# Decoded cardiopoietic cell secretome linkage to heart repair biosignature

**DOI:** 10.1093/stcltm/szae067

**Published:** 2024-09-11

**Authors:** Armin Garmany, D Kent Arrell, Satsuki Yamada, Ryounghoon Jeon, Atta Behfar, Sungjo Park, Andre Terzic

**Affiliations:** Department of Cardiovascular Medicine, Mayo Clinic, Rochester, MN, United States; Center for Regenerative Biotherapeutics, Mayo Clinic, Rochester, MN, United States; Marriott Heart Disease Research Program, Mayo Clinic, Rochester, MN, United States; Mayo Clinic Alix School of Medicine, Regenerative Sciences Track, Mayo Clinic Graduate School of Biomedical Sciences, Mayo Clinic, Rochester, MN, United States; Department of Cardiovascular Medicine, Mayo Clinic, Rochester, MN, United States; Center for Regenerative Biotherapeutics, Mayo Clinic, Rochester, MN, United States; Marriott Heart Disease Research Program, Mayo Clinic, Rochester, MN, United States; Department of Cardiovascular Medicine, Mayo Clinic, Rochester, MN, United States; Center for Regenerative Biotherapeutics, Mayo Clinic, Rochester, MN, United States; Marriott Heart Disease Research Program, Mayo Clinic, Rochester, MN, United States; Section of Geriatric Medicine & Gerontology, Department of Medicine, Mayo Clinic, Rochester, MN, United States; Department of Cardiovascular Medicine, Mayo Clinic, Rochester, MN, United States; Center for Regenerative Biotherapeutics, Mayo Clinic, Rochester, MN, United States; Marriott Heart Disease Research Program, Mayo Clinic, Rochester, MN, United States; Department of Cardiovascular Medicine, Mayo Clinic, Rochester, MN, United States; Center for Regenerative Biotherapeutics, Mayo Clinic, Rochester, MN, United States; Van Cleve Cardiac Regenerative Medicine Program, Mayo Clinic, Rochester, MN, United States; Department of Physiology and Biomedical Engineering, Mayo Clinic, Rochester, MN, United States; Department of Cardiovascular Medicine, Mayo Clinic, Rochester, MN, United States; Center for Regenerative Biotherapeutics, Mayo Clinic, Rochester, MN, United States; Marriott Heart Disease Research Program, Mayo Clinic, Rochester, MN, United States; Department of Cardiovascular Medicine, Mayo Clinic, Rochester, MN, United States; Center for Regenerative Biotherapeutics, Mayo Clinic, Rochester, MN, United States; Marriott Heart Disease Research Program, Mayo Clinic, Rochester, MN, United States; Department of Molecular Pharmacology & Experimental Therapeutics, Mayo Clinic, Rochester, MN, United States; Department of Medical Genetics, Mayo Clinic, Rochester, MN, United States

**Keywords:** cardiopoiesis, heart failure, infarction, machine learning, paracrine, proteomics, secretome, systems biology, stem cells, biotherapy, regenerative

## Abstract

Cardiopoiesis-primed human stem cells exert sustained benefit in treating heart failure despite limited retention following myocardial delivery. To assess potential paracrine contribution, the secretome of cardiopoiesis conditioned versus naïve human mesenchymal stromal cells was decoded by directed proteomics augmented with machine learning and systems interrogation. Cardiopoiesis doubled cellular protein output generating a distinct secretome that segregated the conditioned state. Altering the expression of 1035 secreted proteins, cardiopoiesis reshaped the secretome across functional classes. The resolved differential cardiopoietic secretome was enriched in mesoderm development and cardiac progenitor signaling processes, yielding a cardiovasculogenic profile bolstered by upregulated cardiogenic proteins. In tandem, cardiopoiesis enhanced the secretion of immunomodulatory proteins associated with cytokine signaling, leukocyte migration, and chemotaxis. Network analysis integrated the differential secretome within an interactome of 1745 molecules featuring prioritized regenerative processes. Secretome contribution to the repair signature of cardiopoietic cell-treated infarcted hearts was assessed in a murine coronary ligation model. Intramyocardial delivery of cardiopoietic cells improved the performance of failing hearts, with undirected proteomics revealing 50 myocardial proteins responsive to cell therapy. Pathway analysis linked the secretome to cardiac proteome remodeling, pinpointing 17 cardiopoiesis-upregulated secretome proteins directly upstream of 44% of the cell therapy-responsive cardiac proteome. Knockout, in silico, of this 22-protein secretome-dependent myocardial ensemble eliminated indices of the repair signature. Accordingly, in vivo, cell therapy rendered the secretome-dependent myocardial proteome of an infarcted heart indiscernible from healthy counterparts. Thus, the secretagogue effect of cardiopoiesis transforms the human stem cell secretome, endows regenerative competency, and upregulates candidate paracrine effectors of cell therapy-mediated molecular restitution.

## Graphical Abstract

**Graphical Abstract:**
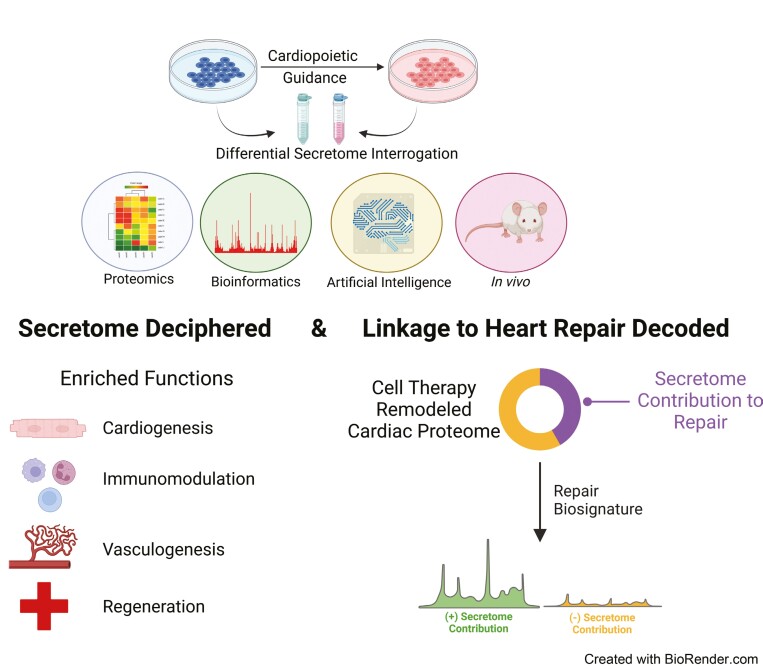

**Significance Statement**

Cardiopoietic cell-based therapy has reached advanced clinical testing for heart failure. While cell protein output is considered contributory to heart repair, the cardiopoietic influence on secreted proteins (‘secretome’) is unknown. A cardiopoiesis triggered secretome overhaul across protein classes imparted cardioregenerative and immunomodulatory enrichment. Within cardiopoietic cell-treated infarcted hearts, the secretome was linked to a repair signature-critical subproteome. This study supports a secretome-dependent contribution to cell therapy meriting further assessment of protein-based regeneration.

## Introduction

Stem cell therapy options for heart failure treatment are evolving, enhanced by optimization strategies.^1-7^ Inherent developmental programming or imposition of lineage guidance cues exemplify biotherapeutic approaches aimed at improved outcomes.^8,9^ Case in point, cardiopoietic conditioning converts a naïve adult stem cell phenotype into a cardiopoietic counterpart yielding augmented cardiorestorative aptitude.^10^ Derived cardiopoietic cells feature upregulated and nuclear translocated cardiac transcription factors, with expression levels correlating to therapeutic efficacy.^11^ Cardiopoietic cellular biotherapy has reached clinical development stages with documented safety and signs of efficacy in heart failure patient populations.^12,13^ Notably, the therapeutic benefit outlasts cell retention postdelivery, suggesting that cardiopoietic cell therapy involves a paracrine contribution.^10,14,15^

Paracrine activity is attributed to the ‘secretome’, the set of molecules secreted by stem cells and implicated in regenerative processes in heart disease.^16-18^ In the context of cardiopoietic cell therapy, participation of the cell protein output is inferred from correlating secretome profiles with the extent of reparative benefit.^19^ In fact, tailoring secretome bioactivity has been identified as a regenerative refinement resource.^20^ Further study of the impact of cardiopoiesis on the secretome protein makeup and its linkage to the cell-treated organ response is thus warranted.

To resolve the complexity of the secreted protein ensemble at high precision, a systems proteomics^21^ approach herein charted secretome composition and characteristics induced by cardiopoietic conditioning of human adult mesenchymal stromal cells. Secreted protein abundance, identities, and expression patterns were compared before and after cardiopoietic guidance. The unbiased strategies of systems biology provided inclusive, multiparametric means to map the cardiopoiesis-dependent secretome architecture and associated properties. With a cardiopoiesis-endowed regenerative constitution, the resolved differential secretome was linked to the repair biosignature of cardiopoietic cell-treated infarcted hearts.

## Materials and methods

### Cardiopoietic induction

Human bone marrow-derived mesenchymal stromal cells (Gibco A15652), were cultured in advanced minimal essential media (AMEM) supplemented with 5% platelet lysate, 1% Glutamax, and 1 IU/mL heparin.^22^ Following expansion, cultured cells were randomized into naïve, unconditioned (Pre), or cardiopoiesis-conditioned (Post) groups. Cardiopoietic induction was carried out for 5 days using a recombinant growth factor-based cocktail.^23^ Specifically, induction of cardiac-specific transcription factors was achieved with the inclusion of transforming growth factor-β (2.5 ng/mL), activin A (10 ng/mL), and bone morphogenic protein-4 (5 ng/mL). Nuclear translocation of cardiac transcription factors was promoted by basic fibroblast growth factor (5 ng/mL) and insulin-like growth factor 1 (50 ng/mL). Cell cycle progression was maintained with the gp130 agonist cardiotrophin (1 ng/mL) and thrombin (1 unit/mL). The applied cocktail was supplemented with the cardiogenic molecule cardiogenol (100 nM), heparin (1000 IU), and 2.5% platelet lysate. Finally, cells were cultured in AMEM supplemented with 2.5% platelet lysate, 1% Glutamax, and heparin (1000 IU) for an additional 5 days.^10,24^

### Directed proteomics of secreted proteins

Pre and Post cell cohorts, seeded at ≈1 × 10^6^ cells per 100 mm dish, were washed twice with phosphate-buffered saline (PBS) and cultured 48 hours in AMEM without platelet lysate. Collected conditioned media was centrifuged (1000 × *g*, 10 minutes), and the supernatant separated. Supernatant and cell pellet protein quantification were carried out using a bicinchoninic acid assay. Supernatant aliquots (200 μL) were dialyzed twice against PBS (300 mL per sample) for 3 hours at 4 °C. The secreted protein output (“secretome”) underwent directed proteomics and resolved 1966 unique proteins by protein array (RayBio L-Series Human Antibody Array 2000 Glass Slide Kit). Protein fluorescence intensities were normalized to cell number using quantified protein content of cell pellets.^19^ Data reliability was assured by preset quality control criteria^25^ that required analyzed specimens to contain only a limited (<1.5 times the interquartile range) number of outlier proteins (≥2 resistant Z scores).

### Secretome composition assessment

Within the resolved secretome, molecular function and biological process frequencies were assessed using the Protein ANalysis THrough Evolutionary Relationships (PANTHER) classification system.^26^ Derived protein functional classes were clustered into superfamilies, and categorical dynamics visualized by alluvial plot using the R (v4.1.3) package ggalluvial.

### Secretome output discrimination

Secretome outputs from Pre and Post cell groups underwent principal component analysis for dimension reduction, with group attribution accomplished using the unsupervised machine learning algorithm K-means clustering. The R ellipse package calculated confidence intervals (CI) for each cluster with the 95% CI center defined as the cluster mean. Clustering was independently verified by supervised random forest classification and a support vector machine model using a polynomial kernel. Accuracy was tested with out-of-bag internal cross-validation, 3-fold cross-validation, and leave-one-out cross-validation in the randomForest and caret R packages.^27,28^ To assess the respective contribution of individual proteins to group segregation, first and second principal component loading plot values were derived for each detected protein. Loading plot feature selection was validated by the artificial intelligence-powered Boruta algorithm (Boruta R package) using the 20 molecules that presented with the highest loading values.^29^ Plots were generated with the R package ggplot2.

### Secretome differential protein expression

Inferential statistics for each secretome protein were conducted across cell groups based on array mean fluorescence intensity. A 2-sided Mann-Whitney test, Welch’s *t* test, or Student’s *t* test were used as appropriate, following consideration of normality and homogeneity of variance assessed by Wilk-Shapiro and *F* tests, respectively. The Benjamini-Hochberg false discovery rate correction method was applied across comparisons. Differential expression was defined as a |Log_2_ Ratio| ≥ 1 (Post vs Pre) and a false discovery rate < 0.05.

### Systems interrogation and interactome synthesis

Systems interrogation was carried out at the differential secretome level for proteins with assigned human UniProt identifiers and broadened to the expanded molecular network neighborhood.^30^ Enrichment analysis of secretome Gene Ontology biological processes was conducted with the ViSEAGO R package^31^ using Fisher’s test in conjunction with the topGO elimination algorithm R package to reduce redundancy and compensate for Gene Ontology hierarchical interdependencies.^32^ To compute semantic similarity for enriched biological processes the Wang method was used, followed by Ward’s method for hierarchical agglomerative clustering to extract process macroclusters,^31,33^ with harmonic mean *P*-values calculated for each cluster.^34,35^ Ingenuity Pathway Analysis (v70750971) was applied to identify enriched biological functions and network interactions. To assess the secretome network landscape,^36,37^ interactome synthesis was implemented with the circlize R package and the Cytoscape (v3.8.2) Bioinformatic Network Gene Ontology (BiNGO) tool used to identify overrepresented biological processes within the derived interactome.^19,38^ Enrichment was defined as a Benjamini-Hochberg corrected *P*-value < .05 using a hypergeometric test. KEGG pathway analysis was conducted in R (v4.4.1) with the clusterProfiler package^39^ using a hypergeometric test. KEGG pathways with a Benjamini and Hochberg corrected *P*-value < .05 were considered overrepresented. KEGG pathways were clustered with Biological Relationships of Information Transmitting Entities (BRITE) hierarchies to derive harmonic mean *P*-values for each class.^34^

### Myocardial infarction and cell delivery

Under protocols approved by the Mayo Clinic Institutional Animal Care and Use Committee, adult (2-3 months old) athymic nude mice underwent aseptic thoracotomy and left anterior descending coronary artery ligation (with a 9-0 monofilament nylon suture). Myocardial infarction was confirmed by ST-elevation and anterior wall hypokinesis using in-procedure electrocardiography and post-procedure echocardiography, respectively. Four weeks after ligation, single-housed and ad libitum-fed mice (*n* = 54) with confirmed anterior infarction and left ventricular (LV) ejection fraction <50% were randomized to receive, through epicardial injections, either vehicle (*n* = 13 mice), 600 000 naïve MSCs (*n* = 8 mice), or 600 000 cardiopoietic cells (*n* = 33 mice) across 5-6 sites (15 μL total injected volume) at the peri-infarct border. Treatment efficacy was evaluated 1-month postintervention by transthoracic echocardiography (Vevo 3100 imaging system with MX400 transducer, VisualSonics) in an investigator-blinded fashion.^40,41^ Left ventricular ejection fraction was calculated per the American Society of Echocardiography as follows: 100 × (LV end-diastolic volume − LV end-systolic volume)/LV end-diastolic volume with volume measurements abstracted from 2D images.^42^ Group comparisons were made using repeated-measures 2-way ANOVA followed by post hoc testing with a 2-sided Wilcoxon signed-rank test or paired *t* test based on assessment of normality and homogeneity of variance with the Wilk-Shapiro and *F* test, respectively. Response to therapy was defined as an improvement of ≥4% in LV ejection fraction in line with established clinical criteria.^41,43^ Responder percentage was compared using Fisher’s exact test followed by post hoc testing with a pairwise Fisher’s exact test.

### Undirected heart proteome assessment

To assess the impact of cardiopoietic cell treatment on the infarcted cardiac proteome, undirected proteomics was carried out on excised ventricles from cell- and vehicle-treated infarcted murine hearts at 1-month postintervention. Noninfarcted hearts from age-matched mice also underwent proteomic assessment and served as a reference. Following sodium dodecyl sulfate-polyacrylamide gel electrophoresis of ventricular protein extracts,^41^ resolved proteins were reduced, digested, and tryptic peptides extracted for untargeted nano-flow liquid chromatography-electrospray tandem mass spectrometry.^35^ Protein abundance was quantified across healthy (noninfarcted), infarcted with sham treatment, and infarcted with cell treatment hearts. Differential expression was conducted by a 2-sided ANOVA with a Gaussian link function using R. Proteins with a |Log_2_ Ratio| ≥ 1 (cell-treated/sham-treated) and false discovery rate < 0.05 were considered differentially expressed.^40^ The heart proteome was evaluated at systems level with Ingenuity Pathway Analysis to identify the overrepresented cardiovascular disease and cardiac adverse functions with a *P* < .05, while in parallel, gene set enrichment analysis with the clusterProfiler package^39^ identified enriched Gene Ontology annotations with a *P* < .05.

### Mapping cardiopoietic secretome to infarcted proteome restoration

The relationship of the resolved differential secretome with the cell therapy remodeled infarcted myocardial proteome was interrogated at 3 levels. First, Ingenuity Pathway upstream regulator analysis was used to identify potential paracrine effectors within the secretome with an overlap *P*-value of <.05. Expression levels of candidate paracrine effectors in the secretome of cardiopoietic cells providing therapeutic response were confirmed using directed proteomics. To this end, cardiopoietic cells manufactured from bone marrow MSCs of ischemic heart failure patients undergoing clinical testing were cultured.^11,19^ Conditioned media was collected and scanned with the RayBiotech Human L507 Array protein chip. Efficacy readout followed predefined trial endpoints.^12,19^ Second, the My Pathways tool in Ingenuity Pathway Analysis was used to identify secretome-dependent and independent protein subsets in the cell-responsive myocardial proteome. For this purpose, cell treatment-responsive myocardial proteins that linked to upstream regulators were mapped and proportional allocation was visualized by alluvial plot using the R package ggalluvial. Third, the secretome-dependent myocardial proteome was knocked out *in silico* to assess, using Ingenuity Pathway Analysis, secretome contribution to repair. Knockout was conducted by factoring out the secretome-dependent protein subset from the cell-responsive myocardial proteome.

## Results

### Cardiopoiesis stimulates stem cell protein secretion

**Figure 1.**
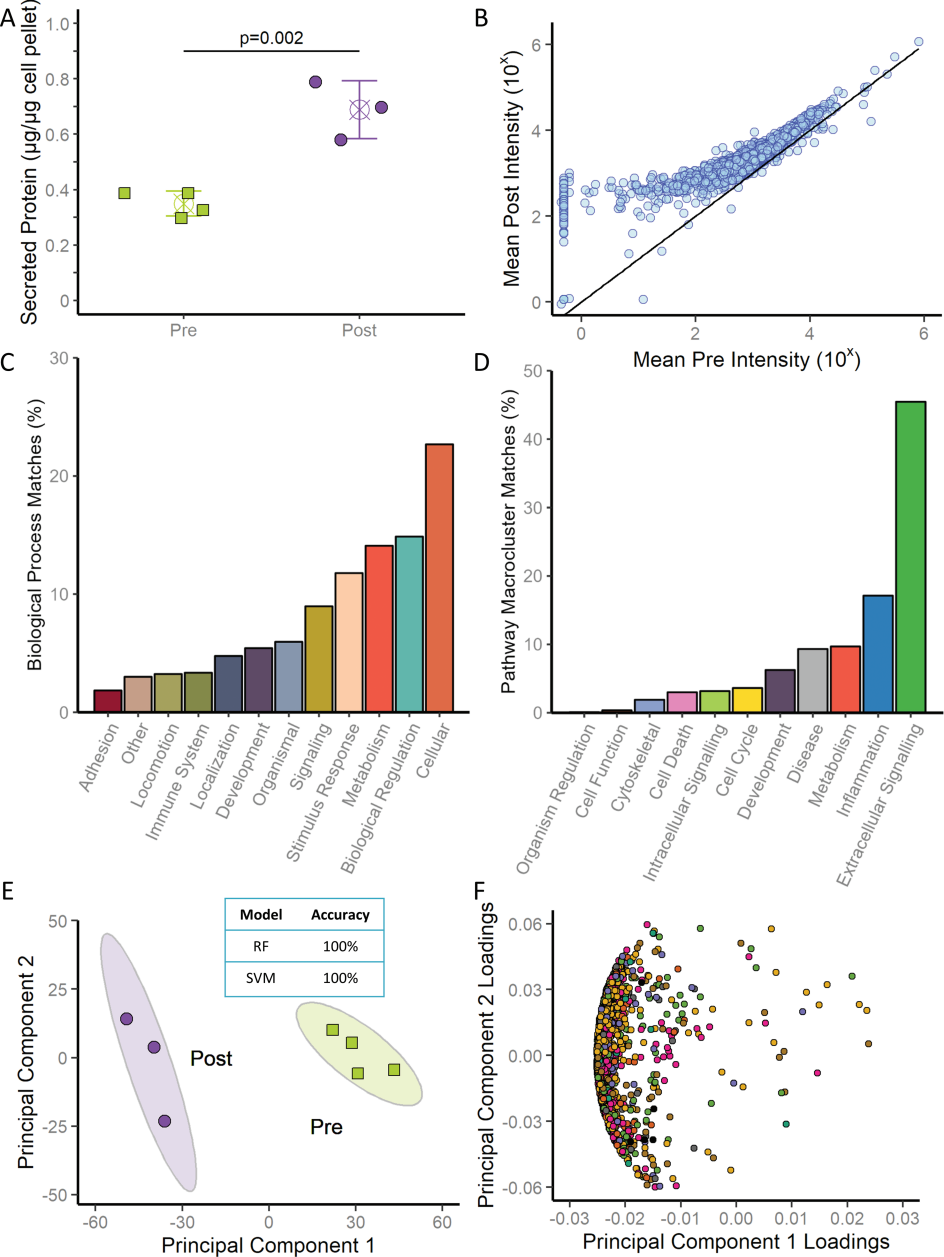
Cardiopoiesis alters the human stem cell secretome. (A) Lineage-specifying cardiopoiesis doubled the protein content secreted by primed (Post) versus naïve (Pre) human mesenchymal stromal cells. Values represent mean (X) ± standard deviation (error bars). (B) The protein array quantified 1966 proteins in the secreted cell output of Pre and Post states. (C) The resolved secretome represented a diversity of Gene Ontology Biological Processes as determined by Protein ANalysis THrough Evolutionary Relationships (PANTHER), which reflected the total human proteome. (D) A multitude of PANTHER pathways consolidated into macroclusters distinguished the secretome with extracellular signaling preeminent. (E) Secretomes prior and following cardiopoiesis conditioning (Pre and Post) segregated on principal component analysis, concordant with K-means clustering. Colored ellipses outline 95% CIs. (E Inset) Random forest (RF) and support vector machine (SVM) models classified conditioning status with 100% accuracy. (F) Principal component loading plots yielded low values for individual secreted proteins, in line with cohort segregation reliant on the secretome as a whole. Colors represent 9 protein class clusters assigned using PANTHER: blue = cell Function, orange = DNA maintenance and transcription, purple = immunomodulation, pink = metabolism, green = protein synthesis and regulation, yellow = signaling, brown = structure, gray = transport, and black = unclassified.

Cardiopoietic conditioning of human adult bone marrow-derived mesenchymal stromal cells with an established recombinant growth factor cocktail promoted protein secretion, doubling the secreted protein output (*t* = 5.93, 95% CI [0.19, 0.49], *P* = .002 Post- versus Pre- conditioning; Figure 1A). Individual protein abundance was determined across 1966 proteins resolved by directed proteomics (Figure 1B). Measured proteins spanned diverse biological process clusters representative of the human proteome (Figure 1C). Consistent with the nature of secreted proteins, extracellular signaling comprised 45% of represented pathways (Figure 1D). Thus, cardiopoiesis stimulates human mesenchymal stromal cell secretory activity.

### Secretome output distinguishes cell state

The cardiopoiesis-induced secretome profile was distinct from that produced by unconditioned, naïve mesenchymal stromal cells (Figure 1E). Principal component analysis separated secreted protein outputs, with K-means clustering—an unsupervised machine learning algorithm—segregating secretomes based on cardiopoiesis conditioning status (Figure 1E). Random forest and support vector machine models, 2 distinct supervised artificial intelligence classifiers, independently validated the distinction of secretomes generated before (Pre) versus after (Post) cardiopoietic conditioning (Figure 1E inset). Notably, discrimination relied on the totality, rather than on a limited set, of secreted proteins. Indeed, the corresponding loading plot—which reflects the relative contribution of each protein—yielded low squared factor loadings for all proteins, signifying limited contribution by individual proteins to the observed secretome distinction (Figure 1F). In fact, discrete protein subsets (20 top proteins ranked by factor loading) were insufficient in distinguishing cardiopoiesis conditioned from unconditioned secretomes. Rather, reliance on the totality of the secretome for segregation was needed, documented by modest mean decrease accuracy scores in random forest feature selection, and further established by the artificial intelligence Boruta algorithm rejecting or not confirming individual candidate proteins for feature selection (Supplementary Table S1). Thus, cardiopoiesis determines secretome identity across the resolved mesenchymal stromal cell protein output.

### Cardiopoietic secretome ensemble adopts cardiogenic imprint

**Figure 2.**
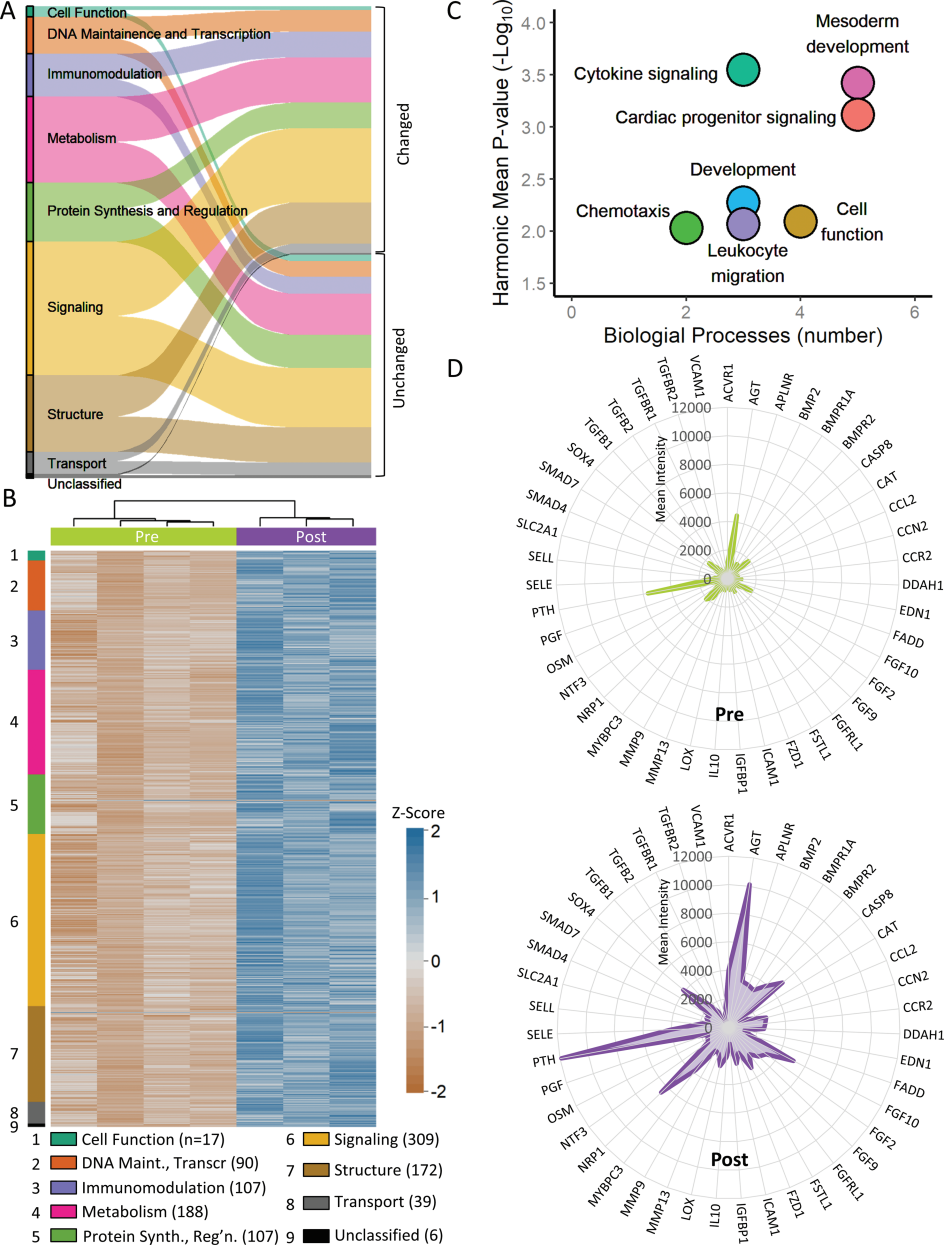
Cardiopoiesis endows a cardiovasculogenic secretome identity. (A) As shown by alluvial plot, cardiopoiesis overhauled all 9 protein class clusters assigned to the adult stem cell secretome by Protein ANalysis THrough Evolutionary Relationships (PANTHER). Extent of change ranged from 32% to 60% of proteins across individual classes with 53% of the entire secretome remodeled. (B) Agglomerative clustering of Pre versus Post secretomes, based on 1035 differentially expressed proteins (with *z*-score transformed expression values), revealed a distinct cardiopoiesis secretome identity. Proteins were grouped by PANTHER class clusters. (C) The differential cardiopoietic secretome, evaluated by ViSEAGO, was enriched in 25 Gene Ontology Biological Processes clustered into 7 classes by semantic similarity. Prominent enrichment of mesoderm development and cardiac progenitor signaling characterized the cardiopoiesis secretome signature. (D) Ingenuity Pathway Analysis-enriched cardiovasculogenic functions connected to 43 proteins upregulated by cardiopoiesis, exposing the cardiovasculogenic profile of the cardiopoietic secretome.

In the resolved secretome, all assigned functional categories were substantially impacted by cardiopoiesis, with expression altered for one-third or more proteins within each annotation (Figure 2A). Proteins related to cell function (39% altered), DNA transcription/maintenance (58%), immunomodulation (60%), metabolism (52%), protein synthesis and regulation (44%), signaling (56%), structure (54%), and transport (42%) were specifically influenced by cardiopoietic induction. Overall, 53% of the resolved secretome was differentially expressed (2-fold, false discovery rate < 0.05) in response to cardiopoietic guidance (Figure 2A) with 1033 proteins upregulated and 2 downregulated (Figure 2B). Hierarchical agglomerative clustering of the differential secretome confirmed segregation in accordance with cardiopoietic cell induction status (Figure 2B). Notably, 7 biological process clusters distinguished the differential cardiopoietic secretome (Figure 2C), indicating that beyond quantitative restructuring the secretome underwent qualitative metamorphosis. Namely, mesoderm development, cardiac progenitor signaling, cytokine signaling, cell function, development, leukocyte migration, and chemotaxis were prominently ranked by degree of enrichment and number of associated biological processes (Figure 2C). The enrichment of mesoderm development/development and cardiac progenitor signaling clusters was fortified by 141 differentially expressed proteins contributing to 14% of the total change (Figure 2C). The elicited secretome featured enriched cardiovasculogenic functions underpinned by a synchronous upregulation of 43 proteins (Figure 2D). Concomitantly, the secretome was enriched in immunomodulatory biological process clusters (namely, cytokine signaling, chemotaxis, and leukocyte migration) underpinned by 188 proteins upregulated in response to cardiopoietic conditioning and contributing to 18% of the differential secreted protein output (Figure 2C). Likewise, signaling and immunomodulatory pathway clusters were independently prioritized (Supplementary Figure S1A), underpinned by 27 enriched KEGG pathways (Supplementary Figure S1B). Thus, cardiopoiesis inculcates a nonstochastic molecular pattern to the secretome output, congruent with an endowed cardioregenerative and immunomodulatory potential.

### Interactome synthesis prioritizes regenerative enrichment

**Figure 3.**
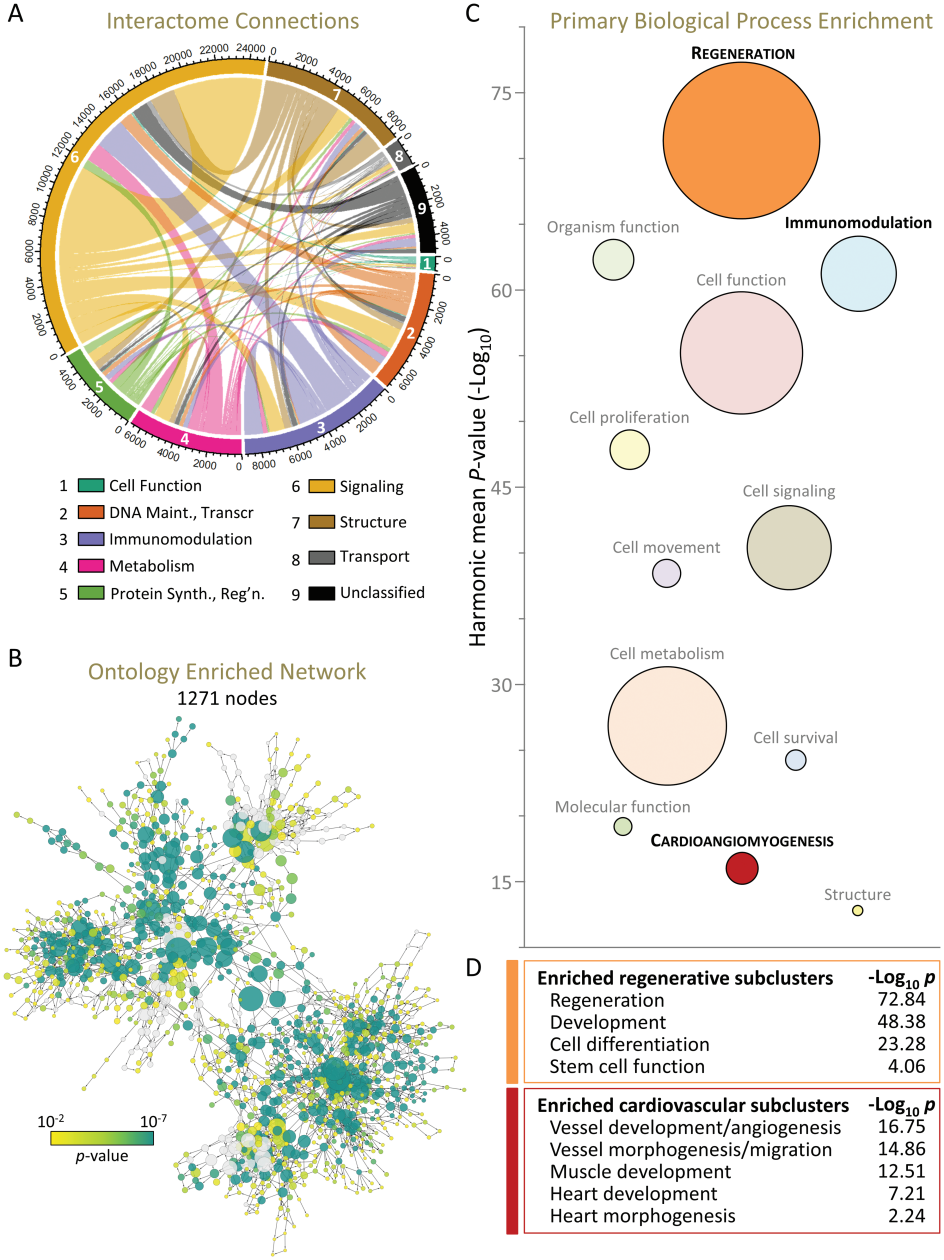
Interactome synthesis prioritizes regenerative traits. (A) Ingenuity Pathway Analysis yielded a 1745-node interactome from the cardiopoietic secretome and its interacting molecular neighborhood, comprising a total of 34 281 edges. Interactome substituents were classified into Protein ANalysis THrough Evolutionary Relationships protein classes with intergroup interactions visualized by chord diagram. Colors represent the class of origin node for each interaction and values represent the number of connections for each protein class, totaling 68 562 (2 per edge). (B) In Cytoscape, the network was assessed for Gene Ontology enrichment, yielding a 1271-node hierarchical network containing 1162 overrepresented biological processes (*P* < .01). (C) Process term similarities enabled the reduction of ontological complexity to 12 macroclusters (sized proportionally to the number of contained processes, with significance centered at the harmonic mean *P*-value of cluster-contained processes), confirming the enrichment of cardioangiomyogenesis (red) and immunomodulation (blue) while revealing prioritization of regeneration (orange). (D) Regeneration, development, cell differentiation, and stem cell function underpinned the regeneration cluster. Vascular development and morphogenesis, myogenesis, and heart development and morphogenesis supported the enrichment of the cardioangiomyogenic cluster.

Network assembly positioned the cardiopoietic secretome within a broader neighborhood comprising 1745 constituent molecules integrated through 34 281 pairwise interactions (Figure 3A). Accounting for over one-third of network connections, the prominence of signaling proteins in the secretome was reiterated in the derived interactome (Figure 3A). Functional interrogation of the secretome network yielded an ontological hierarchy of 1271 biological processes of which 1162 were overrepresented (Figure 3B). Spanning 12 macroclusters (Supplementary Table S2), these processes independently reiterated the cardiovasculogenic and immunomodulation signatures inherent to the differential secretome (Figure 3C). Beyond validating core secretome properties, consideration of the affiliated interactome unmasked ‘Regeneration’ as the predominant enriched ontology macrocluster delineating acquired secretome functionality through neighborhood assembly (Figure 3C). Collectively, 9 distinct subclusters within Regeneration and Cardioangiomyogenesis anchored a cardiopoiesis instilled prioritization of cardiovascular regeneration (Figure 3D). Thus, construction and assessment of the cardiopoiesis modified interactome affirmed secretome cardiovasculogenic and immunomodulatory enrichment, and further exposed regenerative dominance at network systems level.

### Cardioreparative competence validated in infarcted hearts

**Figure 4.**
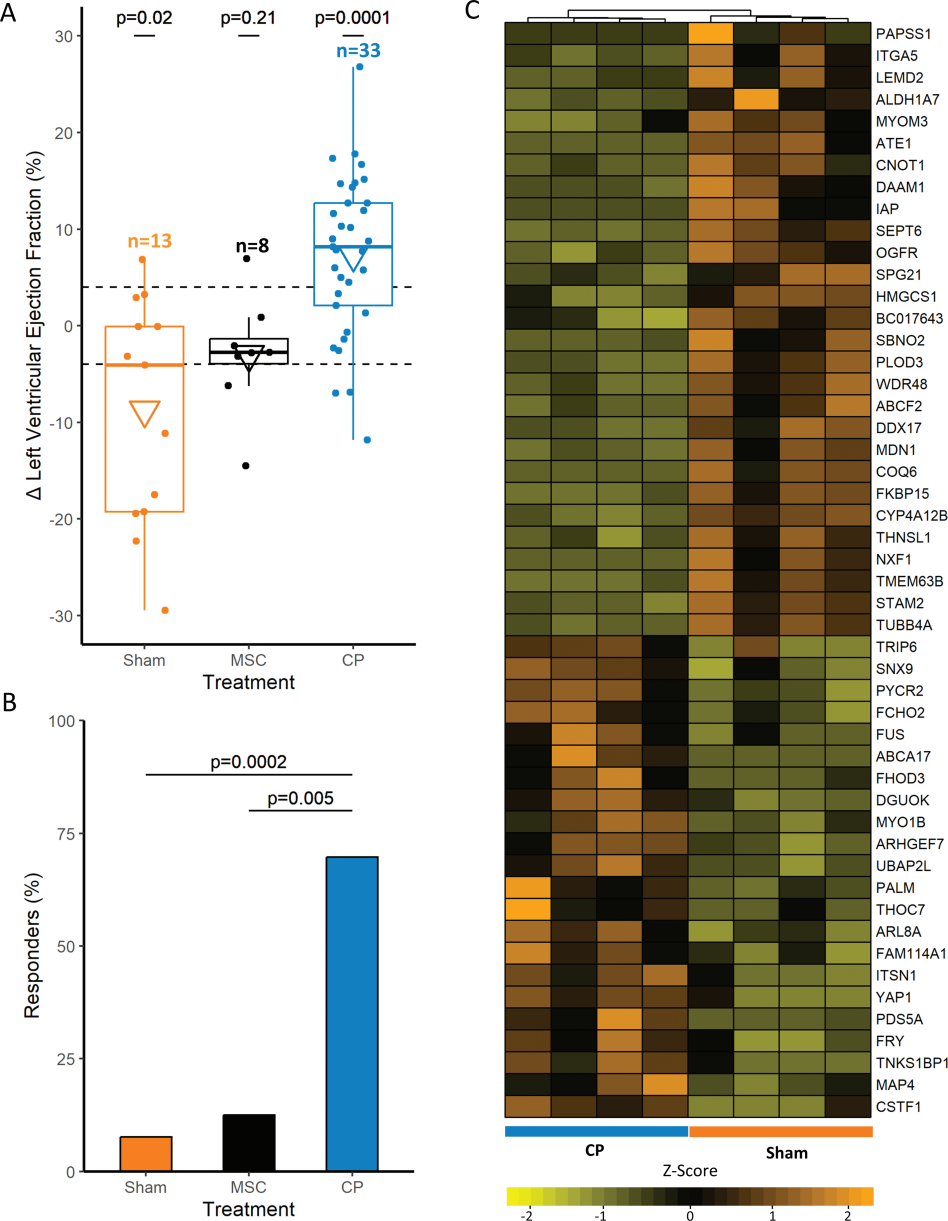
Cardiopoietic cell therapy promotes functional and molecular restitution of infarcted murine hearts. (A) In vivo efficacy following intramyocardial cell delivery was evaluated in the setting of murine chronic ischemic cardiomyopathy at 1-month post-treatment. Epicardial transplantation of cardiopoietic cells (CP), but not of naïve mesenchymal stromal cells (MSC) or vehicle (Sham), demonstrated significantly improved cardiac performance 1-month after cell delivery, as assessed by change in left ventricular ejection fraction (ΔLVEF %) ± standard error of the mean. Box plots represent 75th percentile, median, and 25th percentile with whiskers showing maximum and minimum values 1.5 times the interquartile range above and below the 75th and 25th percentiles, respectively. Triangles represent mean ΔLVEF %. (B) Beneficial therapeutic response was defined as ≥4% improvement in ejection fraction 1-month after therapy. CP cell therapy exhibited a significantly greater proportion of responders than observed for MSC or Sham treatment. (C) Molecular response of infarcted left ventricles to CP cell therapy was evaluated by undirected mass spectrometric proteome comparison to vehicle-treated (Sham) infarcted hearts. Fifty proteins were found to comprise the CP cell treatment-responsive myocardial proteome (|Log_2_ ratio| ≥ 1, false discovery rate < 0.05), visualized by agglomerative clustering of *z*-score transformed expression values, with proteins listed by their Human Genome Organization gene symbol.

The proficiency of cardiopoiesis-conditioned human mesenchymal stromal cells was evaluated in chronically infarcted murine hearts in vivo. Following cell therapy, no animals demonstrated systemic toxicity, adverse events, or mortality throughout follow-up. Functional impact was determined 1-month after therapy by documenting change in left ventricular ejection fraction, a parameter reflecting cardiac performance. Beneficial response was defined as ≥4% improvement in ejection fraction. Cardiopoietic cell treatment resulted in ejection fraction improvement (V-statistic = 62, *P* < .0001) with an average increase of 7 ± 1% (±standard error of the mean; Figure 4A). In contrast, treatment with unconditioned, naïve cells resulted in no benefit (−3 ± 2%, *t* = 1.39, 95% CI [−2.10, 8.04], *P* = .21), and sham treatment (vehicle) progressed into cardiac functional decline (−9 ± 3%, *t* = 2.69, 95% CI [1.66, 15.82], *P* = .02). Overall, 70% of hearts treated with cardiopoietic cells responded favorably, significantly exceeding the 12% (*P* < .05) and 8% (*P* < .01) responders observed in unconditioned cell and sham treatments, respectively (Figure 4B). The efficacy of functional recovery at organ level was supported by cardiopoietic cell treatment-induced molecular remodeling of the infarcted myocardium (Figure 4C). In total, 50 proteins in the resolved myocardial proteome responded to cardiopoietic cell treatment (Figure 4C). Thus, cardiopoietic cell therapy promotes functional recovery underpinned by molecular restitution of infarcted hearts.

### Withdrawal of secretome influence erases heart repair biosignature

**Figure 5.**
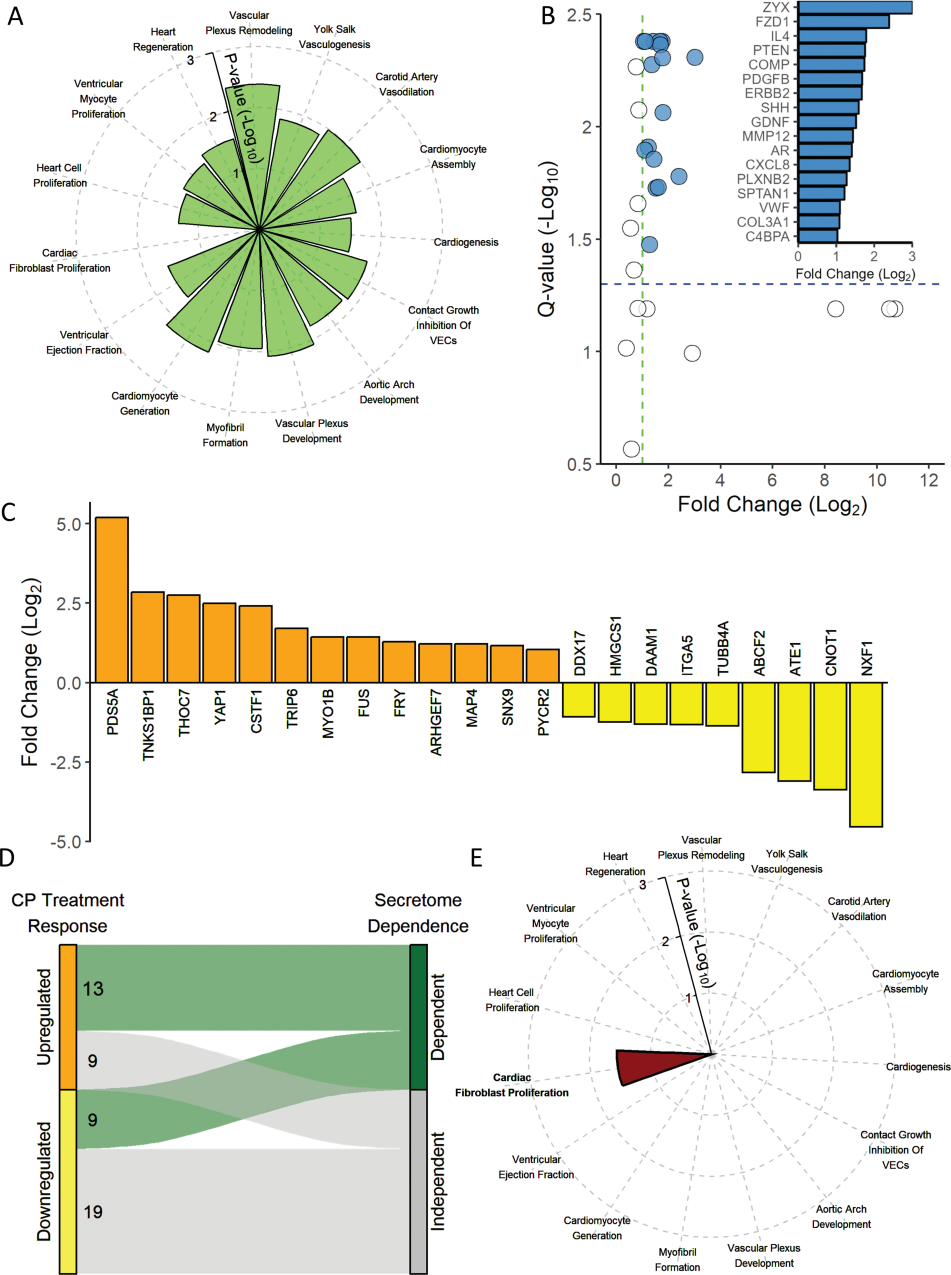
Removal of secretome-dependent contribution abrogates the cardioregenerative imprint of cell therapy. (A) A significantly enriched cardioregenerative signature (green) characterized the cell therapy remodeled myocardial proteome, evaluated by cardiovascular system diseases and toxicology functions in Ingenuity Pathway Analysis (IPA). (B) IPA regulator analysis of the 50 proteins that were cell therapy responsive in the infarcted heart identified 30 potential upstream effectors present in the measured stem cell secretome, of which 17 were upregulated (|Log_2_ ratio| ≥ 1, false discovery rate < 0.05) in response to cardiopoiesis (blue; inset with proteins listed by Human Genome Organization gene symbol). (C) These regulators linked to 13 upregulated (orange) and 9 downregulated (yellow) cardiac proteins (listed by Human Genome Organization gene symbol) responsive to cardiopoietic cell therapy, (D) delineating secretome-dependent (green) and secretome-independent (gray) subsets within the cell therapy-responsive, infarcted myocardial proteome. (E) *In silico* knockout of the secretome-dependent subset of the remodeled infarcted proteome eliminated the enriched cardioregenerative signature upon IPA reassessment of cardiovascular system diseases and toxicology functions, leaving a profibrotic, anti-regenerative hallmark (red).

The cardiopoietic cell treatment-remodeled myocardial proteome was overrepresented in cardiovasculogenic and regenerative functions identified by pathway enrichment analysis (Figure 5A) and further supported by gene set enrichment analysis of Gene Ontology annotations (Supplementary Figure S2). To assess secretome linkage to cell therapy outcome, the cardiopoietic secretome was screened by upstream regulator analysis for candidate paracrine effectors of myocardial molecular remodeling. A total of 30 regulators were identified, with 17 upregulated in response to cardiopoietic induction (Figure 5B). Independent expression profiling of 8 of these candidate regulators in secretomes of patient-derived-cells revealed increased secretion of all 8 of these proteins in the secretome of cells providing therapeutic benefit, with 7 exhibiting >2-fold upregulation (Supplementary Figure S3). These cardiopoietic secretome proteins directly linked downstream to 22 myocardial proteins responsive to cardiopoietic cell treatment (Figure 5C), revealing that 44% of the remodeled cardiac proteome was secretome dependent (Figure 5D). Knockout*, in silico,* of the 22-member secretome-dependent subproteome nullified cardioreparative enrichment, exposing a profibrotic remnant (Figure 5E). Thus, the cardiopoietic secretome harbors core contributors to the heart repair signature.

### Secretome-dependent myocardial proteome returns to preinfarction state following therapy

**Figure 6.**
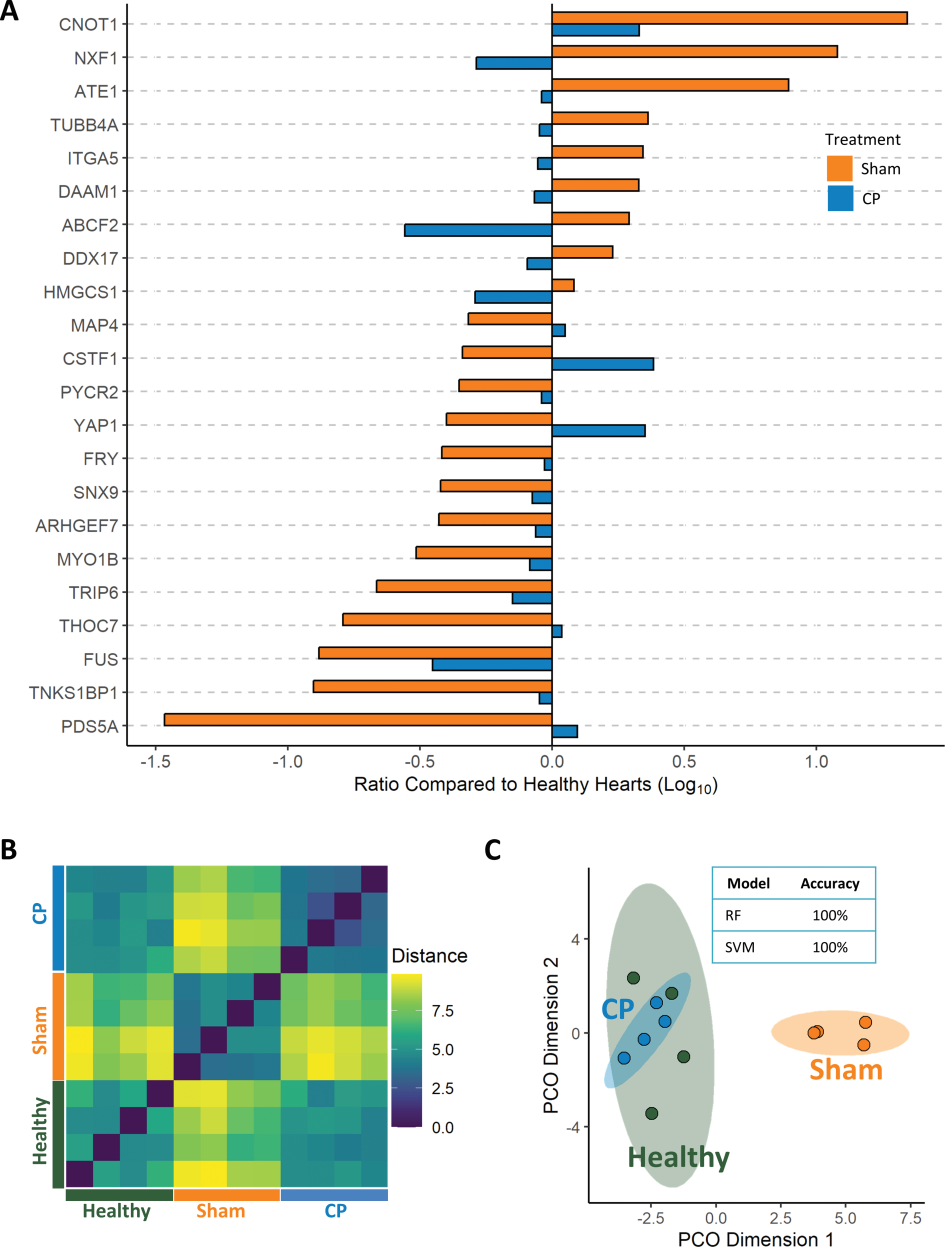
Cell therapy-imposed changes to secretome-dependent subproteome propel infarcted hearts toward a healthy state. (A) The 22 secretome-dependent proteins of the cell therapy-responsive myocardial proteome exhibit changes in protein expression toward healthy levels. (B) Dissimilarity matrix using Euclidean distances of expression levels of the secretome-dependent subproteome revealed greater similarity between healthy hearts and cardiopoietic cell-treated hearts (CP) than either exhibited with sham-treated hearts. (C) Principal coordinates (PCO) analysis documented the equivalence of the secretome-dependent subproteome of cardiopoietic cell-treated (CP) and healthy hearts with overlapping 95% CIs (ellipses), distinct from that of sham-treated hearts. (C inset) Informed by the expression of the 22 secretome-dependent myocardial proteins random forest (RF) and support vector machine (SVM) models classified treatment status with 100% accuracy.

Each protein of the 22-member secretome-dependent subproteome within the infarcted myocardium exhibited, in response to cell therapy, a return in expression toward preinfarction levels (Figure 6A). Expression pattern similarity, assessed by Euclidean distances and visualized by distance matrix, documented a transition of the infarcted subproteome, reaching greater resemblance with the healthy state following therapy (Figure 6B). Dimensional reduction by principal coordinates analysis revealed that the secretome-dependent subproteome within cardiopoietic cell-treated hearts was indistinguishable from the preinfarcted state as documented by overlapping 95% CIs (Figure 6C). In contrast, sham-treated hearts displayed a subproteome distinct from both the cell-treated infarcted hearts and the preinfarcted hearts (Figure 6C). A 100% accuracy in discerning cell-treated from sham-treated infarcted hearts was independently achieved by random forest and support vector machine classifiers (Figure 6C inset), documenting the distinct trajectory of the secretome-dependent subproteome in cell-treated infarcted hearts.

## Discussion

The present study, using directed proteomics, reveals that cardiopoiesis transforms the human adult stem cell secretome. Informed by the secretome expression profile, supervised and unsupervised clustering distinguished secretomes by cardiopoietic conditioning status. The resolved atlas of secreted proteins and associated interactome was characterized by an enrichment of cardioregenerative and immunomodulatory features imposed by cardiopoietic stimuli. Systems interrogation linked the decoded secretome to the proteome of cardiopoietic cell-treated infarcted hearts, unmasking putative effectors of molecular cardiac restitution within the cardiopoietic secretome. Withdrawal of the identified secretome-dependent myocardial targets eliminated the cardiopoietic cell repair biosignature. Observed expression changes of the secretome-dependent subproteome were nonrandom, resetting the molecular profile of cell-treated infarcted hearts toward a healthy state. Thus, cardiopoietic guidance selectively transforms the molecular architecture of the human mesenchymal stromal cell secretome bolstering the inherent cardioregenerative and immunomodulatory substrate.

Cardiopoiesis is an optimizing, lineage-specifying strategy that guides adult stem cells into a cardiorestorative program.^44^ Derived human cardiopoietic cells have shown promise in preclinical studies,^10,14^ and have advanced to late-stage clinical trials demonstrating benefit in patients with chronic heart failure in the setting of myocardial infarction.^45,46^ Characterization of the cardiopoietic cell phenotype has largely been limited to defining its intracellular fingerprint.^11^ Notably, guidance of mesenchymal stromal cells with a cardiopoietic protocol activates phenotypic plasticity promoting cytosolic induction and nuclear translocation of cardiac transcription factors.^23^ The present study delineates the impact of cardiopoietic guidance on the extracellular protein output and infers the contribution of the resolved cardiopoiesis footprint on heart repair.

Specifically, cardiopoietic induction exerted a secretagogue effect doubling the secreted protein content of adult human mesenchymal stromal cells. Previously, an enhanced therapeutic proficiency of neonatal human cardiac progenitor cells has been associated with increased protein secretion when compared to adult counterparts.^47^ Enhanced secretion was accompanied by a distinct secretome composition with decoded proteome identities distinguishing adult human cells post- versus pre-cardiopoietic induction. Probing the secreted protein repertoire relied on an array reflecting the diversity of the total human proteome. The resolved cardiopoietic secretome encompassed 1035 differentially expressed proteins spanning functional classes. Stem cell secretome composition is known to vary based on cell source and age.^48-51^ Here, the quantified impact of a lineage-specifying protocol on secretome identity revealed an overhaul exceeding natural variations ascribed to stem cell type, harvest location, and/or donor.^20,51,52^ Taken together, the cell secretome is thus malleable and a genuine target for modulation by environmental cues.^53-56^

Beyond global revision of the secretome, unbiased systems assessment demonstrated acquired cardiogenic enrichment with prioritization of mesoderm development- and cardiac progenitor signaling-specific biological processes. Proteins linked to cardiovasculogenic and immunomodulatory properties were upregulated, underscoring changes prompted by cardiopoiesis. Notably, proteins targeted by cardiopoietic restructuring matched the identity of bioactive proteins critical to mechanisms linked with therapeutic proficiency.^57^ Angiogenin, fibroblast growth factor-2, insulin-like growth factor-1, interleukin-8, interleukin-10, monocyte chemoattractant protein-1, monokine induced by interferon-gamma, macrophage inflammatory protein-1alpha, placenta growth factor, transforming growth factor beta, and tissue inhibitor of metalloproteinase-2, all contributing to the vasculogenic and immunomodulatory features of mesenchymal stromal cells,^58-67^ were here found significantly upregulated in post- versus pre-cardiopoiesis secretomes. Concordant with the observed secretome expression patterns, cardiopoietic cells stimulate vascular tube formation, promote vascularization, and anti-inflammatory macrophage polarization, beneficial for heart repair.^14,68,69^ Furthermore, the cardiopoietic secretome displayed augmented expression of cardioprotective molecules that characterize secretome profiles of reparative stem cells including insulin-like growth factor-1, transforming growth factor beta, and neuregulin-1.^19,47,70,71^ In fact, neuregulin-1 exemplifies a cardiopoietic secretome-contained protein under clinical trial investigation for the treatment of heart failure.^72^ Upregulated protein changes following cardiopoietic induction were likewise associated with the enrichment of signaling pathways previously established in cardiac repair and regeneration, including Hippo-Yap^73^ and PI3K-Akt signaling.^74^ Extension of the differential secretome map, including the interacting molecular cluster, confirmed cardiovasculogenic and immunomodulatory overrepresentation and further revealed regenerative preeminence. Thus, cardiopoiesis customizes the secretome tapestry inculcating cardioreparative potential through selective upregulation of cardioprotective, immunomodulatory, and vasculogenic protein sets.

Functional recovery of failing hearts is associated with molecular remodeling of the diseased myocardium bestowing a signature of repair.^40,75^ In cardiopoietic cell therapy, the observed improvement in contractile function of infarcted hearts was accompanied by proteome reorganization reflective of a transition from disease toward a predisease state.^19,40^ The present study delineated the secretome-dependent myocardial response to cardiopoietic cell therapy deciphering a subproteome target amenable to restoration to its healthy state. Supporting the potential of a paracrine mode of action,^76^ upstream regulators driving molecular restitution of treated infarcted hearts were pinpointed as inherent to the cardiopoietic secretome. Specifically, the present study identified 30 secretome proteins upstream of the cardiopoietic cell-responsive myocardial subproteome, of which 17 were upregulated by cardiopoietic induction. These cardiopoietic secretome-dependent effectors linked to 22 of the 50 cardiopoietic cell-responsive myocardial proteins implicated in the restoration of an infarcted cell-treated myocardium.^40^ Ablation of the secretome-dependent myocardial protein set eliminated the cardioregenerative signature defining cardiopoietic cell treatment of infarcted tissue, suggestive of a critical contribution to therapeutic outcome. While the complexity of cardiopoietic cell therapy precludes a purely reductionist assessment,^77^ this initial probing of the secretome potential identified molecular candidates for paracrine involvement in cardiopoietic biotherapy. Indeed, targeted knockout of secretome components compromises the therapeutic potential of human cardiac progenitor cells,^47^ underscoring the secretome as a functional regenerative unit.

The therapeutic potential inherent to the secretome has been an area of active investigation,^66,78-82^ prompted by the sustained benefit of adult stem cell treatment outlasting cell retention following delivery into the heart.^10,15^ Indeed, single injection^47,83^ and sustained delivery^84^ of conditioned media collected from adult stem cells improve or preserve ventricular function post-myocardial infarction. Moreover, lineage specification has been used as a conditioning strategy for honing secretome competence in regenerative applications.^13,48^ The present study provides a systems biology-based chart for a presumed secretome-autonomous, acellular contribution to cell therapy benefit.

In summary, this study decodes the cardiopoietic cell secretome at the protein level. Cardiopoiesis reengineered the molecular identity of a cell secretome endowing concentration of cardioregenerative traits. Knockout of the secretome contribution undermined the predicted cardiac repair outcome of cardiopoietic cell treatment, underscoring a potential paracrine contribution to the mode of action. Moreover, secretome-dependent myocardial proteome changes redirected treated infarcted hearts toward a healthy state. Thus, the cardiopoietic cell secretome merits further consideration as a protein-based regenerative biotherapy platform.

## Supplementary Material

Supplementary material is available at *Stem Cells Translational Medicine* online.

szae067_suppl_Supplementary_Table_S1

szae067_suppl_Supplementary_Figures

szae067_suppl_Supplementary_Table_S2

## Data Availability

Proteomic mass spectrometry data are available via the public repository ProteomeXchange with identifier PXD017381.
