# Supplementary material for: Decoded cardiopoietic cell secretome linkage to heart repair biosignature
Source: Stem Cells Transl Med. 2024 Sep 11;13(11):1144–59. doi: 10.1093/stcltm/szae067 (PMC11555478; doi:10.1093/stcltm/szae067)
Supplement: szae067_suppl_Supplementary_Table_S1 [file szae067_suppl_supplementary_table_s1.pdf]

Supplemental Table 1. Boruta Analysis Feature Selection. Secretome proteins with the 20 largest loading values were evaluated via the Boruta algorithm to identify molecules segregating cell outputs before and after cardiopoietic priming. No single molecule predicted conditioning status.

Protein Name	Mean Importance	Median Importance	Minimum Importance	Maximum Importance	Normalized Hits	Decision
Hemoglobin A1c	0	0	0	0	0	Rejected
AKAP9	0.715764	0	0	2.965617	0.070707	Rejected
DSPG3	1.024284	0	0	3.41555	0.252525	Rejected
TGF-beta RII	1.892373	2.415901	0	3.700416	0.525253	Tentative
ADH1B	2.334749	2.591644	0	3.949558	0.616162	Tentative
Aconitase 1	2.413065	2.644429	0	3.715384	0.606061	Tentative
Arp2	2.182399	2.443023	0	4.34588	0.545455	Tentative
CA2	2.358065	2.615793	0	3.701565	0.59596	Tentative
Dermcidin	2.262532	2.636758	0	4.076119	0.59596	Tentative
APA	2.358509	2.591644	0	3.836425	0.606061	Tentative
CRTAC1	2.107907	2.452617	0	4.28286	0.545455	Tentative
Glutathione Synthetase/GSS	2.4452	2.610077	0	4.206068	0.626263	Tentative
Apo (a)	2.357769	2.611468	0	3.813317	0.626263	Tentative
Tie-2	2.262043	2.610077	0	4.091755	0.555556	Tentative
Histone H3.3	2.371055	2.611468	0	4.439651	0.606061	Tentative
GRHPR	2.246976	2.590461	0	3.852036	0.585859	Tentative
GLUD1	2.007487	2.398292	0	3.965258	0.474747	Tentative
Glypican 5	2.357302	2.615793	0	3.836425	0.656566	Tentative
ARPC3	2.27314	2.562445	0	3.817966	0.606061	Tentative
BRSK1	2.414462	2.615793	0	3.705485	0.656566	Tentative
