# Supplementary material for: Decoded cardiopoietic cell secretome linkage to heart repair biosignature
Source: Stem Cells Transl Med. 2024 Sep 11;13(11):1144–59. doi: 10.1093/stcltm/szae067 (PMC11555478; doi:10.1093/stcltm/szae067)
Supplement: szae067_suppl_Supplementary_Figures [file szae067_suppl_supplementary_figures.pdf]

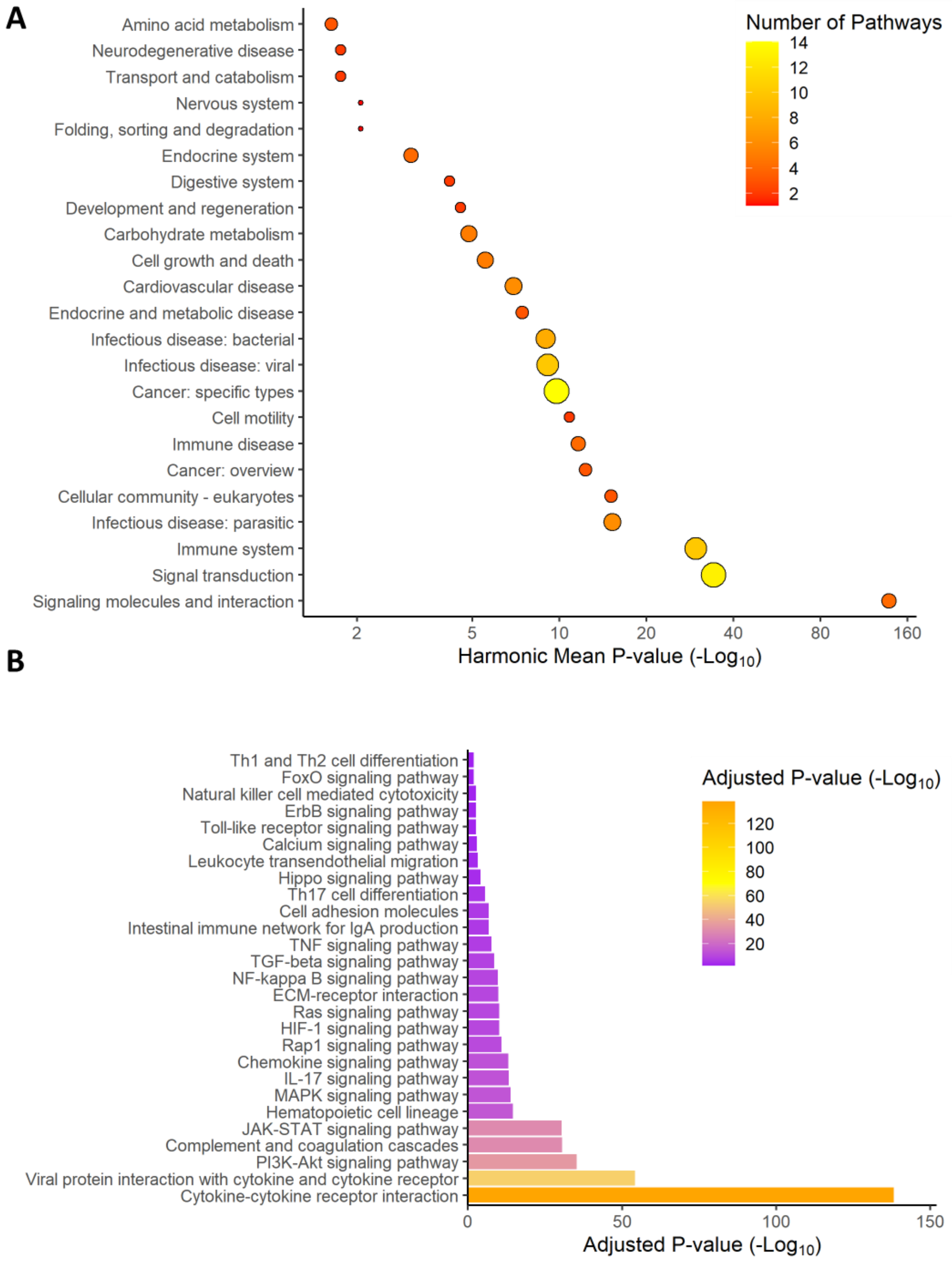

Supplementary Figure 1. KEGG pathways enriched in cardiopoietic secretome. (A) Cardiopoiesis promotes overrepresentation of 23 Biological Relationships of Information Transmitting Entities (BRITE) KEGG pathway classes including immunomodulatory, signaling, and cardiovascular-related KEGG pathways. Bubble color and size signify the number of KEGG pathways contained within each class. Signaling-related classes are prioritized by degree of enrichment and number of contained pathways. **(B)** Enriched KEGG signaling pathways include immunomodulatory and growth factor pathways. Color represents the p-value.

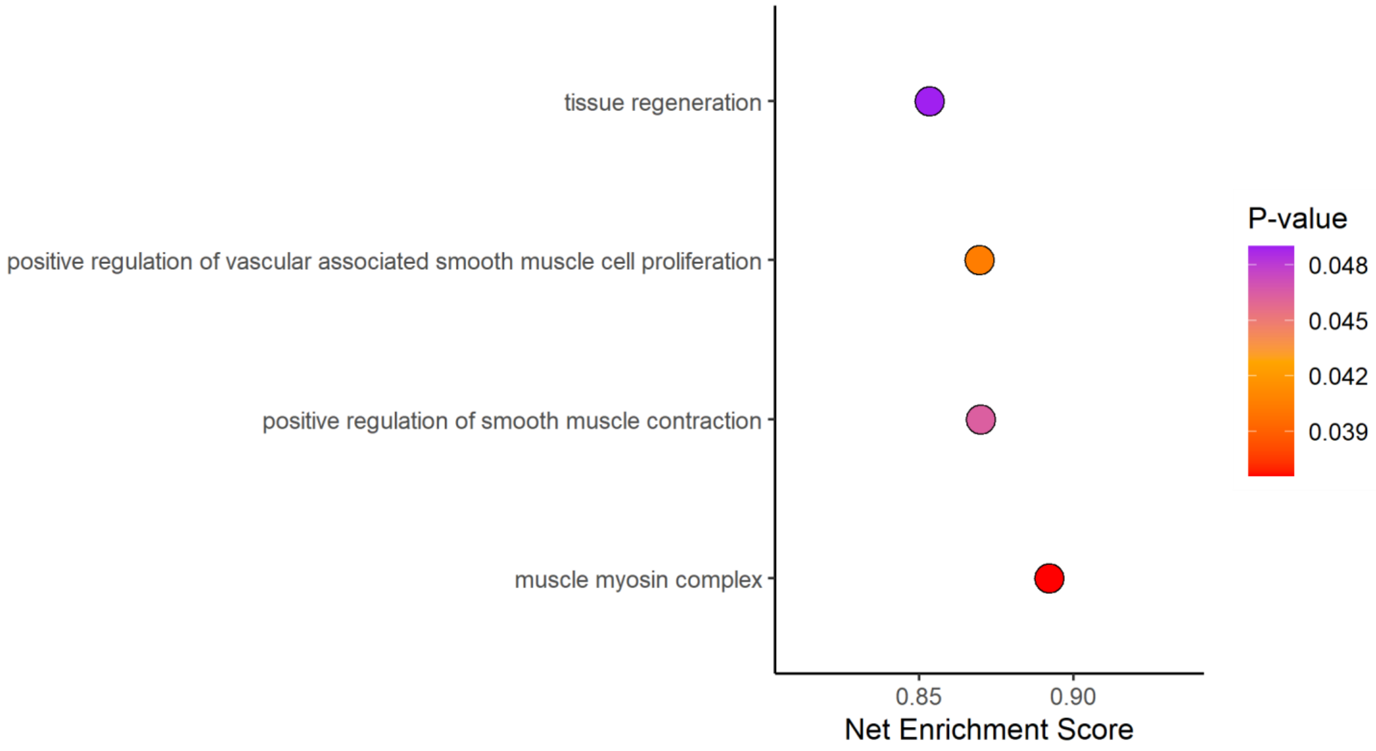

Supplementary Figure 2. Upregulated cardioregenerative Gene Ontology annotations in the myocardial proteome of cardiopoietic cell-treated infarcted hearts.

Gene set enrichment analysis of the differentially expressed myocardial proteome following cardiopoietic cell-treatment of infarcted hearts independently revealed a cardioregenerative signature featuring upregulated cardiomyogenic annotations.

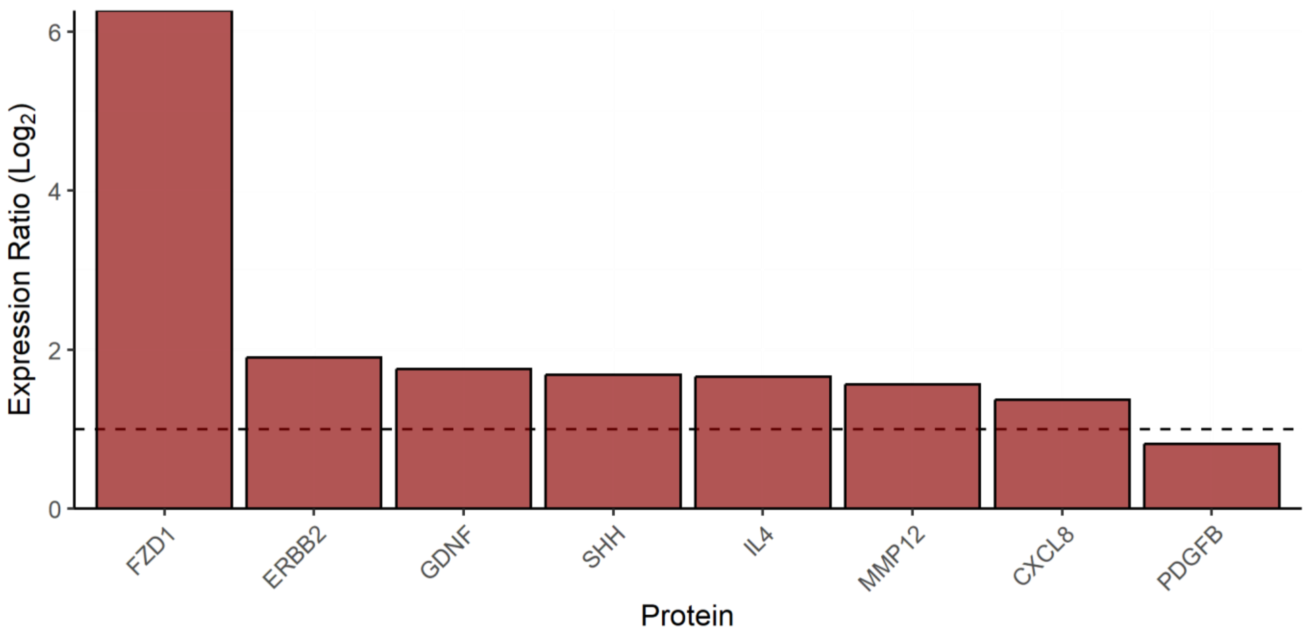

Supplementary Figure 3. Upregulated candidate paracrine effectors validated in secretomes of cells with therapeutic proficiency.

The expression ratio (Log_2) of 8 probed candidate paracrine effectors in the secretome of human cardiopoietic cells with/without therapeutic proficiency. The dotted line represents a 2-fold expression ratio.
