# Supplementary material for: Decoded cardiopoietic cell secretome linkage to heart repair biosignature
Source: Stem Cells Transl Med. 2024 Sep 11;13(11):1144–59. doi: 10.1093/stcltm/szae067 (PMC11555478; doi:10.1093/stcltm/szae067)
Supplement: szae067_suppl_Supplementary_Table_S2 [file szae067_suppl_supplementary_table_s2.pdf]

**Supplemental Table 2. Processes Enriched in Cardiopoietic Secretome Network.** Interactome overrepresented biological processes spanned 12 macroclusters comprising 19 subclusters.

| Macrocluster | Subcluster | GO Biological Process | adjustedPval | pValue | N | nn | x | xx |
|---|---|---|---|---|---|---|---|---|
| Cardioangiomyogenesis | Heart development | heart development | 8.82E-09 | 5.23E-10 | 14223 | 221 | 1066 | 45 |
| Cardioangiomyogenesis | Heart development | cardiac muscle tissue development | 5.03E-04 | 7.24E-05 | 14223 | 58 | 1066 | 14 |
| Cardioangiomyogenesis | Heart development | cardiac ventricle development | 1.87E-03 | 3.21E-04 | 14223 | 31 | 1066 | 9 |
| Cardioangiomyogenesis | Heart development | cardiac chamber development | 2.51E-03 | 4.58E-04 | 14223 | 39 | 1066 | 10 |
| Cardioangiomyogenesis | Heart development | ventricular cardiac muscle tissue development | 4.20E-03 | 8.16E-04 | 14223 | 22 | 1066 | 7 |
| Cardioangiomyogenesis | Heart development | negative regulation of cardiac muscle cell apoptosis | 7.32E-03 | 1.59E-03 | 14223 | 4 | 1066 | 3 |
| Cardioangiomyogenesis | Heart development | regulation of cardiac muscle cell apoptosis | 7.32E-03 | 1.59E-03 | 14223 | 4 | 1066 | 3 |
| Cardioangiomyogenesis | Heart morphogenesis | heart morphogenesis | 3.52E-03 | 6.63E-04 | 14223 | 87 | 1066 | 16 |
| Cardioangiomyogenesis | Heart morphogenesis | cardiac chamber morphogenesis | 3.62E-03 | 6.82E-04 | 14223 | 34 | 1066 | 9 |
| Cardioangiomyogenesis | Heart morphogenesis | ventricular cardiac muscle tissue morphogenesis | 4.20E-03 | 8.16E-04 | 14223 | 22 | 1066 | 7 |
| Cardioangiomyogenesis | Heart morphogenesis | positive regulation of cardiac muscle hypertrophy | 7.32E-03 | 1.59E-03 | 14223 | 4 | 1066 | 3 |
| Cardioangiomyogenesis | Heart morphogenesis | positive regulation of muscle hypertrophy | 7.32E-03 | 1.59E-03 | 14223 | 4 | 1066 | 3 |
| Cardioangiomyogenesis | Heart morphogenesis | cardiac ventricle morphogenesis | 8.39E-03 | 1.89E-03 | 14223 | 25 | 1066 | 7 |
| Cardioangiomyogenesis | Heart morphogenesis | cardiac muscle tissue morphogenesis | 1.27E-02 | 3.05E-03 | 14223 | 27 | 1066 | 7 |
| Cardioangiomyogenesis | Heart morphogenesis | regulation of cardiac muscle hypertrophy | 7.02E-02 | 2.50E-02 | 14223 | 9 | 1066 | 3 |
| Cardioangiomyogenesis | Muscle development | skeletal system development | 1.45E-14 | 4.51E-16 | 14223 | 334 | 1066 | 71 |
| Cardioangiomyogenesis | Muscle development | muscle structure development | 5.48E-13 | 2.04E-14 | 14223 | 266 | 1066 | 59 |
| Cardioangiomyogenesis | Muscle development | muscle organ development | 2.27E-08 | 1.47E-09 | 14223 | 220 | 1066 | 44 |
| Cardioangiomyogenesis | Muscle development | muscle cell differentiation | 2.27E-08 | 1.46E-09 | 14223 | 117 | 1066 | 30 |
| Cardioangiomyogenesis | Muscle development | muscle tissue development | 3.67E-07 | 3.01E-08 | 14223 | 132 | 1066 | 30 |
| Cardioangiomyogenesis | Muscle development | regulation of striated muscle tissue development | 9.84E-07 | 8.41E-08 | 14223 | 51 | 1066 | 17 |
| Cardioangiomyogenesis | Muscle development | striated muscle tissue development | 3.01E-05 | 3.21E-06 | 14223 | 122 | 1066 | 25 |
| Cardioangiomyogenesis | Muscle development | regulation of skeletal muscle tissue development | 7.93E-05 | 9.38E-06 | 14223 | 26 | 1066 | 10 |
| Cardioangiomyogenesis | Muscle development | regulation of striated muscle cell differentiation | 1.58E-04 | 2.02E-05 | 14223 | 28 | 1066 | 10 |
| Cardioangiomyogenesis | Muscle development | regulation of muscle cell differentiation | 7.97E-04 | 1.21E-04 | 14223 | 40 | 1066 | 11 |
| Cardioangiomyogenesis | Muscle development | muscle fiber development | 1.37E-03 | 2.25E-04 | 14223 | 36 | 1066 | 10 |
| Cardioangiomyogenesis | Muscle development | somatic muscle development | 2.34E-03 | 4.20E-04 | 14223 | 3 | 1066 | 3 |
| Cardioangiomyogenesis | Muscle development | muscle cell development | 3.94E-03 | 7.54E-04 | 14223 | 56 | 1066 | 12 |
| Cardioangiomyogenesis | Muscle development | skeletal muscle fiber development | 4.04E-03 | 7.77E-04 | 14223 | 28 | 1066 | 8 |
| Cardioangiomyogenesis | Muscle development | striated muscle cell development | 4.21E-03 | 8.21E-04 | 14223 | 49 | 1066 | 11 |
| Cardioangiomyogenesis | Muscle development | positive regulation of muscle cell differentiation | 5.22E-03 | 1.05E-03 | 14223 | 17 | 1066 | 6 |
| Cardioangiomyogenesis | Muscle development | regulation of muscle system process | 6.33E-03 | 1.32E-03 | 14223 | 84 | 1066 | 15 |
| Cardioangiomyogenesis | Muscle development | skeletal muscle organ development | 6.81E-03 | 1.43E-03 | 14223 | 68 | 1066 | 13 |
| Cardioangiomyogenesis | Muscle development | muscle system process | 7.61E-03 | 1.66E-03 | 14223 | 168 | 1066 | 24 |
| Cardioangiomyogenesis | Muscle development | muscle tissue morphogenesis | 1.27E-02 | 3.05E-03 | 14223 | 27 | 1066 | 7 |
| Cardioangiomyogenesis | Muscle development | skeletal muscle tissue development | 1.38E-02 | 3.33E-03 | 14223 | 66 | 1066 | 12 |
| Cardioangiomyogenesis | Muscle development | regulation of muscle hypertrophy | 1.79E-02 | 4.57E-03 | 14223 | 10 | 1066 | 4 |
| Cardioangiomyogenesis | Vessel development/angiogenesis | blood vessel development | 2.64E-18 | 6.26E-20 | 14223 | 266 | 1066 | 68 |
| Cardioangiomyogenesis | Vessel development/angiogenesis | vasculature development | 1.44E-17 | 3.55E-19 | 14223 | 274 | 1066 | 68 |
| Cardioangiomyogenesis | Vessel development/angiogenesis | angiogenesis | 7.07E-14 | 2.41E-15 | 14223 | 153 | 1066 | 44 |
| Cardioangiomyogenesis | Vessel development/angiogenesis | regulation of angiogenesis | 2.68E-12 | 1.06E-13 | 14223 | 89 | 1066 | 31 |
| Cardioangiomyogenesis | Vessel development/angiogenesis | positive regulation of angiogenesis | 1.27E-08 | 7.65E-10 | 14223 | 44 | 1066 | 18 |
| Cardioangiomyogenesis | Vessel development/angiogenesis | negative regulation of angiogenesis | 3.91E-04 | 5.54E-05 | 14223 | 31 | 1066 | 10 |
| Cardioangiomyogenesis | Vessel development/angiogenesis | vasculogenesis | 6.13E-03 | 1.27E-03 | 14223 | 44 | 1066 | 10 |
| Cardioangiomyogenesis | Vessel development/angiogenesis | artery development | 7.02E-03 | 1.48E-03 | 14223 | 18 | 1066 | 6 |
| Cardioangiomyogenesis | Vessel morphogenesis/migration | blood vessel morphogenesis | 2.74E-16 | 7.54E-18 | 14223 | 220 | 1066 | 58 |
| Cardioangiomyogenesis | Vessel morphogenesis/migration | patterning of blood vessels | 3.11E-05 | 3.35E-06 | 14223 | 19 | 1066 | 9 |
| Cardioangiomyogenesis | Vessel morphogenesis/migration | positive regulation of blood vessel endothelial cell migration | 1.17E-03 | 1.89E-04 | 14223 | 13 | 1066 | 6 |

| | | | | | | | | |
|---|---|---|---|---|---|---|---|---|
| Cardioangiomyogenesis | Vessel morphogenesis/migration | regulation of blood vessel endothelial cell migration | 4.20E-03 | 8.16E-04 | 14223 | 22 | 1066 | 7 |
| Cardioangiomyogenesis | Vessel morphogenesis/migration | blood vessel endothelial cell migration | 5.77E-03 | 1.19E-03 | 14223 | 12 | 1066 | 5 |
| Cell Function | Cell Function | response to chemical stimulus | 3.14E-58 | 2.64E-61 | 14223 | 1473 | 1066 | 293 |
| Cell Function | Cell Function | response to stress | 6.64E-54 | 8.37E-57 | 14223 | 1780 | 1066 | 320 |
| Cell Function | Cell Function | positive regulation of cellular process | 1.68E-52 | 2.47E-55 | 14223 | 2009 | 1066 | 342 |
| Cell Function | Cell Function | response to stimulus | 2.05E-49 | 6.02E-52 | 14223 | 3634 | 1066 | 492 |
| Cell Function | Cell Function | signaling | 2.05E-49 | 5.66E-52 | 14223 | 3129 | 1066 | 446 |
| Cell Function | Cell Function | response to external stimulus | 8.51E-39 | 4.47E-41 | 14223 | 555 | 1066 | 142 |
| Cell Function | Cell Function | negative regulation of biological process | 1.98E-34 | 1.29E-36 | 14223 | 2027 | 1066 | 304 |
| Cell Function | Cell Function | response to organic substance | 4.92E-33 | 3.61E-35 | 14223 | 878 | 1066 | 175 |
| Cell Function | Cell Function | positive regulation of cell communication | 1.67E-30 | 1.37E-32 | 14223 | 415 | 1066 | 109 |
| Cell Function | Cell Function | negative regulation of cellular process | 2.61E-28 | 2.41E-30 | 14223 | 1850 | 1066 | 271 |
| Cell Function | Cell Function | signaling process | 2.35E-26 | 2.76E-28 | 14223 | 2159 | 1066 | 296 |
| Cell Function | Cell Function | regulation of response to external stimulus | 3.56E-25 | 4.86E-27 | 14223 | 194 | 1066 | 66 |
| Cell Function | Cell Function | regulation of cell communication | 1.66E-24 | 2.38E-26 | 14223 | 1156 | 1066 | 189 |
| Cell Function | Cell Function | regulation of cellular process | 2.61E-24 | 3.89E-26 | 14223 | 6205 | 1066 | 630 |
| Cell Function | Cell Function | response to endogenous stimulus | 2.17E-23 | 3.82E-25 | 14223 | 500 | 1066 | 109 |
| Cell Function | Cell Function | positive regulation of response to stimulus | 8.89E-22 | 1.81E-23 | 14223 | 271 | 1066 | 74 |
| Cell Function | Cell Function | response to hormone stimulus | 1.08E-20 | 2.31E-22 | 14223 | 446 | 1066 | 97 |
| Cell Function | Cell Function | cell activation | 3.27E-20 | 7.34E-22 | 14223 | 280 | 1066 | 73 |
| Cell Function | Cell Function | positive regulation of biosynthetic process | 3.91E-18 | 9.44E-20 | 14223 | 734 | 1066 | 127 |
| Cell Function | Cell Function | regulation of cell activation | 9.10E-18 | 2.22E-19 | 14223 | 193 | 1066 | 56 |
| Cell Function | Cell Function | cellular response to chemical stimulus | 1.26E-16 | 3.40E-18 | 14223 | 404 | 1066 | 84 |
| Cell Function | Cell Function | regulation of response to stress | 2.16E-16 | 5.86E-18 | 14223 | 324 | 1066 | 73 |
| Cell Function | Cell Function | cellular process | 2.17E-16 | 5.93E-18 | 14223 | 9323 | 1066 | 823 |
| Cell Function | Cell Function | response to biotic stimulus | 1.25E-15 | 3.51E-17 | 14223 | 451 | 1066 | 88 |
| Cell Function | Cell Function | positive regulation of response to external stimulus | 5.97E-15 | 1.76E-16 | 14223 | 88 | 1066 | 34 |
| Cell Function | Cell Function | cell-cell signaling | 1.46E-14 | 4.56E-16 | 14223 | 588 | 1066 | 102 |
| Cell Function | Cell Function | positive regulation of transport | 1.58E-14 | 5.02E-16 | 14223 | 275 | 1066 | 63 |
| Cell Function | Cell Function | response to oxygen levels | 1.76E-14 | 5.61E-16 | 14223 | 160 | 1066 | 46 |
| Cell Function | Cell Function | cell communication | 1.82E-14 | 5.83E-16 | 14223 | 815 | 1066 | 127 |
| Cell Function | Cell Function | response to hypoxia | 4.22E-14 | 1.41E-15 | 14223 | 151 | 1066 | 44 |
| Cell Function | Cell Function | positive regulation of protein transport | 4.14E-13 | 1.51E-14 | 14223 | 89 | 1066 | 32 |
| Cell Function | Cell Function | regulation of cellular component size | 3.56E-12 | 1.43E-13 | 14223 | 285 | 1066 | 60 |
| Cell Function | Cell Function | regulation of cellular localization | 4.64E-12 | 1.89E-13 | 14223 | 310 | 1066 | 63 |
| Cell Function | Cell Function | positive regulation of DNA replication | 5.46E-12 | 2.25E-13 | 14223 | 34 | 1066 | 19 |
| Cell Function | Cell Function | regulation of transport | 5.65E-12 | 2.35E-13 | 14223 | 520 | 1066 | 88 |
| Cell Function | Cell Function | positive regulation of gene expression | 1.67E-11 | 7.21E-13 | 14223 | 602 | 1066 | 96 |
| Cell Function | Cell Function | regulation of cellular component organization | 4.59E-11 | 2.07E-12 | 14223 | 540 | 1066 | 88 |
| Cell Function | Cell Function | positive regulation of cytokine production | 1.05E-10 | 4.92E-12 | 14223 | 107 | 1066 | 32 |
| Cell Function | Cell Function | regulation of gene-specific transcription | 2.39E-10 | 1.15E-11 | 14223 | 213 | 1066 | 47 |
| Cell Function | Cell Function | positive regulation of cell activation | 2.55E-10 | 1.24E-11 | 14223 | 123 | 1066 | 34 |
| Cell Function | Cell Function | regulation of establishment of protein localization | 4.50E-10 | 2.23E-11 | 14223 | 159 | 1066 | 39 |
| Cell Function | Cell Function | cellular response to stimulus | 4.51E-10 | 2.25E-11 | 14223 | 990 | 1066 | 132 |
| Cell Function | Cell Function | negative regulation of cell activation | 1.04E-09 | 5.38E-11 | 14223 | 63 | 1066 | 23 |
| Cell Function | Cell Function | regulation of protein localization | 1.12E-09 | 5.81E-11 | 14223 | 178 | 1066 | 41 |
| Cell Function | Cell Function | regulation of kinase activity | 1.17E-09 | 6.13E-11 | 14223 | 386 | 1066 | 67 |
| Cell Function | Cell Function | regulation of protein transport | 1.17E-09 | 6.11E-11 | 14223 | 150 | 1066 | 37 |
| Cell Function | Cell Function | cellular component organization | 1.27E-09 | 6.71E-11 | 14223 | 2558 | 1066 | 273 |
| Cell Function | Cell Function | regulation of DNA replication | 1.46E-09 | 7.78E-11 | 14223 | 75 | 1066 | 25 |

| | | | | | | | | |
|---|---|---|---|---|---|---|---|---|
| Cell Function | Cell Function | response to peptide hormone stimulus | 2.08E-09 | 1.14E-10 | 14223 | 189 | 1066 | 42 |
| Cell Function | Cell Function | extracellular structure organization | 2.47E-09 | 1.37E-10 | 14223 | 161 | 1066 | 38 |
| Cell Function | Cell Function | positive regulation of transcription | 2.55E-09 | 1.42E-10 | 14223 | 574 | 1066 | 87 |
| Cell Function | Cell Function | protein processing | 8.82E-09 | 5.22E-10 | 14223 | 106 | 1066 | 29 |
| Cell Function | Cell Function | response to nutrient levels | 9.25E-09 | 5.50E-10 | 14223 | 245 | 1066 | 48 |
| Cell Function | Cell Function | cellular calcium ion homeostasis | 1.25E-08 | 7.53E-10 | 14223 | 185 | 1066 | 40 |
| Cell Function | Cell Function | positive regulation of cellular component organization | 1.35E-08 | 8.25E-10 | 14223 | 224 | 1066 | 45 |
| Cell Function | Cell Function | positive regulation of secretion | 2.01E-08 | 1.28E-09 | 14223 | 137 | 1066 | 33 |
| Cell Function | Cell Function | cellular metal ion homeostasis | 2.07E-08 | 1.32E-09 | 14223 | 196 | 1066 | 41 |
| Cell Function | Cell Function | calcium ion homeostasis | 3.55E-08 | 2.38E-09 | 14223 | 192 | 1066 | 40 |
| Cell Function | Cell Function | regulation of protein secretion | 4.06E-08 | 2.77E-09 | 14223 | 69 | 1066 | 22 |
| Cell Function | Cell Function | cellular di-, tri-valent inorganic cation homeostasis | 5.10E-08 | 3.53E-09 | 14223 | 226 | 1066 | 44 |
| Cell Function | Cell Function | positive regulation of transcription factor activity | 5.28E-08 | 3.68E-09 | 14223 | 82 | 1066 | 24 |
| Cell Function | Cell Function | positive regulation of transcription regulator activity | 5.28E-08 | 3.68E-09 | 14223 | 82 | 1066 | 24 |
| Cell Function | Cell Function | regulation of DNA binding | 5.30E-08 | 3.71E-09 | 14223 | 157 | 1066 | 35 |
| Cell Function | Cell Function | regulation of organelle organization | 5.65E-08 | 3.98E-09 | 14223 | 243 | 1066 | 46 |
| Cell Function | Cell Function | regulation of transcription factor activity | 5.93E-08 | 4.24E-09 | 14223 | 136 | 1066 | 32 |
| Cell Function | Cell Function | regulation of transcription regulator activity | 5.93E-08 | 4.24E-09 | 14223 | 136 | 1066 | 32 |
| Cell Function | Cell Function | regulation of cell size | 7.49E-08 | 5.43E-09 | 14223 | 221 | 1066 | 43 |
| Cell Function | Cell Function | chemical homeostasis | 7.80E-08 | 5.67E-09 | 14223 | 531 | 1066 | 78 |
| Cell Function | Cell Function | metal ion homeostasis | 8.49E-08 | 6.19E-09 | 14223 | 206 | 1066 | 41 |
| Cell Function | Cell Function | positive regulation of intracellular protein transport | 8.77E-08 | 6.46E-09 | 14223 | 44 | 1066 | 17 |
| Cell Function | Cell Function | positive regulation of transcription from RNA polymerase II promoter | 1.01E-07 | 7.51E-09 | 14223 | 386 | 1066 | 62 |
| Cell Function | Cell Function | response to abiotic stimulus | 1.10E-07 | 8.24E-09 | 14223 | 405 | 1066 | 64 |
| Cell Function | Cell Function | regulation of homeostatic process | 1.50E-07 | 1.14E-08 | 14223 | 134 | 1066 | 31 |
| Cell Function | Cell Function | cellular cation homeostasis | 1.69E-07 | 1.30E-08 | 14223 | 252 | 1066 | 46 |
| Cell Function | Cell Function | positive regulation of gene-specific transcription | 2.48E-07 | 1.98E-08 | 14223 | 137 | 1066 | 31 |
| Cell Function | Cell Function | positive regulation of intracellular transport | 2.59E-07 | 2.08E-08 | 14223 | 47 | 1066 | 17 |
| Cell Function | Cell Function | positive regulation of transcription, DNA-dependent | 2.63E-07 | 2.12E-08 | 14223 | 499 | 1066 | 73 |
| Cell Function | Cell Function | response to drug | 3.00E-07 | 2.44E-08 | 14223 | 257 | 1066 | 46 |
| Cell Function | Cell Function | di-, tri-valent inorganic cation homeostasis | 3.28E-07 | 2.68E-08 | 14223 | 241 | 1066 | 44 |
| Cell Function | Cell Function | cellular homeostasis | 3.90E-07 | 3.21E-08 | 14223 | 466 | 1066 | 69 |
| Cell Function | Cell Function | regulation of gene-specific transcription from RNA polymerase II promoter | 4.13E-07 | 3.42E-08 | 14223 | 155 | 1066 | 33 |
| Cell Function | Cell Function | positive regulation of protein secretion | 7.12E-07 | 6.02E-08 | 14223 | 50 | 1066 | 17 |
| Cell Function | Cell Function | positive regulation of cell size | 1.05E-06 | 9.04E-08 | 14223 | 57 | 1066 | 18 |
| Cell Function | Cell Function | response to nutrient | 1.07E-06 | 9.22E-08 | 14223 | 169 | 1066 | 34 |
| Cell Function | Cell Function | cellular ion homeostasis | 1.67E-06 | 1.47E-07 | 14223 | 371 | 1066 | 57 |
| Cell Function | Cell Function | response to inorganic substance | 2.13E-06 | 1.89E-07 | 14223 | 240 | 1066 | 42 |
| Cell Function | Cell Function | response to temperature stimulus | 2.22E-06 | 1.99E-07 | 14223 | 99 | 1066 | 24 |
| Cell Function | Cell Function | cellular chemical homeostasis | 2.82E-06 | 2.54E-07 | 14223 | 377 | 1066 | 57 |
| Cell Function | Cell Function | positive regulation of protein import into nucleus, translocation | 5.42E-06 | 5.10E-07 | 14223 | 16 | 1066 | 9 |
| Cell Function | Cell Function | cation homeostasis | 6.63E-06 | 6.32E-07 | 14223 | 286 | 1066 | 46 |
| Cell Function | Cell Function | regulation of hormone secretion | 6.91E-06 | 6.60E-07 | 14223 | 84 | 1066 | 21 |
| Cell Function | Cell Function | regulation of activin receptor signaling pathway | 9.04E-06 | 8.89E-07 | 14223 | 13 | 1066 | 8 |
| Cell Function | Cell Function | negative regulation of cell communication | 1.18E-05 | 1.19E-06 | 14223 | 329 | 1066 | 50 |
| Cell Function | Cell Function | ion homeostasis | 1.44E-05 | 1.46E-06 | 14223 | 407 | 1066 | 58 |
| Cell Function | Cell Function | regulation of actin filament-based process | 2.45E-05 | 2.56E-06 | 14223 | 98 | 1066 | 22 |
| Cell Function | Cell Function | response to heat | 3.56E-05 | 3.90E-06 | 14223 | 65 | 1066 | 17 |
| Cell Function | Cell Function | positive regulation of gene-specific transcription from RNA polymerase II promoter | 3.92E-05 | 4.34E-06 | 14223 | 101 | 1066 | 22 |
| Cell Function | Cell Function | regulation of protein import into nucleus | 4.40E-05 | 4.89E-06 | 14223 | 66 | 1066 | 17 |

| | | | | | | | | |
|---|---|---|---|---|---|---|---|---|
| Cell Function | Cell Function | response to oxidative stress | 6.68E-05 | 7.69E-06 | 14223 | 186 | 1066 | 32 |
| Cell Function | Cell Function | negative regulation of hormone secretion | 7.18E-05 | 8.32E-06 | 14223 | 31 | 1066 | 11 |
| Cell Function | Cell Function | elevation of cytosolic calcium ion concentration | 7.60E-05 | 8.85E-06 | 14223 | 113 | 1066 | 23 |
| Cell Function | Cell Function | regulation of protein import into nucleus, translocation | 7.90E-05 | 9.29E-06 | 14223 | 21 | 1066 | 9 |
| Cell Function | Cell Function | cellular response to endogenous stimulus | 9.33E-05 | 1.11E-05 | 14223 | 172 | 1066 | 30 |
| Cell Function | Cell Function | protein import into nucleus, translocation | 9.84E-05 | 1.18E-05 | 14223 | 32 | 1066 | 11 |
| Cell Function | Cell Function | platelet activation | 9.84E-05 | 1.18E-05 | 14223 | 32 | 1066 | 11 |
| Cell Function | Cell Function | cytosolic calcium ion homeostasis | 1.00E-04 | 1.21E-05 | 14223 | 123 | 1066 | 24 |
| Cell Function | Cell Function | positive regulation of DNA recombination | 1.00E-04 | 1.21E-05 | 14223 | 9 | 1066 | 6 |
| Cell Function | Cell Function | regulation of DNA recombination | 1.13E-04 | 1.39E-05 | 14223 | 27 | 1066 | 10 |
| Cell Function | Cell Function | negative regulation of gene-specific transcription | 1.25E-04 | 1.55E-05 | 14223 | 86 | 1066 | 19 |
| Cell Function | Cell Function | regulation of actin cytoskeleton organization | 1.35E-04 | 1.69E-05 | 14223 | 94 | 1066 | 20 |
| Cell Function | Cell Function | negative regulation of cellular component movement | 1.36E-04 | 1.72E-05 | 14223 | 72 | 1066 | 17 |
| Cell Function | Cell Function | regulation of transcription from RNA polymerase II promoter | 1.53E-04 | 1.94E-05 | 14223 | 745 | 1066 | 87 |
| Cell Function | Cell Function | regulation of intracellular protein transport | 1.57E-04 | 1.99E-05 | 14223 | 80 | 1066 | 18 |
| Cell Function | Cell Function | cellular response to organic substance | 1.90E-04 | 2.49E-05 | 14223 | 242 | 1066 | 37 |
| Cell Function | Cell Function | axis elongation | 2.13E-04 | 2.82E-05 | 14223 | 10 | 1066 | 6 |
| Cell Function | Cell Function | cellular response to hormone stimulus | 2.17E-04 | 2.88E-05 | 14223 | 163 | 1066 | 28 |
| Cell Function | Cell Function | positive regulation of glucose import | 2.51E-04 | 3.36E-05 | 14223 | 24 | 1066 | 9 |
| Cell Function | Cell Function | regulation of nucleocytoplasmic transport | 2.52E-04 | 3.37E-05 | 14223 | 83 | 1066 | 18 |
| Cell Function | Cell Function | intermediate filament cytoskeleton organization | 2.57E-04 | 3.47E-05 | 14223 | 19 | 1066 | 8 |
| Cell Function | Cell Function | response to radiation | 2.57E-04 | 3.46E-05 | 14223 | 218 | 1066 | 34 |
| Cell Function | Cell Function | cellular component assembly | 2.66E-04 | 3.60E-05 | 14223 | 911 | 1066 | 101 |
| Cell Function | Cell Function | cell morphogenesis | 3.17E-04 | 4.37E-05 | 14223 | 315 | 1066 | 44 |
| Cell Function | Cell Function | positive regulation of ubiquitin-protein ligase activity | 3.34E-04 | 4.63E-05 | 14223 | 70 | 1066 | 16 |
| Cell Function | Cell Function | cell projection organization | 3.37E-04 | 4.68E-05 | 14223 | 365 | 1066 | 49 |
| Cell Function | Cell Function | positive regulation of glucose transport | 3.52E-04 | 4.91E-05 | 14223 | 25 | 1066 | 9 |
| Cell Function | Cell Function | negative regulation of cell size | 4.69E-04 | 6.70E-05 | 14223 | 111 | 1066 | 21 |
| Cell Function | Cell Function | positive regulation of protein ubiquitination | 4.70E-04 | 6.73E-05 | 14223 | 95 | 1066 | 19 |
| Cell Function | Cell Function | negative regulation of gene-specific transcription from RNA polymerase II promoter | 4.91E-04 | 7.05E-05 | 14223 | 65 | 1066 | 15 |
| Cell Function | Cell Function | negative regulation of transport | 5.26E-04 | 7.61E-05 | 14223 | 163 | 1066 | 27 |
| Cell Function | Cell Function | regulation of cell projection organization | 5.52E-04 | 8.06E-05 | 14223 | 129 | 1066 | 23 |
| Cell Function | Cell Function | intermediate filament-based process | 5.54E-04 | 8.16E-05 | 14223 | 21 | 1066 | 8 |
| Cell Function | Cell Function | regulation of ubiquitin-protein ligase activity | 5.79E-04 | 8.56E-05 | 14223 | 81 | 1066 | 17 |
| Cell Function | Cell Function | response to fluid shear stress | 7.26E-04 | 1.09E-04 | 14223 | 12 | 1066 | 6 |
| Cell Function | Cell Function | negative regulation of homeostatic process | 7.91E-04 | 1.20E-04 | 14223 | 22 | 1066 | 8 |
| Cell Function | Cell Function | glucose transport | 8.74E-04 | 1.35E-04 | 14223 | 28 | 1066 | 9 |
| Cell Function | Cell Function | astrocyte activation | 9.46E-04 | 1.48E-04 | 14223 | 5 | 1066 | 4 |
| Cell Function | Cell Function | regulation of glucose transport | 1.09E-03 | 1.74E-04 | 14223 | 35 | 1066 | 10 |
| Cell Function | Cell Function | monosaccharide transport | 1.15E-03 | 1.83E-04 | 14223 | 29 | 1066 | 9 |
| Cell Function | Cell Function | reproductive cellular process | 1.18E-03 | 1.92E-04 | 14223 | 172 | 1066 | 27 |
| Cell Function | Cell Function | negative regulation of response to external stimulus | 1.44E-03 | 2.40E-04 | 14223 | 57 | 1066 | 13 |
| Cell Function | Cell Function | negative regulation of ubiquitin-protein ligase activity | 1.84E-03 | 3.15E-04 | 14223 | 66 | 1066 | 14 |
| Cell Function | Cell Function | cellular component biogenesis | 1.84E-03 | 3.15E-04 | 14223 | 1033 | 1066 | 107 |
| Cell Function | Cell Function | regulation of cellular response to stress | 2.01E-03 | 3.47E-04 | 14223 | 133 | 1066 | 22 |
| Cell Function | Cell Function | posttranscriptional regulation of gene expression | 2.03E-03 | 3.52E-04 | 14223 | 245 | 1066 | 34 |
| Cell Function | Cell Function | protein import into nucleus | 2.19E-03 | 3.83E-04 | 14223 | 75 | 1066 | 15 |
| Cell Function | Cell Function | response to UV-A | 2.34E-03 | 4.16E-04 | 14223 | 6 | 1066 | 4 |
| Cell Function | Cell Function | negative regulation of phagocytosis | 2.34E-03 | 4.20E-04 | 14223 | 3 | 1066 | 3 |
| Cell Function | Cell Function | regulation of glucose import | 2.34E-03 | 4.18E-04 | 14223 | 32 | 1066 | 9 |

| | | | | | | | | |
|---|---|---|---|---|---|---|---|---|
| Cell Function | Cell Function | intermediate filament organization | 2.38E-03 | 4.29E-04 | 14223 | 10 | 1066 | 5 |
| Cell Function | Cell Function | regulation of hormone levels | 2.38E-03 | 4.31E-04 | 14223 | 153 | 1066 | 24 |
| Cell Function | Cell Function | positive regulation of response to biotic stimulus | 2.38E-03 | 4.29E-04 | 14223 | 10 | 1066 | 5 |
| Cell Function | Cell Function | regulation of carbohydrate biosynthetic process | 2.45E-03 | 4.47E-04 | 14223 | 26 | 1066 | 8 |
| Cell Function | Cell Function | protein localization in nucleus | 3.02E-03 | 5.57E-04 | 14223 | 94 | 1066 | 17 |
| Cell Function | Cell Function | regulation of lipid transport | 3.08E-03 | 5.70E-04 | 14223 | 40 | 1066 | 10 |
| Cell Function | Cell Function | nuclear import | 3.18E-03 | 5.93E-04 | 14223 | 78 | 1066 | 15 |
| Cell Function | Cell Function | regulation of organic acid transport | 3.18E-03 | 5.94E-04 | 14223 | 21 | 1066 | 7 |
| Cell Function | Cell Function | positive regulation of proteasomal ubiquitin-dependent protein catabolic process | 3.18E-03 | 5.94E-04 | 14223 | 21 | 1066 | 7 |
| Cell Function | Cell Function | negative regulation of cell growth | 3.18E-03 | 5.96E-04 | 14223 | 103 | 1066 | 18 |
| Cell Function | Cell Function | negative regulation of protein ubiquitination | 3.18E-03 | 5.93E-04 | 14223 | 78 | 1066 | 15 |
| Cell Function | Cell Function | regulation of protein ubiquitination | 3.41E-03 | 6.40E-04 | 14223 | 121 | 1066 | 20 |
| Cell Function | Cell Function | regulation of cellular component biogenesis | 3.68E-03 | 6.94E-04 | 14223 | 158 | 1066 | 24 |
| Cell Function | Cell Function | negative regulation of cellular component organization | 3.78E-03 | 7.14E-04 | 14223 | 177 | 1066 | 26 |
| Cell Function | Cell Function | regulation of proteasomal protein catabolic process | 4.04E-03 | 7.77E-04 | 14223 | 28 | 1066 | 8 |
| Cell Function | Cell Function | positive regulation of steroid biosynthetic process | 4.62E-03 | 9.14E-04 | 14223 | 7 | 1066 | 4 |
| Cell Function | Cell Function | regulation of cytoskeleton organization | 4.83E-03 | 9.58E-04 | 14223 | 143 | 1066 | 22 |
| Cell Function | Cell Function | somatic cell DNA recombination | 5.37E-03 | 1.10E-03 | 14223 | 23 | 1066 | 7 |
| Cell Function | Cell Function | positive regulation of protein import into nucleus | 5.37E-03 | 1.10E-03 | 14223 | 23 | 1066 | 7 |
| Cell Function | Cell Function | positive regulation of survival gene product expression | 5.77E-03 | 1.19E-03 | 14223 | 12 | 1066 | 5 |
| Cell Function | Cell Function | positive regulation of cell projection organization | 6.00E-03 | 1.24E-03 | 14223 | 67 | 1066 | 13 |
| Cell Function | Cell Function | membrane invagination | 6.13E-03 | 1.27E-03 | 14223 | 223 | 1066 | 30 |
| Cell Function | Cell Function | endocytosis | 6.13E-03 | 1.27E-03 | 14223 | 223 | 1066 | 30 |
| Cell Function | Cell Function | positive regulation of nucleocytoplasmic transport | 6.17E-03 | 1.28E-03 | 14223 | 30 | 1066 | 8 |
| Cell Function | Cell Function | response to ozone | 7.32E-03 | 1.59E-03 | 14223 | 4 | 1066 | 3 |
| Cell Function | Cell Function | cellular response to reactive oxygen species | 7.43E-03 | 1.62E-03 | 14223 | 38 | 1066 | 9 |
| Cell Function | Cell Function | cellular membrane organization | 8.15E-03 | 1.83E-03 | 14223 | 384 | 1066 | 45 |
| Cell Function | Cell Function | cellular response to oxidative stress | 8.48E-03 | 1.91E-03 | 14223 | 54 | 1066 | 11 |
| Cell Function | Cell Function | membrane organization | 8.52E-03 | 1.92E-03 | 14223 | 385 | 1066 | 45 |
| Cell Function | Cell Function | positive regulation of lipid transport | 8.94E-03 | 2.03E-03 | 14223 | 19 | 1066 | 6 |
| Cell Function | Cell Function | response to UV | 9.76E-03 | 2.22E-03 | 14223 | 63 | 1066 | 12 |
| Cell Function | Cell Function | cell projection morphogenesis | 1.16E-02 | 2.69E-03 | 14223 | 234 | 1066 | 30 |
| Cell Function | Cell Function | regulation of phagocytosis | 1.17E-02 | 2.71E-03 | 14223 | 20 | 1066 | 6 |
| Cell Function | Cell Function | cytoskeleton organization | 1.24E-02 | 2.94E-03 | 14223 | 448 | 1066 | 50 |
| Cell Function | Cell Function | cellular response to biotic stimulus | 1.26E-02 | 2.99E-03 | 14223 | 49 | 1066 | 10 |
| Cell Function | Cell Function | cell part morphogenesis | 1.27E-02 | 3.01E-03 | 14223 | 246 | 1066 | 31 |
| Cell Function | Cell Function | regulation of response to biotic stimulus | 2.04E-02 | 5.29E-03 | 14223 | 37 | 1066 | 8 |
| Cell Function | Cell Function | positive regulation of organic acid transport | 2.41E-02 | 6.75E-03 | 14223 | 11 | 1066 | 4 |
| Cell Function | Cell Function | positive regulation of transcription factor import into nucleus | 3.04E-02 | 8.81E-03 | 14223 | 18 | 1066 | 5 |
| Cell Function | Cell Function | nucleocytoplasmic transport | 4.18E-02 | 1.28E-02 | 14223 | 146 | 1066 | 19 |
| Cell Function | Cell Function | nuclear transport | 4.39E-02 | 1.37E-02 | 14223 | 147 | 1066 | 19 |
| Cell Function | Cell Function | negative regulation of transcription from RNA polymerase II promoter | 4.82E-02 | 1.55E-02 | 14223 | 287 | 1066 | 32 |
| Cell Function | Cell Function | carbohydrate transport | 4.88E-02 | 1.62E-02 | 14223 | 62 | 1066 | 10 |
| Cell Function | Cell Function | negative regulation of transcription | 5.44E-02 | 1.86E-02 | 14223 | 495 | 1066 | 50 |
| Cell Function | Cell Function | protein localization in organelle | 5.54E-02 | 1.91E-02 | 14223 | 152 | 1066 | 19 |
| Cell Function | Cell Function | positive regulation of cytoskeleton organization | 5.73E-02 | 1.97E-02 | 14223 | 46 | 1066 | 8 |
| Cell Function | Cell Function | negative regulation of transcription, DNA-dependent | 6.42E-02 | 2.25E-02 | 14223 | 397 | 1066 | 41 |
| Cell Function | Cell Function | protein import | 8.04E-02 | 3.03E-02 | 14223 | 118 | 1066 | 15 |
| Cell Function | Cell Function | negative regulation of gene expression | 9.46E-02 | 3.67E-02 | 14223 | 552 | 1066 | 53 |
| Cell Function | Cell Function | response to light stimulus | 1.10E-01 | 4.36E-02 | 14223 | 145 | 1066 | 17 |

| | | | | | | | | |
|---|---|---|---|---|---|---|---|---|
| Cell Function | Cell Function | gene expression | 1.21E-01 | 5.05E-02 | 14223 | 1263 | 1066 | 110 |
| Cell Function | Cell Function | cellular response to stress | 1.36E-01 | 5.76E-02 | 14223 | 617 | 1066 | 57 |
| Cell Function | Cell Function | regulation of transcription factor import into nucleus | 1.48E-01 | 7.55E-02 | 14223 | 40 | 1066 | 6 |
| Cell Function | Cell Function | protein targeting | 1.61E-01 | 8.24E-02 | 14223 | 203 | 1066 | 21 |
| Cell Function | Cell Function | translation | 1.62E-01 | 8.32E-02 | 14223 | 296 | 1066 | 29 |
| Cell Function | Cell Function | regulation of endocytosis | 1.71E-01 | 8.83E-02 | 14223 | 72 | 1066 | 9 |
| Cell Function | Cell Function | cellular hormone metabolic process | 2.18E-01 | 1.19E-01 | 14223 | 66 | 1066 | 8 |
| Cell Function | Cell Function | negative regulation of endocytosis | 2.35E-01 | 1.30E-01 | 14223 | 17 | 1066 | 3 |
| Cell Function | Cell Function | DNA recombination | 2.69E-01 | 1.70E-01 | 14223 | 106 | 1066 | 11 |
| Cell Function | Cell Function | cellular protein localization | 2.92E-01 | 1.85E-01 | 14223 | 436 | 1066 | 38 |
| Cell Function | Cell Function | cellular macromolecule localization | 3.02E-01 | 1.97E-01 | 14223 | 439 | 1066 | 38 |
| Cell Function | Cell Function | cellular macromolecular complex assembly | 3.02E-01 | 2.07E-01 | 14223 | 317 | 1066 | 28 |
| Cell Function | Cell Function | regulation of purine nucleotide catabolic process | 3.67E-01 | 2.72E-01 | 14223 | 155 | 1066 | 14 |
| Cell Function | Cell Function | regulation of vesicle-mediated transport | 3.97E-01 | 2.97E-01 | 14223 | 121 | 1066 | 11 |
| Cell Function | Cell Function | organelle organization | 4.59E-01 | 3.73E-01 | 14223 | 1382 | 1066 | 107 |
| Cell Function | Cell Function | regulation of gene expression | 4.59E-01 | 3.73E-01 | 14223 | 2928 | 1066 | 224 |
| Cell Function | Cell Function | intracellular protein transport | 7.00E-01 | 6.42E-01 | 14223 | 366 | 1066 | 26 |
| Cell Function | Cell Function | vesicle-mediated transport | 7.19E-01 | 6.66E-01 | 14223 | 591 | 1066 | 42 |
| Cell Function | Cell Function | establishment of localization in cell | 8.73E-01 | 8.40E-01 | 14223 | 867 | 1066 | 58 |
| Cell Function | Cell Function | regulation of transcription | 8.84E-01 | 8.54E-01 | 14223 | 2619 | 1066 | 184 |
| Cell Function | Cell Function | protein localization | 8.98E-01 | 8.70E-01 | 14223 | 923 | 1066 | 61 |
| Cell Function | Cell Function | intracellular transport | 9.47E-01 | 9.27E-01 | 14223 | 667 | 1066 | 41 |
| Cell Function | Cell Function | protein transport | 9.98E-01 | 9.92E-01 | 14223 | 757 | 1066 | 41 |
| Cell Function | Cell Function | establishment of protein localization | 1.00E+00 | 9.94E-01 | 14223 | 768 | 1066 | 41 |
| Cell Function | Cell Function | establishment of localization | 1 | 1.00E+00 | 14223 | 2609 | 1066 | 149 |
| Cell Function | Cell Function | transport | 1 | 1.00E+00 | 14223 | 2575 | 1066 | 148 |
| Cell Function | Cell Function | biological_process | 1 | 1 | 14223 | 14223 | 1066 | 1066 |
| Cell Metabolism | Cell Metabolism | positive regulation of metabolic process | 7.44E-30 | 6.57E-32 | 14223 | 1023 | 1066 | 186 |
| Cell Metabolism | Cell Metabolism | positive regulation of cellular metabolic process | 1.59E-27 | 1.60E-29 | 14223 | 970 | 1066 | 175 |
| Cell Metabolism | Cell Metabolism | positive regulation of macromolecule metabolic process | 1.59E-27 | 1.57E-29 | 14223 | 944 | 1066 | 172 |
| Cell Metabolism | Cell Metabolism | positive regulation of phosphate metabolic process | 8.19E-27 | 9.12E-29 | 14223 | 137 | 1066 | 57 |
| Cell Metabolism | Cell Metabolism | positive regulation of phosphorus metabolic process | 8.19E-27 | 9.12E-29 | 14223 | 137 | 1066 | 57 |
| Cell Metabolism | Cell Metabolism | positive regulation of phosphorylation | 9.78E-26 | 1.23E-27 | 14223 | 133 | 1066 | 55 |
| Cell Metabolism | Cell Metabolism | regulation of cellular protein metabolic process | 2.94E-24 | 4.45E-26 | 14223 | 564 | 1066 | 119 |
| Cell Metabolism | Cell Metabolism | regulation of phosphorus metabolic process | 7.73E-24 | 1.25E-25 | 14223 | 547 | 1066 | 116 |
| Cell Metabolism | Cell Metabolism | regulation of phosphate metabolic process | 7.73E-24 | 1.25E-25 | 14223 | 547 | 1066 | 116 |
| Cell Metabolism | Cell Metabolism | regulation of protein amino acid phosphorylation | 2.03E-23 | 3.54E-25 | 14223 | 219 | 1066 | 68 |
| Cell Metabolism | Cell Metabolism | regulation of protein modification process | 2.24E-23 | 4.00E-25 | 14223 | 369 | 1066 | 91 |
| Cell Metabolism | Cell Metabolism | positive regulation of cellular protein metabolic process | 5.76E-23 | 1.05E-24 | 14223 | 292 | 1066 | 79 |
| Cell Metabolism | Cell Metabolism | regulation of phosphorylation | 6.52E-23 | 1.21E-24 | 14223 | 522 | 1066 | 111 |
| Cell Metabolism | Cell Metabolism | regulation of peptidyl-tyrosine phosphorylation | 8.06E-23 | 1.52E-24 | 14223 | 79 | 1066 | 40 |
| Cell Metabolism | Cell Metabolism | positive regulation of protein modification process | 1.38E-22 | 2.63E-24 | 14223 | 232 | 1066 | 69 |
| Cell Metabolism | Cell Metabolism | regulation of protein metabolic process | 1.89E-22 | 3.65E-24 | 14223 | 640 | 1066 | 125 |
| Cell Metabolism | Cell Metabolism | positive regulation of protein metabolic process | 6.03E-22 | 1.19E-23 | 14223 | 309 | 1066 | 80 |
| Cell Metabolism | Cell Metabolism | positive regulation of protein amino acid phosphorylation | 8.26E-22 | 1.65E-23 | 14223 | 120 | 1066 | 48 |
| Cell Metabolism | Cell Metabolism | positive regulation of macromolecule biosynthetic process | 1.70E-17 | 4.25E-19 | 14223 | 677 | 1066 | 119 |
| Cell Metabolism | Cell Metabolism | positive regulation of peptidyl-tyrosine phosphorylation | 1.77E-17 | 4.47E-19 | 14223 | 58 | 1066 | 30 |
| Cell Metabolism | Cell Metabolism | positive regulation of cellular biosynthetic process | 5.44E-17 | 1.40E-18 | 14223 | 722 | 1066 | 123 |
| Cell Metabolism | Cell Metabolism | positive regulation of catalytic activity | 1.22E-16 | 3.25E-18 | 14223 | 557 | 1066 | 103 |
| Cell Metabolism | Cell Metabolism | protein metabolic process | 1.45E-14 | 4.52E-16 | 14223 | 2611 | 1066 | 299 |

| | | | | | | | | |
|---|---|---|---|---|---|---|---|---|
| Cell Metabolism | Cell Metabolism | positive regulation of nitrogen compound metabolic process | 4.41E-14 | 1.48E-15 | 14223 | 678 | 1066 | 111 |
| Cell Metabolism | Cell Metabolism | regulation of catalytic activity | 2.18E-13 | 7.79E-15 | 14223 | 909 | 1066 | 134 |
| Cell Metabolism | Cell Metabolism | positive regulation of DNA metabolic process | 7.35E-13 | 2.75E-14 | 14223 | 65 | 1066 | 27 |
| Cell Metabolism | Cell Metabolism | multicellular organismal metabolic process | 1.66E-12 | 6.43E-14 | 14223 | 40 | 1066 | 21 |
| Cell Metabolism | Cell Metabolism | regulation of DNA metabolic process | 3.58E-12 | 1.44E-13 | 14223 | 131 | 1066 | 38 |
| Cell Metabolism | Cell Metabolism | positive regulation of nucleobase, nucleoside, nucleotide and nucleic acid metabolic process | 1.39E-11 | 5.94E-13 | 14223 | 655 | 1066 | 102 |
| Cell Metabolism | Cell Metabolism | macromolecule metabolic process | 2.26E-11 | 9.87E-13 | 14223 | 4009 | 1066 | 403 |
| Cell Metabolism | Cell Metabolism | positive regulation of transferase activity | 1.88E-10 | 8.98E-12 | 14223 | 257 | 1066 | 53 |
| Cell Metabolism | Cell Metabolism | collagen metabolic process | 9.32E-10 | 4.74E-11 | 14223 | 30 | 1066 | 16 |
| Cell Metabolism | Cell Metabolism | protein amino acid phosphorylation | 1.02E-09 | 5.23E-11 | 14223 | 657 | 1066 | 97 |
| Cell Metabolism | Cell Metabolism | protein maturation by peptide bond cleavage | 2.10E-09 | 1.16E-10 | 14223 | 82 | 1066 | 26 |
| Cell Metabolism | Cell Metabolism | regulation of transferase activity | 5.81E-09 | 3.35E-10 | 14223 | 401 | 1066 | 67 |
| Cell Metabolism | Cell Metabolism | primary metabolic process | 7.78E-09 | 4.54E-10 | 14223 | 5264 | 1066 | 489 |
| Cell Metabolism | Cell Metabolism | metabolic process | 1.31E-08 | 7.94E-10 | 14223 | 5930 | 1066 | 539 |
| Cell Metabolism | Cell Metabolism | negative regulation of response to stimulus | 1.66E-08 | 1.03E-09 | 14223 | 129 | 1066 | 32 |
| Cell Metabolism | Cell Metabolism | multicellular organismal catabolic process | 2.02E-08 | 1.29E-09 | 14223 | 27 | 1066 | 14 |
| Cell Metabolism | Cell Metabolism | regulation of cytokine biosynthetic process | 2.45E-08 | 1.60E-09 | 14223 | 79 | 1066 | 24 |
| Cell Metabolism | Cell Metabolism | negative regulation of metabolic process | 2.63E-08 | 1.72E-09 | 14223 | 870 | 1066 | 114 |
| Cell Metabolism | Cell Metabolism | negative regulation of cellular metabolic process | 3.16E-08 | 2.10E-09 | 14223 | 792 | 1066 | 106 |
| Cell Metabolism | Cell Metabolism | positive regulation of hydrolase activity | 1.26E-07 | 9.51E-09 | 14223 | 201 | 1066 | 40 |
| Cell Metabolism | Cell Metabolism | positive regulation of RNA metabolic process | 4.22E-07 | 3.50E-08 | 14223 | 505 | 1066 | 73 |
| Cell Metabolism | Cell Metabolism | phosphorylation | 5.19E-07 | 4.32E-08 | 14223 | 786 | 1066 | 101 |
| Cell Metabolism | Cell Metabolism | proteolysis | 6.79E-07 | 5.71E-08 | 14223 | 729 | 1066 | 95 |
| Cell Metabolism | Cell Metabolism | cellular protein metabolic process | 1.13E-06 | 9.74E-08 | 14223 | 2155 | 1066 | 223 |
| Cell Metabolism | Cell Metabolism | negative regulation of macromolecule metabolic process | 1.83E-06 | 1.61E-07 | 14223 | 807 | 1066 | 101 |
| Cell Metabolism | Cell Metabolism | positive regulation of nitric oxide biosynthetic process | 2.01E-06 | 1.79E-07 | 14223 | 27 | 1066 | 12 |
| Cell Metabolism | Cell Metabolism | negative regulation of protein metabolic process | 2.20E-06 | 1.97E-07 | 14223 | 215 | 1066 | 39 |
| Cell Metabolism | Cell Metabolism | regulation of nitric oxide biosynthetic process | 3.30E-06 | 3.04E-07 | 14223 | 33 | 1066 | 13 |
| Cell Metabolism | Cell Metabolism | negative regulation of cellular protein metabolic process | 3.39E-06 | 3.13E-07 | 14223 | 202 | 1066 | 37 |
| Cell Metabolism | Cell Metabolism | phosphate metabolic process | 4.39E-06 | 4.09E-07 | 14223 | 950 | 1066 | 113 |
| Cell Metabolism | Cell Metabolism | phosphorus metabolic process | 4.39E-06 | 4.09E-07 | 14223 | 950 | 1066 | 113 |
| Cell Metabolism | Cell Metabolism | response to organic cyclic substance | 8.20E-06 | 7.99E-07 | 14223 | 152 | 1066 | 30 |
| Cell Metabolism | Cell Metabolism | catabolic process | 9.37E-06 | 9.27E-07 | 14223 | 998 | 1066 | 116 |
| Cell Metabolism | Cell Metabolism | oxoacid metabolic process | 1.36E-05 | 1.38E-06 | 14223 | 564 | 1066 | 74 |
| Cell Metabolism | Cell Metabolism | carboxylic acid metabolic process | 1.36E-05 | 1.38E-06 | 14223 | 564 | 1066 | 74 |
| Cell Metabolism | Cell Metabolism | regulation of proteolysis | 1.65E-05 | 1.68E-06 | 14223 | 68 | 1066 | 18 |
| Cell Metabolism | Cell Metabolism | cellular ketone metabolic process | 1.73E-05 | 1.78E-06 | 14223 | 578 | 1066 | 75 |
| Cell Metabolism | Cell Metabolism | organic acid metabolic process | 2.13E-05 | 2.19E-06 | 14223 | 571 | 1066 | 74 |
| Cell Metabolism | Cell Metabolism | regulation of hydrolase activity | 2.93E-05 | 3.12E-06 | 14223 | 378 | 1066 | 54 |
| Cell Metabolism | Cell Metabolism | negative regulation of secretion | 3.08E-05 | 3.31E-06 | 14223 | 71 | 1066 | 18 |
| Cell Metabolism | Cell Metabolism | positive regulation of cytokine biosynthetic process | 3.52E-05 | 3.84E-06 | 14223 | 52 | 1066 | 15 |
| Cell Metabolism | Cell Metabolism | protein amino acid autophosphorylation | 6.51E-05 | 7.48E-06 | 14223 | 82 | 1066 | 19 |
| Cell Metabolism | Cell Metabolism | regulation of macromolecule metabolic process | 6.84E-05 | 7.88E-06 | 14223 | 3383 | 1066 | 313 |
| Cell Metabolism | Cell Metabolism | positive regulation of cellular catabolic process | 7.12E-05 | 8.23E-06 | 14223 | 55 | 1066 | 15 |
| Cell Metabolism | Cell Metabolism | glucose metabolic process | 9.58E-05 | 1.14E-05 | 14223 | 147 | 1066 | 27 |
| Cell Metabolism | Cell Metabolism | post-translational protein modification | 1.48E-04 | 1.87E-05 | 14223 | 1266 | 1066 | 134 |
| Cell Metabolism | Cell Metabolism | alcohol metabolic process | 1.52E-04 | 1.92E-05 | 14223 | 432 | 1066 | 57 |
| Cell Metabolism | Cell Metabolism | regulation of oxidoreductase activity | 1.69E-04 | 2.16E-05 | 14223 | 46 | 1066 | 13 |
| Cell Metabolism | Cell Metabolism | cellular catabolic process | 1.71E-04 | 2.19E-05 | 14223 | 769 | 1066 | 89 |
| Cell Metabolism | Cell Metabolism | peptidyl-tyrosine phosphorylation | 1.77E-04 | 2.30E-05 | 14223 | 40 | 1066 | 12 |

| | | | | | | | | |
|---|---|---|---|---|---|---|---|---|
| Cell Metabolism | Cell Metabolism | positive regulation of lipid metabolic process | 1.87E-04 | 2.44E-05 | 14223 | 53 | 1066 | 14 |
| Cell Metabolism | Cell Metabolism | regulation of metabolic process | 1.93E-04 | 2.53E-05 | 14223 | 3919 | 1066 | 352 |
| Cell Metabolism | Cell Metabolism | cellular metabolic process | 1.99E-04 | 2.61E-05 | 14223 | 4968 | 1066 | 434 |
| Cell Metabolism | Cell Metabolism | positive regulation of oxidoreductase activity | 2.16E-04 | 2.87E-05 | 14223 | 29 | 1066 | 10 |
| Cell Metabolism | Cell Metabolism | regulation of intracellular transport | 2.41E-04 | 3.22E-05 | 14223 | 98 | 1066 | 20 |
| Cell Metabolism | Cell Metabolism | response to activity | 3.52E-04 | 4.91E-05 | 14223 | 25 | 1066 | 9 |
| Cell Metabolism | Cell Metabolism | hexose metabolic process | 3.72E-04 | 5.21E-05 | 14223 | 186 | 1066 | 30 |
| Cell Metabolism | Cell Metabolism | regulation of monooxygenase activity | 3.91E-04 | 5.54E-05 | 14223 | 31 | 1066 | 10 |
| Cell Metabolism | Cell Metabolism | regulation of cellular carbohydrate metabolic process | 4.60E-04 | 6.57E-05 | 14223 | 44 | 1066 | 12 |
| Cell Metabolism | Cell Metabolism | regulation of glucose metabolic process | 5.04E-04 | 7.26E-05 | 14223 | 38 | 1066 | 11 |
| Cell Metabolism | Cell Metabolism | positive regulation of ligase activity | 5.45E-04 | 7.95E-05 | 14223 | 73 | 1066 | 16 |
| Cell Metabolism | Cell Metabolism | cytokine metabolic process | 5.54E-04 | 8.16E-05 | 14223 | 16 | 1066 | 7 |
| Cell Metabolism | Cell Metabolism | positive regulation of monooxygenase activity | 5.54E-04 | 8.16E-05 | 14223 | 16 | 1066 | 7 |
| Cell Metabolism | Cell Metabolism | regulation of carbohydrate metabolic process | 5.65E-04 | 8.35E-05 | 14223 | 45 | 1066 | 12 |
| Cell Metabolism | Cell Metabolism | negative regulation of biosynthetic process | 5.82E-04 | 8.62E-05 | 14223 | 624 | 1066 | 73 |
| Cell Metabolism | Cell Metabolism | negative regulation of phosphorus metabolic process | 5.95E-04 | 8.84E-05 | 14223 | 59 | 1066 | 14 |
| Cell Metabolism | Cell Metabolism | negative regulation of phosphate metabolic process | 5.95E-04 | 8.84E-05 | 14223 | 59 | 1066 | 14 |
| Cell Metabolism | Cell Metabolism | phagocytosis | 7.26E-04 | 1.09E-04 | 14223 | 53 | 1066 | 13 |
| Cell Metabolism | Cell Metabolism | regulation of fatty acid beta-oxidation | 7.26E-04 | 1.09E-04 | 14223 | 12 | 1066 | 6 |
| Cell Metabolism | Cell Metabolism | positive regulation of carbohydrate metabolic process | 7.91E-04 | 1.20E-04 | 14223 | 22 | 1066 | 8 |
| Cell Metabolism | Cell Metabolism | positive regulation of cellular carbohydrate metabolic process | 7.91E-04 | 1.20E-04 | 14223 | 22 | 1066 | 8 |
| Cell Metabolism | Cell Metabolism | regulation of lipid metabolic process | 8.50E-04 | 1.30E-04 | 14223 | 133 | 1066 | 23 |
| Cell Metabolism | Cell Metabolism | regulation of protein catabolic process | 8.59E-04 | 1.32E-04 | 14223 | 76 | 1066 | 16 |
| Cell Metabolism | Cell Metabolism | hexose transport | 8.74E-04 | 1.35E-04 | 14223 | 28 | 1066 | 9 |
| Cell Metabolism | Cell Metabolism | regulation of ligase activity | 8.88E-04 | 1.38E-04 | 14223 | 84 | 1066 | 17 |
| Cell Metabolism | Cell Metabolism | negative regulation of cellular biosynthetic process | 9.81E-04 | 1.54E-04 | 14223 | 614 | 1066 | 71 |
| Cell Metabolism | Cell Metabolism | small molecule catabolic process | 1.02E-03 | 1.61E-04 | 14223 | 264 | 1066 | 37 |
| Cell Metabolism | Cell Metabolism | regulation of cellular catabolic process | 1.04E-03 | 1.65E-04 | 14223 | 245 | 1066 | 35 |
| Cell Metabolism | Cell Metabolism | monocarboxylic acid metabolic process | 1.15E-03 | 1.84E-04 | 14223 | 305 | 1066 | 41 |
| Cell Metabolism | Cell Metabolism | serine family amino acid biosynthetic process | 1.17E-03 | 1.89E-04 | 14223 | 13 | 1066 | 6 |
| Cell Metabolism | Cell Metabolism | positive regulation of catabolic process | 1.24E-03 | 2.04E-04 | 14223 | 71 | 1066 | 15 |
| Cell Metabolism | Cell Metabolism | regulation of cellular metabolic process | 1.55E-03 | 2.60E-04 | 14223 | 3734 | 1066 | 329 |
| Cell Metabolism | Cell Metabolism | regulation of lipid biosynthetic process | 1.71E-03 | 2.88E-04 | 14223 | 58 | 1066 | 13 |
| Cell Metabolism | Cell Metabolism | positive regulation of glucose metabolic process | 1.74E-03 | 2.94E-04 | 14223 | 19 | 1066 | 7 |
| Cell Metabolism | Cell Metabolism | negative regulation of catalytic activity | 1.77E-03 | 3.00E-04 | 14223 | 302 | 1066 | 40 |
| Cell Metabolism | Cell Metabolism | negative regulation of macromolecule biosynthetic process | 1.82E-03 | 3.10E-04 | 14223 | 596 | 1066 | 68 |
| Cell Metabolism | Cell Metabolism | negative regulation of ligase activity | 1.84E-03 | 3.15E-04 | 14223 | 66 | 1066 | 14 |
| Cell Metabolism | Cell Metabolism | carbohydrate catabolic process | 2.00E-03 | 3.45E-04 | 14223 | 107 | 1066 | 19 |
| Cell Metabolism | Cell Metabolism | monosaccharide metabolic process | 2.02E-03 | 3.50E-04 | 14223 | 216 | 1066 | 31 |
| Cell Metabolism | Cell Metabolism | nicotinamide nucleotide metabolic process | 2.09E-03 | 3.65E-04 | 14223 | 38 | 1066 | 10 |
| Cell Metabolism | Cell Metabolism | negative regulation of phosphatase activity | 2.34E-03 | 4.16E-04 | 14223 | 6 | 1066 | 4 |
| Cell Metabolism | Cell Metabolism | positive regulation of small GTPase mediated signal transduction | 2.36E-03 | 4.24E-04 | 14223 | 20 | 1066 | 7 |
| Cell Metabolism | Cell Metabolism | cholesterol metabolic process | 2.38E-03 | 4.30E-04 | 14223 | 92 | 1066 | 17 |
| Cell Metabolism | Cell Metabolism | glucose catabolic process | 2.44E-03 | 4.43E-04 | 14223 | 53 | 1066 | 12 |
| Cell Metabolism | Cell Metabolism | amine metabolic process | 2.47E-03 | 4.51E-04 | 14223 | 411 | 1066 | 50 |
| Cell Metabolism | Cell Metabolism | macromolecule catabolic process | 2.89E-03 | 5.32E-04 | 14223 | 499 | 1066 | 58 |
| Cell Metabolism | Cell Metabolism | regulation of dephosphorylation | 2.91E-03 | 5.37E-04 | 14223 | 33 | 1066 | 9 |
| Cell Metabolism | Cell Metabolism | small molecule metabolic process | 3.10E-03 | 5.75E-04 | 14223 | 1366 | 1066 | 134 |
| Cell Metabolism | Cell Metabolism | response to reactive oxygen species | 3.13E-03 | 5.80E-04 | 14223 | 86 | 1066 | 16 |
| Cell Metabolism | Cell Metabolism | alcohol catabolic process | 3.18E-03 | 5.93E-04 | 14223 | 78 | 1066 | 15 |

| | | | | | | | | |
|---|---|---|---|---|---|---|---|---|
| Cell Metabolism | Cell Metabolism | regulation of catabolic process | 3.40E-03 | 6.38E-04 | 14223 | 283 | 1066 | 37 |
| Cell Metabolism | Cell Metabolism | pyridine nucleotide metabolic process | 3.73E-03 | 7.04E-04 | 14223 | 41 | 1066 | 10 |
| Cell Metabolism | Cell Metabolism | regulation of chemokine biosynthetic process | 3.86E-03 | 7.38E-04 | 14223 | 11 | 1066 | 5 |
| Cell Metabolism | Cell Metabolism | serine family amino acid metabolic process | 4.04E-03 | 7.77E-04 | 14223 | 28 | 1066 | 8 |
| Cell Metabolism | Cell Metabolism | regulation of proteasomal ubiquitin-dependent protein catabolic process | 4.04E-03 | 7.77E-04 | 14223 | 28 | 1066 | 8 |
| Cell Metabolism | Cell Metabolism | negative regulation of cytokine biosynthetic process | 4.20E-03 | 8.16E-04 | 14223 | 22 | 1066 | 7 |
| Cell Metabolism | Cell Metabolism | glyoxylate metabolic process | 4.62E-03 | 9.14E-04 | 14223 | 7 | 1066 | 4 |
| Cell Metabolism | Cell Metabolism | C21-steroid hormone biosynthetic process | 4.62E-03 | 9.14E-04 | 14223 | 7 | 1066 | 4 |
| Cell Metabolism | Cell Metabolism | response to hydrogen peroxide | 4.65E-03 | 9.20E-04 | 14223 | 65 | 1066 | 13 |
| Cell Metabolism | Cell Metabolism | steroid metabolic process | 4.69E-03 | 9.30E-04 | 14223 | 209 | 1066 | 29 |
| Cell Metabolism | Cell Metabolism | pyruvate metabolic process | 5.04E-03 | 1.00E-03 | 14223 | 29 | 1066 | 8 |
| Cell Metabolism | Cell Metabolism | regulation of steroid biosynthetic process | 5.04E-03 | 1.00E-03 | 14223 | 29 | 1066 | 8 |
| Cell Metabolism | Cell Metabolism | positive regulation of steroid metabolic process | 5.22E-03 | 1.05E-03 | 14223 | 17 | 1066 | 6 |
| Cell Metabolism | Cell Metabolism | dicarboxylic acid metabolic process | 5.22E-03 | 1.05E-03 | 14223 | 43 | 1066 | 10 |
| Cell Metabolism | Cell Metabolism | regulation of phosphatase activity | 5.37E-03 | 1.10E-03 | 14223 | 23 | 1066 | 7 |
| Cell Metabolism | Cell Metabolism | nucleobase metabolic process | 5.37E-03 | 1.10E-03 | 14223 | 23 | 1066 | 7 |
| Cell Metabolism | Cell Metabolism | oxaloacetate metabolic process | 5.77E-03 | 1.19E-03 | 14223 | 12 | 1066 | 5 |
| Cell Metabolism | Cell Metabolism | sterol metabolic process | 6.21E-03 | 1.29E-03 | 14223 | 101 | 1066 | 17 |
| Cell Metabolism | Cell Metabolism | receptor metabolic process | 6.33E-03 | 1.32E-03 | 14223 | 37 | 1066 | 9 |
| Cell Metabolism | Cell Metabolism | hormone metabolic process | 6.33E-03 | 1.32E-03 | 14223 | 110 | 1066 | 18 |
| Cell Metabolism | Cell Metabolism | negative regulation of nitrogen compound metabolic process | 6.59E-03 | 1.38E-03 | 14223 | 563 | 1066 | 62 |
| Cell Metabolism | Cell Metabolism | regulation of lipase activity | 6.68E-03 | 1.40E-03 | 14223 | 93 | 1066 | 16 |
| Cell Metabolism | Cell Metabolism | positive regulation of peptidase activity | 6.81E-03 | 1.43E-03 | 14223 | 68 | 1066 | 13 |
| Cell Metabolism | Cell Metabolism | heme catabolic process | 7.32E-03 | 1.59E-03 | 14223 | 4 | 1066 | 3 |
| Cell Metabolism | Cell Metabolism | pigment catabolic process | 7.32E-03 | 1.59E-03 | 14223 | 4 | 1066 | 3 |
| Cell Metabolism | Cell Metabolism | L-serine biosynthetic process | 7.32E-03 | 1.59E-03 | 14223 | 4 | 1066 | 3 |
| Cell Metabolism | Cell Metabolism | peptidyl-serine phosphorylation | 7.43E-03 | 1.61E-03 | 14223 | 31 | 1066 | 8 |
| Cell Metabolism | Cell Metabolism | positive regulation of proteolysis | 7.43E-03 | 1.62E-03 | 14223 | 38 | 1066 | 9 |
| Cell Metabolism | Cell Metabolism | carbohydrate metabolic process | 7.57E-03 | 1.65E-03 | 14223 | 523 | 1066 | 58 |
| Cell Metabolism | Cell Metabolism | regulation of protein amino acid dephosphorylation | 7.74E-03 | 1.72E-03 | 14223 | 8 | 1066 | 4 |
| Cell Metabolism | Cell Metabolism | negative regulation of nucleobase, nucleoside, nucleotide and nucleic acid metabolic process | 7.74E-03 | 1.71E-03 | 14223 | 557 | 1066 | 61 |
| Cell Metabolism | Cell Metabolism | positive regulation of lipid biosynthetic process | 8.39E-03 | 1.89E-03 | 14223 | 25 | 1066 | 7 |
| Cell Metabolism | Cell Metabolism | negative regulation of phosphorylation | 8.48E-03 | 1.91E-03 | 14223 | 54 | 1066 | 11 |
| Cell Metabolism | Cell Metabolism | cellular aldehyde metabolic process | 8.90E-03 | 2.01E-03 | 14223 | 32 | 1066 | 8 |
| Cell Metabolism | Cell Metabolism | regulation of fatty acid oxidation | 8.90E-03 | 2.01E-03 | 14223 | 32 | 1066 | 8 |
| Cell Metabolism | Cell Metabolism | hexose catabolic process | 9.76E-03 | 2.22E-03 | 14223 | 63 | 1066 | 12 |
| Cell Metabolism | Cell Metabolism | cofactor catabolic process | 1.09E-02 | 2.48E-03 | 14223 | 33 | 1066 | 8 |
| Cell Metabolism | Cell Metabolism | cellular carbohydrate catabolic process | 1.13E-02 | 2.59E-03 | 14223 | 81 | 1066 | 14 |
| Cell Metabolism | Cell Metabolism | peptidyl-serine modification | 1.22E-02 | 2.84E-03 | 14223 | 41 | 1066 | 9 |
| Cell Metabolism | Cell Metabolism | L-serine metabolic process | 1.23E-02 | 2.91E-03 | 14223 | 9 | 1066 | 4 |
| Cell Metabolism | Cell Metabolism | protein catabolic process | 1.25E-02 | 2.97E-03 | 14223 | 340 | 1066 | 40 |
| Cell Metabolism | Cell Metabolism | monosaccharide catabolic process | 1.38E-02 | 3.33E-03 | 14223 | 66 | 1066 | 12 |
| Cell Metabolism | Cell Metabolism | oxidoreduction coenzyme metabolic process | 1.63E-02 | 4.06E-03 | 14223 | 51 | 1066 | 10 |
| Cell Metabolism | Cell Metabolism | regulation of primary metabolic process | 1.71E-02 | 4.29E-03 | 14223 | 3554 | 1066 | 303 |
| Cell Metabolism | Cell Metabolism | regulation of steroid metabolic process | 2.07E-02 | 5.48E-03 | 14223 | 45 | 1066 | 9 |
| Cell Metabolism | Cell Metabolism | positive regulation of protein catabolic process | 2.07E-02 | 5.48E-03 | 14223 | 45 | 1066 | 9 |
| Cell Metabolism | Cell Metabolism | regulation of cellular ketone metabolic process | 2.28E-02 | 6.24E-03 | 14223 | 80 | 1066 | 13 |
| Cell Metabolism | Cell Metabolism | tetrapyrrole catabolic process | 2.47E-02 | 7.07E-03 | 14223 | 6 | 1066 | 3 |
| Cell Metabolism | Cell Metabolism | porphyrin catabolic process | 2.47E-02 | 7.07E-03 | 14223 | 6 | 1066 | 3 |
| Cell Metabolism | Cell Metabolism | cellular macromolecule catabolic process | 2.56E-02 | 7.35E-03 | 14223 | 436 | 1066 | 47 |

| | | | | | | | | |
|---|---|---|---|---|---|---|---|---|
| Cell Metabolism | Cell Metabolism | cellular amino acid metabolic process | 3.05E-02 | 8.84E-03 | 14223 | 233 | 1066 | 28 |
| Cell Metabolism | Cell Metabolism | C21-steroid hormone metabolic process | 3.23E-02 | 9.54E-03 | 14223 | 12 | 1066 | 4 |
| Cell Metabolism | Cell Metabolism | regulation of peptidase activity | 3.27E-02 | 9.65E-03 | 14223 | 103 | 1066 | 15 |
| Cell Metabolism | Cell Metabolism | regulation of lipid catabolic process | 3.35E-02 | 9.92E-03 | 14223 | 33 | 1066 | 7 |
| Cell Metabolism | Cell Metabolism | cofactor metabolic process | 3.43E-02 | 1.02E-02 | 14223 | 204 | 1066 | 25 |
| Cell Metabolism | Cell Metabolism | cellular amino acid and derivative metabolic process | 3.45E-02 | 1.02E-02 | 14223 | 366 | 1066 | 40 |
| Cell Metabolism | Cell Metabolism | regulation of fatty acid metabolic process | 3.47E-02 | 1.03E-02 | 14223 | 58 | 1066 | 10 |
| Cell Metabolism | Cell Metabolism | cellular amine metabolic process | 3.61E-02 | 1.07E-02 | 14223 | 312 | 1066 | 35 |
| Cell Metabolism | Cell Metabolism | cellular protein catabolic process | 3.84E-02 | 1.18E-02 | 14223 | 314 | 1066 | 35 |
| Cell Metabolism | Cell Metabolism | carboxylic acid biosynthetic process | 4.08E-02 | 1.25E-02 | 14223 | 166 | 1066 | 21 |
| Cell Metabolism | Cell Metabolism | organic acid biosynthetic process | 4.08E-02 | 1.25E-02 | 14223 | 166 | 1066 | 21 |
| Cell Metabolism | Cell Metabolism | positive regulation of phospholipase activity | 4.20E-02 | 1.31E-02 | 14223 | 69 | 1066 | 11 |
| Cell Metabolism | Cell Metabolism | small molecule biosynthetic process | 5.03E-02 | 1.68E-02 | 14223 | 446 | 1066 | 46 |
| Cell Metabolism | Cell Metabolism | proteolysis involved in cellular protein catabolic process | 5.05E-02 | 1.69E-02 | 14223 | 311 | 1066 | 34 |
| Cell Metabolism | Cell Metabolism | nitrogen compound metabolic process | 5.14E-02 | 1.73E-02 | 14223 | 2191 | 1066 | 189 |
| Cell Metabolism | Cell Metabolism | cellular amino acid biosynthetic process | 5.27E-02 | 1.80E-02 | 14223 | 54 | 1066 | 9 |
| Cell Metabolism | Cell Metabolism | heterocycle catabolic process | 6.17E-02 | 2.14E-02 | 14223 | 74 | 1066 | 11 |
| Cell Metabolism | Cell Metabolism | coenzyme metabolic process | 6.22E-02 | 2.16E-02 | 14223 | 154 | 1066 | 19 |
| Cell Metabolism | Cell Metabolism | regulation of Rho GTPase activity | 6.28E-02 | 2.19E-02 | 14223 | 30 | 1066 | 6 |
| Cell Metabolism | Cell Metabolism | positive regulation of lipase activity | 7.13E-02 | 2.56E-02 | 14223 | 76 | 1066 | 11 |
| Cell Metabolism | Cell Metabolism | cellular carbohydrate metabolic process | 7.72E-02 | 2.79E-02 | 14223 | 380 | 1066 | 39 |
| Cell Metabolism | Cell Metabolism | negative regulation of RNA metabolic process | 7.97E-02 | 2.89E-02 | 14223 | 404 | 1066 | 41 |
| Cell Metabolism | Cell Metabolism | positive regulation of Ras GTPase activity | 8.04E-02 | 3.01E-02 | 14223 | 24 | 1066 | 5 |
| Cell Metabolism | Cell Metabolism | proteasomal protein catabolic process | 8.14E-02 | 3.08E-02 | 14223 | 139 | 1066 | 17 |
| Cell Metabolism | Cell Metabolism | proteasomal ubiquitin-dependent protein catabolic process | 8.14E-02 | 3.08E-02 | 14223 | 139 | 1066 | 17 |
| Cell Metabolism | Cell Metabolism | regulation of endopeptidase activity | 8.16E-02 | 3.09E-02 | 14223 | 98 | 1066 | 13 |
| Cell Metabolism | Cell Metabolism | cellular biosynthetic process | 8.71E-02 | 3.31E-02 | 14223 | 1689 | 1066 | 146 |
| Cell Metabolism | Cell Metabolism | lipid metabolic process | 9.84E-02 | 3.83E-02 | 14223 | 830 | 1066 | 76 |
| Cell Metabolism | Cell Metabolism | biosynthetic process | 9.89E-02 | 3.86E-02 | 14223 | 1799 | 1066 | 154 |
| Cell Metabolism | Cell Metabolism | regulation of macromolecule biosynthetic process | 1.21E-01 | 5.08E-02 | 14223 | 2878 | 1066 | 237 |
| Cell Metabolism | Cell Metabolism | heme metabolic process | 1.30E-01 | 5.44E-02 | 14223 | 28 | 1066 | 5 |
| Cell Metabolism | Cell Metabolism | cellular nitrogen compound catabolic process | 1.44E-01 | 6.14E-02 | 14223 | 67 | 1066 | 9 |
| Cell Metabolism | Cell Metabolism | positive regulation of GTPase activity | 1.45E-01 | 6.19E-02 | 14223 | 29 | 1066 | 5 |
| Cell Metabolism | Cell Metabolism | amine biosynthetic process | 1.48E-01 | 6.83E-02 | 14223 | 89 | 1066 | 11 |
| Cell Metabolism | Cell Metabolism | macromolecule biosynthetic process | 1.48E-01 | 6.89E-02 | 14223 | 1056 | 1066 | 92 |
| Cell Metabolism | Cell Metabolism | DNA metabolic process | 1.48E-01 | 6.61E-02 | 14223 | 514 | 1066 | 48 |
| Cell Metabolism | Cell Metabolism | nucleobase, nucleoside and nucleotide metabolic process | 1.50E-01 | 7.61E-02 | 14223 | 305 | 1066 | 30 |
| Cell Metabolism | Cell Metabolism | regulation of biosynthetic process | 1.58E-01 | 8.06E-02 | 14223 | 3047 | 1066 | 247 |
| Cell Metabolism | Cell Metabolism | cellular nitrogen compound metabolic process | 1.71E-01 | 8.83E-02 | 14223 | 2074 | 1066 | 171 |
| Cell Metabolism | Cell Metabolism | steroid biosynthetic process | 2.05E-01 | 1.09E-01 | 14223 | 86 | 1066 | 10 |
| Cell Metabolism | Cell Metabolism | cellular macromolecule biosynthetic process | 2.12E-01 | 1.13E-01 | 14223 | 1035 | 1066 | 88 |
| Cell Metabolism | Cell Metabolism | pigment metabolic process | 2.26E-01 | 1.24E-01 | 14223 | 56 | 1066 | 7 |
| Cell Metabolism | Cell Metabolism | heterocycle metabolic process | 2.35E-01 | 1.37E-01 | 14223 | 337 | 1066 | 31 |
| Cell Metabolism | Cell Metabolism | tetrapyrrole metabolic process | 2.35E-01 | 1.40E-01 | 14223 | 37 | 1066 | 5 |
| Cell Metabolism | Cell Metabolism | porphyrin metabolic process | 2.35E-01 | 1.40E-01 | 14223 | 37 | 1066 | 5 |
| Cell Metabolism | Cell Metabolism | lipid biosynthetic process | 2.45E-01 | 1.52E-01 | 14223 | 341 | 1066 | 31 |
| Cell Metabolism | Cell Metabolism | nucleoside phosphate metabolic process | 2.49E-01 | 1.55E-01 | 14223 | 281 | 1066 | 26 |
| Cell Metabolism | Cell Metabolism | nucleotide metabolic process | 2.49E-01 | 1.55E-01 | 14223 | 281 | 1066 | 26 |
| Cell Metabolism | Cell Metabolism | regulation of cellular biosynthetic process | 2.76E-01 | 1.75E-01 | 14223 | 3022 | 1066 | 239 |
| Cell Metabolism | Cell Metabolism | cellular aromatic compound metabolic process | 2.99E-01 | 1.91E-01 | 14223 | 144 | 1066 | 14 |

| | | | | | | | | |
|---|---|---|---|---|---|---|---|---|
| Cell Metabolism | Cell Metabolism | cellular nitrogen compound biosynthetic process | 3.11E-01 | 2.14E-01 | 14223 | 331 | 1066 | 29 |
| Cell Metabolism | Cell Metabolism | hormone biosynthetic process | 3.12E-01 | 2.16E-01 | 14223 | 32 | 1066 | 4 |
| Cell Metabolism | Cell Metabolism | modification-dependent protein catabolic process | 3.14E-01 | 2.17E-01 | 14223 | 282 | 1066 | 25 |
| Cell Metabolism | Cell Metabolism | modification-dependent macromolecule catabolic process | 3.14E-01 | 2.17E-01 | 14223 | 282 | 1066 | 25 |
| Cell Metabolism | Cell Metabolism | regulation of nucleotide catabolic process | 3.67E-01 | 2.72E-01 | 14223 | 155 | 1066 | 14 |
| Cell Metabolism | Cell Metabolism | ubiquitin-dependent protein catabolic process | 4.28E-01 | 3.35E-01 | 14223 | 277 | 1066 | 23 |
| Cell Metabolism | Cell Metabolism | regulation of nucleotide metabolic process | 5.22E-01 | 4.41E-01 | 14223 | 280 | 1066 | 22 |
| Cell Metabolism | Cell Metabolism | nucleobase, nucleoside, nucleotide and nucleic acid metabolic process | 5.36E-01 | 4.54E-01 | 14223 | 1780 | 1066 | 135 |
| Cell Metabolism | Cell Metabolism | regulation of nitrogen compound metabolic process | 5.76E-01 | 5.06E-01 | 14223 | 3039 | 1066 | 228 |
| Cell Metabolism | Cell Metabolism | regulation of nucleobase, nucleoside, nucleotide and nucleic acid metabolic process | 7.12E-01 | 6.55E-01 | 14223 | 3011 | 1066 | 221 |
| Cell Metabolism | Cell Metabolism | nucleic acid metabolic process | 7.36E-01 | 6.83E-01 | 14223 | 1456 | 1066 | 105 |
| Cell Metabolism | Cell Metabolism | regulation of GTP catabolic process | 7.71E-01 | 7.23E-01 | 14223 | 139 | 1066 | 9 |
| Cell Metabolism | Cell Metabolism | regulation of RNA metabolic process | 9.22E-01 | 8.97E-01 | 14223 | 1852 | 1066 | 126 |
| Cell Metabolism | Cell Metabolism | regulation of Ras GTPase activity | 9.23E-01 | 8.99E-01 | 14223 | 121 | 1066 | 6 |
| Cell Movement | Cell Movement | locomotion | 1.97E-40 | 9.10E-43 | 14223 | 446 | 1066 | 128 |
| Cell Movement | Cell Movement | chemotaxis | 2.81E-40 | 1.42E-42 | 14223 | 171 | 1066 | 78 |
| Cell Movement | Cell Movement | taxis | 2.81E-40 | 1.42E-42 | 14223 | 171 | 1066 | 78 |
| Cell Movement | Cell Movement | cellular component movement | 9.54E-28 | 9.22E-30 | 14223 | 480 | 1066 | 114 |
| Cell Movement | Cell Movement | regulation of localization | 3.91E-27 | 4.19E-29 | 14223 | 734 | 1066 | 146 |
| Cell Movement | Cell Movement | positive regulation of locomotion | 2.01E-26 | 2.28E-28 | 14223 | 139 | 1066 | 57 |
| Cell Movement | Cell Movement | cell migration | 3.49E-26 | 4.18E-28 | 14223 | 287 | 1066 | 83 |
| Cell Movement | Cell Movement | regulation of locomotion | 1.36E-25 | 1.77E-27 | 14223 | 243 | 1066 | 75 |
| Cell Movement | Cell Movement | positive regulation of cellular component movement | 1.25E-24 | 1.76E-26 | 14223 | 139 | 1066 | 55 |
| Cell Movement | Cell Movement | regulation of cellular component movement | 2.26E-24 | 3.33E-26 | 14223 | 241 | 1066 | 73 |
| Cell Movement | Cell Movement | positive regulation of cell migration | 1.40E-23 | 2.29E-25 | 14223 | 130 | 1066 | 52 |
| Cell Movement | Cell Movement | regulation of cell migration | 1.58E-23 | 2.63E-25 | 14223 | 218 | 1066 | 68 |
| Cell Movement | Cell Movement | cell motility | 1.81E-23 | 3.12E-25 | 14223 | 320 | 1066 | 84 |
| Cell Movement | Cell Movement | localization of cell | 1.81E-23 | 3.12E-25 | 14223 | 320 | 1066 | 84 |
| Cell Movement | Cell Movement | leukocyte migration | 9.64E-22 | 1.98E-23 | 14223 | 63 | 1066 | 35 |
| Cell Movement | Cell Movement | cell chemotaxis | 1.30E-21 | 2.70E-23 | 14223 | 49 | 1066 | 31 |
| Cell Movement | Cell Movement | leukocyte chemotaxis | 1.10E-18 | 2.51E-20 | 14223 | 43 | 1066 | 27 |
| Cell Movement | Cell Movement | neutrophil chemotaxis | 1.48E-08 | 9.10E-10 | 14223 | 19 | 1066 | 12 |
| Cell Movement | Cell Movement | regulation of chemotaxis | 2.63E-08 | 1.72E-09 | 14223 | 51 | 1066 | 19 |
| Cell Movement | Cell Movement | positive regulation of chemotaxis | 1.96E-07 | 1.52E-08 | 14223 | 41 | 1066 | 16 |
| Cell Movement | Cell Movement | lymphocyte chemotaxis | 1.14E-05 | 1.15E-06 | 14223 | 7 | 1066 | 6 |
| Cell Movement | Cell Movement | regulation of positive chemotaxis | 1.48E-05 | 1.51E-06 | 14223 | 22 | 1066 | 10 |
| Cell Movement | Cell Movement | cellular extravasation | 3.06E-05 | 3.29E-06 | 14223 | 11 | 1066 | 7 |
| Cell Movement | Cell Movement | positive regulation of endothelial cell migration | 5.49E-05 | 6.18E-06 | 14223 | 25 | 1066 | 10 |
| Cell Movement | Cell Movement | induction of positive chemotaxis | 6.44E-05 | 7.38E-06 | 14223 | 12 | 1066 | 7 |
| Cell Movement | Cell Movement | positive regulation of leukocyte migration | 7.18E-05 | 8.32E-06 | 14223 | 31 | 1066 | 11 |
| Cell Movement | Cell Movement | positive regulation of positive chemotaxis | 7.90E-05 | 9.29E-06 | 14223 | 21 | 1066 | 9 |
| Cell Movement | Cell Movement | regulation of leukocyte migration | 1.77E-04 | 2.30E-05 | 14223 | 40 | 1066 | 12 |
| Cell Movement | Cell Movement | regulation of endothelial cell migration | 2.90E-04 | 3.96E-05 | 14223 | 42 | 1066 | 12 |
| Cell Movement | Cell Movement | monocyte chemotaxis | 3.14E-04 | 4.33E-05 | 14223 | 7 | 1066 | 5 |
| Cell Movement | Cell Movement | regulation of smooth muscle cell migration | 3.83E-04 | 5.41E-05 | 14223 | 20 | 1066 | 8 |
| Cell Movement | Cell Movement | neural crest cell migration | 3.83E-04 | 5.41E-05 | 14223 | 20 | 1066 | 8 |
| Cell Movement | Cell Movement | macrophage chemotaxis | 4.09E-04 | 5.82E-05 | 14223 | 11 | 1066 | 6 |
| Cell Movement | Cell Movement | negative regulation of cell migration | 6.87E-04 | 1.02E-04 | 14223 | 67 | 1066 | 15 |
| Cell Movement | Cell Movement | regulation of epithelial cell migration | 1.17E-03 | 1.89E-04 | 14223 | 13 | 1066 | 6 |
| Cell Movement | Cell Movement | negative regulation of locomotion | 1.44E-03 | 2.40E-04 | 14223 | 72 | 1066 | 15 |

| | | | | | | | | |
|---|---|---|---|---|---|---|---|---|
| Cell Movement | Cell Movement | endothelial cell migration | 1.92E-03 | 3.31E-04 | 14223 | 25 | 1066 | 8 |
| Cell Movement | Cell Movement | positive regulation of epithelial cell migration | 2.38E-03 | 4.29E-04 | 14223 | 10 | 1066 | 5 |
| Cell Movement | Cell Movement | positive regulation of smooth muscle cell migration | 2.38E-03 | 4.29E-04 | 14223 | 10 | 1066 | 5 |
| Cell Movement | Cell Movement | ameboidal cell migration | 2.91E-03 | 5.37E-04 | 14223 | 33 | 1066 | 9 |
| Cell Movement | Cell Movement | regulation of leukocyte chemotaxis | 4.04E-03 | 7.77E-04 | 14223 | 28 | 1066 | 8 |
| Cell Movement | Cell Movement | positive regulation of leukocyte chemotaxis | 5.37E-03 | 1.10E-03 | 14223 | 23 | 1066 | 7 |
| Cell Movement | Cell Movement | cellular response to hydrogen peroxide | 8.39E-03 | 1.89E-03 | 14223 | 25 | 1066 | 7 |
| Cell Movement | Cell Movement | cellular localization | 5.23E-01 | 4.42E-01 | 14223 | 980 | 1066 | 75 |
| Cell Proliferation | Cell Proliferation | positive regulation of cell proliferation | 2.26E-50 | 5.22E-53 | 14223 | 463 | 1066 | 144 |
| Cell Proliferation | Cell Proliferation | regulation of cell proliferation | 2.16E-49 | 6.79E-52 | 14223 | 854 | 1066 | 200 |
| Cell Proliferation | Cell Proliferation | positive regulation of cell division | 6.88E-17 | 1.82E-18 | 14223 | 41 | 1066 | 25 |
| Cell Proliferation | Cell Proliferation | regulation of cell division | 3.03E-15 | 8.77E-17 | 14223 | 50 | 1066 | 26 |
| Cell Proliferation | Cell Proliferation | negative regulation of cell proliferation | 6.62E-15 | 1.98E-16 | 14223 | 383 | 1066 | 78 |
| Cell Proliferation | Cell Proliferation | regulation of leukocyte proliferation | 2.36E-14 | 7.72E-16 | 14223 | 97 | 1066 | 35 |
| Cell Proliferation | Cell Proliferation | regulation of mononuclear cell proliferation | 1.11E-13 | 3.83E-15 | 14223 | 96 | 1066 | 34 |
| Cell Proliferation | Cell Proliferation | cell proliferation | 1.16E-13 | 4.03E-15 | 14223 | 436 | 1066 | 82 |
| Cell Proliferation | Cell Proliferation | regulation of lymphocyte proliferation | 5.05E-13 | 1.87E-14 | 14223 | 95 | 1066 | 33 |
| Cell Proliferation | Cell Proliferation | positive regulation of endothelial cell proliferation | 3.85E-11 | 1.71E-12 | 14223 | 33 | 1066 | 18 |
| Cell Proliferation | Cell Proliferation | regulation of epithelial cell proliferation | 1.65E-09 | 8.85E-11 | 14223 | 87 | 1066 | 27 |
| Cell Proliferation | Cell Proliferation | regulation of smooth muscle cell proliferation | 3.76E-09 | 2.11E-10 | 14223 | 46 | 1066 | 19 |
| Cell Proliferation | Cell Proliferation | positive regulation of cell cycle | 4.53E-09 | 2.58E-10 | 14223 | 73 | 1066 | 24 |
| Cell Proliferation | Cell Proliferation | regulation of endothelial cell proliferation | 8.66E-09 | 5.07E-10 | 14223 | 48 | 1066 | 19 |
| Cell Proliferation | Cell Proliferation | regulation of T cell proliferation | 1.71E-08 | 1.06E-09 | 14223 | 66 | 1066 | 22 |
| Cell Proliferation | Cell Proliferation | positive regulation of epithelial cell proliferation | 1.88E-08 | 1.19E-09 | 14223 | 45 | 1066 | 18 |
| Cell Proliferation | Cell Proliferation | positive regulation of leukocyte proliferation | 2.27E-08 | 1.47E-09 | 14223 | 67 | 1066 | 22 |
| Cell Proliferation | Cell Proliferation | negative regulation of leukocyte proliferation | 3.05E-08 | 2.02E-09 | 14223 | 32 | 1066 | 15 |
| Cell Proliferation | Cell Proliferation | negative regulation of lymphocyte proliferation | 3.05E-08 | 2.02E-09 | 14223 | 32 | 1066 | 15 |
| Cell Proliferation | Cell Proliferation | negative regulation of mononuclear cell proliferation | 3.05E-08 | 2.02E-09 | 14223 | 32 | 1066 | 15 |
| Cell Proliferation | Cell Proliferation | positive regulation of mononuclear cell proliferation | 8.94E-08 | 6.61E-09 | 14223 | 66 | 1066 | 21 |
| Cell Proliferation | Cell Proliferation | positive regulation of lymphocyte proliferation | 3.51E-07 | 2.87E-08 | 14223 | 65 | 1066 | 20 |
| Cell Proliferation | Cell Proliferation | regulation of B cell proliferation | 6.51E-07 | 5.44E-08 | 14223 | 34 | 1066 | 14 |
| Cell Proliferation | Cell Proliferation | positive regulation of nuclear division | 6.94E-06 | 6.66E-07 | 14223 | 25 | 1066 | 11 |
| Cell Proliferation | Cell Proliferation | positive regulation of mitosis | 6.94E-06 | 6.66E-07 | 14223 | 25 | 1066 | 11 |
| Cell Proliferation | Cell Proliferation | positive regulation of smooth muscle cell proliferation | 7.41E-06 | 7.19E-07 | 14223 | 30 | 1066 | 12 |
| Cell Proliferation | Cell Proliferation | negative regulation of T cell proliferation | 1.08E-05 | 1.08E-06 | 14223 | 26 | 1066 | 11 |
| Cell Proliferation | Cell Proliferation | positive regulation of fibroblast proliferation | 1.10E-05 | 1.09E-06 | 14223 | 31 | 1066 | 12 |
| Cell Proliferation | Cell Proliferation | positive regulation of cyclin-dependent protein kinase activity involved in G1/S | 1.14E-05 | 1.15E-06 | 14223 | 7 | 1066 | 6 |
| Cell Proliferation | Cell Proliferation | regulation of cyclin-dependent protein kinase activity involved by G1/S | 1.14E-05 | 1.15E-06 | 14223 | 7 | 1066 | 6 |
| Cell Proliferation | Cell Proliferation | mononuclear cell proliferation | 3.56E-05 | 3.90E-06 | 14223 | 40 | 1066 | 13 |
| Cell Proliferation | Cell Proliferation | leukocyte proliferation | 4.77E-05 | 5.32E-06 | 14223 | 41 | 1066 | 13 |
| Cell Proliferation | Cell Proliferation | regulation of mitosis | 5.07E-05 | 5.69E-06 | 14223 | 60 | 1066 | 16 |
| Cell Proliferation | Cell Proliferation | regulation of nuclear division | 5.07E-05 | 5.69E-06 | 14223 | 60 | 1066 | 16 |
| Cell Proliferation | Cell Proliferation | positive regulation of mesenchymal cell proliferation | 7.90E-05 | 9.29E-06 | 14223 | 21 | 1066 | 9 |
| Cell Proliferation | Cell Proliferation | positive regulation of B cell proliferation | 7.93E-05 | 9.38E-06 | 14223 | 26 | 1066 | 10 |
| Cell Proliferation | Cell Proliferation | positive regulation of cell cycle process | 1.04E-04 | 1.27E-05 | 14223 | 44 | 1066 | 13 |
| Cell Proliferation | Cell Proliferation | lymphocyte proliferation | 1.05E-04 | 1.28E-05 | 14223 | 38 | 1066 | 12 |
| Cell Proliferation | Cell Proliferation | regulation of cell cycle | 1.18E-04 | 1.46E-05 | 14223 | 448 | 1066 | 59 |
| Cell Proliferation | Cell Proliferation | negative regulation of smooth muscle cell proliferation | 2.13E-04 | 2.80E-05 | 14223 | 14 | 1066 | 7 |
| Cell Proliferation | Cell Proliferation | regulation of mesenchymal cell proliferation | 2.51E-04 | 3.36E-05 | 14223 | 24 | 1066 | 9 |
| Cell Proliferation | Cell Proliferation | regulation of cell cycle process | 2.57E-04 | 3.45E-05 | 14223 | 139 | 1066 | 25 |

| | | | | | | | | |
|---|---|---|---|---|---|---|---|---|
| Cell Proliferation | Cell Proliferation | positive regulation of T cell proliferation | 2.90E-04 | 3.96E-05 | 14223 | 42 | 1066 | 12 |
| Cell Proliferation | Cell Proliferation | regulation of mitotic cell cycle | 7.73E-04 | 1.17E-04 | 14223 | 176 | 1066 | 28 |
| Cell Proliferation | Cell Proliferation | G1/S transition of mitotic cell cycle | 8.72E-04 | 1.34E-04 | 14223 | 54 | 1066 | 13 |
| Cell Proliferation | Cell Proliferation | epithelial cell proliferation | 1.08E-03 | 1.72E-04 | 14223 | 23 | 1066 | 8 |
| Cell Proliferation | Cell Proliferation | negative regulation of epithelial cell proliferation | 1.09E-03 | 1.74E-04 | 14223 | 35 | 1066 | 10 |
| Cell Proliferation | Cell Proliferation | B cell proliferation | 1.17E-03 | 1.89E-04 | 14223 | 13 | 1066 | 6 |
| Cell Proliferation | Cell Proliferation | positive regulation of ubiquitin-protein ligase activity involved in mitotic cell cycle | 1.36E-03 | 2.24E-04 | 14223 | 64 | 1066 | 14 |
| Cell Proliferation | Cell Proliferation | positive regulation of granule cell precursor proliferation | 1.37E-03 | 2.29E-04 | 14223 | 9 | 1066 | 5 |
| Cell Proliferation | Cell Proliferation | regulation of granule cell precursor proliferation | 1.37E-03 | 2.29E-04 | 14223 | 9 | 1066 | 5 |
| Cell Proliferation | Cell Proliferation | positive regulation of cyclin-dependent protein kinase activity | 1.82E-03 | 3.11E-04 | 14223 | 14 | 1066 | 6 |
| Cell Proliferation | Cell Proliferation | regulation of ubiquitin-protein ligase activity involved in mitotic cell cycle | 2.40E-03 | 4.35E-04 | 14223 | 68 | 1066 | 14 |
| Cell Proliferation | Cell Proliferation | negative regulation of ubiquitin-protein ligase activity involved in mitotic cell cycle | 3.10E-03 | 5.74E-04 | 14223 | 62 | 1066 | 13 |
| Cell Proliferation | Cell Proliferation | anaphase-promoting complex-dependent proteasomal ubiquitin-dependent protein catabolic process | 3.58E-03 | 6.74E-04 | 14223 | 63 | 1066 | 13 |
| Cell Proliferation | Cell Proliferation | negative regulation of cell cycle | 7.32E-03 | 1.58E-03 | 14223 | 177 | 1066 | 25 |
| Cell Proliferation | Cell Proliferation | negative regulation of B cell proliferation | 7.74E-03 | 1.72E-03 | 14223 | 8 | 1066 | 4 |
| Cell Proliferation | Cell Proliferation | regulation of activated T cell proliferation | 8.11E-03 | 1.81E-03 | 14223 | 13 | 1066 | 5 |
| Cell Proliferation | Cell Proliferation | interphase of mitotic cell cycle | 1.79E-02 | 4.49E-03 | 14223 | 104 | 1066 | 16 |
| Cell Proliferation | Cell Proliferation | cell cycle process | 1.88E-02 | 4.84E-03 | 14223 | 583 | 1066 | 61 |
| Cell Proliferation | Cell Proliferation | regulation of cyclin-dependent protein kinase activity | 2.15E-02 | 5.87E-03 | 14223 | 62 | 1066 | 11 |
| Cell Proliferation | Cell Proliferation | interphase | 2.36E-02 | 6.51E-03 | 14223 | 108 | 1066 | 16 |
| Cell Proliferation | Cell Proliferation | mitotic cell cycle | 8.04E-02 | 2.99E-02 | 14223 | 382 | 1066 | 39 |
| Cell Proliferation | Cell Proliferation | cell cycle | 2.35E-01 | 1.37E-01 | 14223 | 795 | 1066 | 68 |
| Cell Proliferation | Cell Proliferation | cell cycle phase | 4.58E-01 | 3.60E-01 | 14223 | 436 | 1066 | 35 |
| Cell Signalling | Cell Signalling | enzyme linked receptor protein signaling pathway | 4.45E-43 | 1.87E-45 | 14223 | 352 | 1066 | 116 |
| Cell Signalling | Cell Signalling | cell surface receptor linked signaling pathway | 7.66E-43 | 3.38E-45 | 14223 | 1283 | 1066 | 242 |
| Cell Signalling | Cell Signalling | signaling pathway | 2.56E-37 | 1.45E-39 | 14223 | 2098 | 1066 | 318 |
| Cell Signalling | Cell Signalling | regulation of response to stimulus | 2.97E-35 | 1.87E-37 | 14223 | 530 | 1066 | 133 |
| Cell Signalling | Cell Signalling | signal transduction | 1.76E-31 | 1.33E-33 | 14223 | 1880 | 1066 | 282 |
| Cell Signalling | Cell Signalling | positive regulation of signaling pathway | 1.74E-30 | 1.46E-32 | 14223 | 382 | 1066 | 104 |
| Cell Signalling | Cell Signalling | transmembrane receptor protein tyrosine kinase signaling pathway | 3.90E-27 | 4.09E-29 | 14223 | 225 | 1066 | 74 |
| Cell Signalling | Cell Signalling | signal transmission | 2.35E-26 | 2.76E-28 | 14223 | 2159 | 1066 | 296 |
| Cell Signalling | Cell Signalling | positive regulation of signal transduction | 2.14E-24 | 3.10E-26 | 14223 | 278 | 1066 | 79 |
| Cell Signalling | Cell Signalling | positive regulation of signaling process | 7.14E-24 | 1.13E-25 | 14223 | 283 | 1066 | 79 |
| Cell Signalling | Cell Signalling | positive regulation of intracellular protein kinase cascade | 1.58E-23 | 2.65E-25 | 14223 | 224 | 1066 | 69 |
| Cell Signalling | Cell Signalling | regulation of intracellular protein kinase cascade | 6.58E-23 | 1.23E-24 | 14223 | 326 | 1066 | 84 |
| Cell Signalling | Cell Signalling | regulation of signaling pathway | 1.77E-21 | 3.71E-23 | 14223 | 1004 | 1066 | 165 |
| Cell Signalling | Cell Signalling | transmembrane receptor protein serine/threonine kinase signaling pathway | 2.19E-20 | 4.88E-22 | 14223 | 108 | 1066 | 44 |
| Cell Signalling | Cell Signalling | regulation of signal transduction | 1.17E-18 | 2.70E-20 | 14223 | 776 | 1066 | 133 |
| Cell Signalling | Cell Signalling | regulation of signaling process | 2.05E-18 | 4.83E-20 | 14223 | 781 | 1066 | 133 |
| Cell Signalling | Cell Signalling | response to steroid hormone stimulus | 1.49E-17 | 3.70E-19 | 14223 | 227 | 1066 | 61 |
| Cell Signalling | Cell Signalling | positive regulation of JAK-STAT cascade | 1.45E-14 | 4.47E-16 | 14223 | 27 | 1066 | 19 |
| Cell Signalling | Cell Signalling | transforming growth factor beta receptor signaling pathway | 1.52E-14 | 4.78E-16 | 14223 | 57 | 1066 | 27 |
| Cell Signalling | Cell Signalling | regulation of JAK-STAT cascade | 1.07E-13 | 3.65E-15 | 14223 | 36 | 1066 | 21 |
| Cell Signalling | Cell Signalling | positive regulation of tyrosine phosphorylation of STAT protein | 1.24E-13 | 4.32E-15 | 14223 | 23 | 1066 | 17 |
| Cell Signalling | Cell Signalling | regulation of tyrosine phosphorylation of STAT protein | 2.43E-13 | 8.84E-15 | 14223 | 30 | 1066 | 19 |
| Cell Signalling | Cell Signalling | positive regulation of MAPKKK cascade | 4.61E-13 | 1.70E-14 | 14223 | 84 | 1066 | 31 |
| Cell Signalling | Cell Signalling | cytokine-mediated signaling pathway | 1.17E-12 | 4.44E-14 | 14223 | 71 | 1066 | 28 |
| Cell Signalling | Cell Signalling | signal initiation by protein/peptide mediator | 1.72E-12 | 6.76E-14 | 14223 | 72 | 1066 | 28 |
| Cell Signalling | Cell Signalling | signal initiation by diffusible mediator | 1.72E-12 | 6.76E-14 | 14223 | 72 | 1066 | 28 |
| Cell Signalling | Cell Signalling | initiation of signal transduction | 1.72E-12 | 6.76E-14 | 14223 | 72 | 1066 | 28 |

| | | | | | | | | |
|---|---|---|---|---|---|---|---|---|
| Cell Signalling | Cell Signalling | positive regulation of protein kinase activity | 1.06E-11 | 4.46E-13 | 14223 | 239 | 1066 | 53 |
| Cell Signalling | Cell Signalling | regulation of MAPKKK cascade | 1.50E-11 | 6.44E-13 | 14223 | 163 | 1066 | 42 |
| Cell Signalling | Cell Signalling | positive regulation of kinase activity | 4.62E-11 | 2.09E-12 | 14223 | 248 | 1066 | 53 |
| Cell Signalling | Cell Signalling | response to estrogen stimulus | 1.31E-10 | 6.20E-12 | 14223 | 114 | 1066 | 33 |
| Cell Signalling | Cell Signalling | regulation of pathway-restricted SMAD protein phosphorylation | 2.39E-10 | 1.15E-11 | 14223 | 21 | 1066 | 14 |
| Cell Signalling | Cell Signalling | BMP signaling pathway | 2.79E-10 | 1.37E-11 | 14223 | 45 | 1066 | 20 |
| Cell Signalling | Cell Signalling | regulation of protein kinase activity | 1.65E-09 | 8.92E-11 | 14223 | 372 | 1066 | 65 |
| Cell Signalling | Cell Signalling | response to extracellular stimulus | 2.08E-09 | 1.13E-10 | 14223 | 266 | 1066 | 52 |
| Cell Signalling | Cell Signalling | signal transmission via phosphorylation event | 4.58E-09 | 2.62E-10 | 14223 | 364 | 1066 | 63 |
| Cell Signalling | Cell Signalling | intracellular protein kinase cascade | 4.58E-09 | 2.62E-10 | 14223 | 364 | 1066 | 63 |
| Cell Signalling | Cell Signalling | fibroblast growth factor receptor signaling pathway | 5.33E-09 | 3.06E-10 | 14223 | 33 | 1066 | 16 |
| Cell Signalling | Cell Signalling | response to insulin stimulus | 7.45E-09 | 4.34E-10 | 14223 | 125 | 1066 | 32 |
| Cell Signalling | Cell Signalling | response to corticosteroid stimulus | 8.82E-09 | 5.22E-10 | 14223 | 106 | 1066 | 29 |
| Cell Signalling | Cell Signalling | positive regulation of tyrosine phosphorylation of Stat5 protein | 1.13E-08 | 6.75E-10 | 14223 | 10 | 1066 | 9 |
| Cell Signalling | Cell Signalling | regulation of transmembrane receptor protein serine/threonine kinase signaling pathway | 2.27E-08 | 1.47E-09 | 14223 | 91 | 1066 | 26 |
| Cell Signalling | Cell Signalling | response to glucocorticoid stimulus | 3.31E-08 | 2.22E-09 | 14223 | 99 | 1066 | 27 |
| Cell Signalling | Cell Signalling | positive regulation of pathway-restricted SMAD protein phosphorylation | 4.72E-08 | 3.24E-09 | 14223 | 17 | 1066 | 11 |
| Cell Signalling | Cell Signalling | regulation of tyrosine phosphorylation of Stat3 protein | 4.72E-08 | 3.24E-09 | 14223 | 17 | 1066 | 11 |
| Cell Signalling | Cell Signalling | positive regulation of tyrosine phosphorylation of Stat3 protein | 5.01E-08 | 3.46E-09 | 14223 | 11 | 1066 | 9 |
| Cell Signalling | Cell Signalling | collagen catabolic process | 6.42E-08 | 4.61E-09 | 14223 | 21 | 1066 | 12 |
| Cell Signalling | Cell Signalling | regulation of tyrosine phosphorylation of Stat5 protein | 1.68E-07 | 1.29E-08 | 14223 | 12 | 1066 | 9 |
| Cell Signalling | Cell Signalling | JAK-STAT cascade | 1.92E-07 | 1.49E-08 | 14223 | 36 | 1066 | 15 |
| Cell Signalling | Cell Signalling | response to estradiol stimulus | 2.02E-07 | 1.58E-08 | 14223 | 63 | 1066 | 20 |
| Cell Signalling | Cell Signalling | positive regulation of MAP kinase activity | 2.64E-07 | 2.13E-08 | 14223 | 109 | 1066 | 27 |
| Cell Signalling | Cell Signalling | activation of protein kinase activity | 1.16E-06 | 1.00E-07 | 14223 | 124 | 1066 | 28 |
| Cell Signalling | Cell Signalling | pathway-restricted SMAD protein phosphorylation | 1.18E-06 | 1.02E-07 | 14223 | 14 | 1066 | 9 |
| Cell Signalling | Cell Signalling | intracellular signal transduction | 1.34E-06 | 1.17E-07 | 14223 | 833 | 1066 | 104 |
| Cell Signalling | Cell Signalling | positive regulation of organelle organization | 2.19E-06 | 1.96E-07 | 14223 | 92 | 1066 | 23 |
| Cell Signalling | Cell Signalling | regulation of MAP kinase activity | 3.15E-06 | 2.86E-07 | 14223 | 153 | 1066 | 31 |
| Cell Signalling | Cell Signalling | positive regulation of NF-kappaB transcription factor activity | 3.18E-06 | 2.92E-07 | 14223 | 55 | 1066 | 17 |
| Cell Signalling | Cell Signalling | positive regulation of protein kinase B signaling cascade | 4.31E-06 | 4.00E-07 | 14223 | 24 | 1066 | 11 |
| Cell Signalling | Cell Signalling | intracellular signaling pathway | 4.63E-06 | 4.33E-07 | 14223 | 1157 | 1066 | 132 |
| Cell Signalling | Cell Signalling | response to vitamin | 4.68E-06 | 4.39E-07 | 14223 | 103 | 1066 | 24 |
| Cell Signalling | Cell Signalling | response to cytokine stimulus | 9.34E-06 | 9.22E-07 | 14223 | 107 | 1066 | 24 |
| Cell Signalling | Cell Signalling | regulation of ERK1 and ERK2 cascade | 1.17E-05 | 1.17E-06 | 14223 | 42 | 1066 | 14 |
| Cell Signalling | Cell Signalling | regulation of protein kinase B signaling cascade | 2.31E-05 | 2.39E-06 | 14223 | 33 | 1066 | 12 |
| Cell Signalling | Cell Signalling | epidermal growth factor receptor signaling pathway | 2.47E-05 | 2.60E-06 | 14223 | 28 | 1066 | 11 |
| Cell Signalling | Cell Signalling | cellular response to insulin stimulus | 2.61E-05 | 2.76E-06 | 14223 | 77 | 1066 | 19 |
| Cell Signalling | Cell Signalling | cellular response to peptide hormone stimulus | 3.12E-05 | 3.36E-06 | 14223 | 85 | 1066 | 20 |
| Cell Signalling | Cell Signalling | positive regulation of ERK1 and ERK2 cascade | 3.17E-05 | 3.44E-06 | 14223 | 34 | 1066 | 12 |
| Cell Signalling | Cell Signalling | positive regulation of phosphoinositide 3-kinase cascade | 3.56E-05 | 3.88E-06 | 14223 | 15 | 1066 | 8 |
| Cell Signalling | Cell Signalling | regulation of phospholipase A2 activity | 3.88E-05 | 4.29E-06 | 14223 | 8 | 1066 | 6 |
| Cell Signalling | Cell Signalling | positive regulation of SMAD protein nuclear translocation | 3.88E-05 | 4.29E-06 | 14223 | 8 | 1066 | 6 |
| Cell Signalling | Cell Signalling | regulation of fibroblast proliferation | 6.29E-05 | 7.18E-06 | 14223 | 42 | 1066 | 13 |
| Cell Signalling | Cell Signalling | regulation of phosphoinositide 3-kinase cascade | 6.33E-05 | 7.25E-06 | 14223 | 16 | 1066 | 8 |
| Cell Signalling | Cell Signalling | response to ethanol | 7.92E-05 | 9.33E-06 | 14223 | 76 | 1066 | 18 |
| Cell Signalling | Cell Signalling | positive regulation of transmembrane receptor protein serine/threonine kinase signaling pathway | 7.93E-05 | 9.42E-06 | 14223 | 37 | 1066 | 12 |
| Cell Signalling | Cell Signalling | response to metal ion | 9.24E-05 | 1.10E-05 | 14223 | 155 | 1066 | 28 |
| Cell Signalling | Cell Signalling | regulation of SMAD protein nuclear translocation | 1.00E-04 | 1.21E-05 | 14223 | 9 | 1066 | 6 |
| Cell Signalling | Cell Signalling | negative regulation of signaling pathway | 1.01E-04 | 1.23E-05 | 14223 | 262 | 1066 | 40 |

| | | | | | | | | |
|---|---|---|---|---|---|---|---|---|
| Cell Signalling | Cell Signalling | insulin receptor signaling pathway | 1.05E-04 | 1.28E-05 | 14223 | 38 | 1066 | 12 |
| Cell Signalling | Cell Signalling | activation of phospholipase A2 activity | 1.07E-04 | 1.32E-05 | 14223 | 6 | 1066 | 5 |
| Cell Signalling | Cell Signalling | response to organic nitrogen | 1.32E-04 | 1.65E-05 | 14223 | 79 | 1066 | 18 |
| Cell Signalling | Cell Signalling | MAPKKK cascade | 1.76E-04 | 2.27E-05 | 14223 | 187 | 1066 | 31 |
| Cell Signalling | Cell Signalling | regulation of I-kappaB kinase/NF-kappaB cascade | 1.87E-04 | 2.43E-05 | 14223 | 128 | 1066 | 24 |
| Cell Signalling | Cell Signalling | positive regulation of phospholipase A2 activity | 3.14E-04 | 4.33E-05 | 14223 | 7 | 1066 | 5 |
| Cell Signalling | Cell Signalling | positive regulation of I-kappaB kinase/NF-kappaB cascade | 3.16E-04 | 4.36E-05 | 14223 | 116 | 1066 | 22 |
| Cell Signalling | Cell Signalling | response to tumor necrosis factor | 3.83E-04 | 5.41E-05 | 14223 | 20 | 1066 | 8 |
| Cell Signalling | Cell Signalling | response to mechanical stimulus | 7.22E-04 | 1.07E-04 | 14223 | 60 | 1066 | 14 |
| Cell Signalling | Cell Signalling | positive regulation of Rac GTPase activity | 7.25E-04 | 1.08E-04 | 14223 | 8 | 1066 | 5 |
| Cell Signalling | Cell Signalling | positive regulation of epidermal growth factor receptor signaling pathway | 7.26E-04 | 1.09E-04 | 14223 | 12 | 1066 | 6 |
| Cell Signalling | Cell Signalling | ciliary neurotrophic factor-mediated signaling pathway | 9.46E-04 | 1.48E-04 | 14223 | 5 | 1066 | 4 |
| Cell Signalling | Cell Signalling | regulation of stress-activated protein kinase signaling cascade | 1.02E-03 | 1.60E-04 | 14223 | 85 | 1066 | 17 |
| Cell Signalling | Cell Signalling | response to progesterone stimulus | 1.44E-03 | 2.41E-04 | 14223 | 24 | 1066 | 8 |
| Cell Signalling | Cell Signalling | platelet-derived growth factor receptor signaling pathway | 1.74E-03 | 2.94E-04 | 14223 | 19 | 1066 | 7 |
| Cell Signalling | Cell Signalling | positive regulation of Ras protein signal transduction | 1.74E-03 | 2.94E-04 | 14223 | 19 | 1066 | 7 |
| Cell Signalling | Cell Signalling | negative regulation of transmembrane receptor protein serine/threonine kinase signaling pathway | 2.16E-03 | 3.77E-04 | 14223 | 45 | 1066 | 11 |
| Cell Signalling | Cell Signalling | negative regulation of signaling process | 2.17E-03 | 3.78E-04 | 14223 | 125 | 1066 | 21 |
| Cell Signalling | Cell Signalling | SMAD protein nuclear translocation | 2.34E-03 | 4.16E-04 | 14223 | 6 | 1066 | 4 |
| Cell Signalling | Cell Signalling | positive regulation of epidermal growth factor receptor activity | 2.34E-03 | 4.16E-04 | 14223 | 6 | 1066 | 4 |
| Cell Signalling | Cell Signalling | vascular endothelial growth factor receptor signaling pathway | 2.34E-03 | 4.16E-04 | 14223 | 6 | 1066 | 4 |
| Cell Signalling | Cell Signalling | regulation of JNK cascade | 2.45E-03 | 4.44E-04 | 14223 | 76 | 1066 | 15 |
| Cell Signalling | Cell Signalling | negative regulation of intracellular protein kinase cascade | 2.89E-03 | 5.32E-04 | 14223 | 54 | 1066 | 12 |
| Cell Signalling | Cell Signalling | SMAD protein signal transduction | 3.84E-03 | 7.27E-04 | 14223 | 16 | 1066 | 6 |
| Cell Signalling | Cell Signalling | regulation of vascular endothelial growth factor production | 3.84E-03 | 7.27E-04 | 14223 | 16 | 1066 | 6 |
| Cell Signalling | Cell Signalling | regulation of transforming growth factor-beta production | 3.86E-03 | 7.38E-04 | 14223 | 11 | 1066 | 5 |
| Cell Signalling | Cell Signalling | regulation of platelet activation | 3.86E-03 | 7.38E-04 | 14223 | 11 | 1066 | 5 |
| Cell Signalling | Cell Signalling | positive regulation of stress-activated protein kinase signaling cascade | 4.04E-03 | 7.77E-04 | 14223 | 28 | 1066 | 8 |
| Cell Signalling | Cell Signalling | negative regulation of signal transduction | 4.11E-03 | 7.93E-04 | 14223 | 123 | 1066 | 20 |
| Cell Signalling | Cell Signalling | positive regulation of JNK cascade | 4.20E-03 | 8.16E-04 | 14223 | 22 | 1066 | 7 |
| Cell Signalling | Cell Signalling | integrin-mediated signaling pathway | 4.56E-03 | 8.91E-04 | 14223 | 57 | 1066 | 12 |
| Cell Signalling | Cell Signalling | positive regulation of protein tyrosine kinase activity | 4.62E-03 | 9.14E-04 | 14223 | 7 | 1066 | 4 |
| Cell Signalling | Cell Signalling | response to vitamin D | 5.22E-03 | 1.05E-03 | 14223 | 17 | 1066 | 6 |
| Cell Signalling | Cell Signalling | response to lipid | 5.27E-03 | 1.07E-03 | 14223 | 36 | 1066 | 9 |
| Cell Signalling | Cell Signalling | positive regulation of vascular endothelial growth factor receptor signaling pathway | 5.77E-03 | 1.19E-03 | 14223 | 12 | 1066 | 5 |
| Cell Signalling | Cell Signalling | regulation of epidermal growth factor receptor signaling pathway | 6.91E-03 | 1.45E-03 | 14223 | 24 | 1066 | 7 |
| Cell Signalling | Cell Signalling | regulation of transforming growth factor-beta2 production | 7.32E-03 | 1.59E-03 | 14223 | 4 | 1066 | 3 |
| Cell Signalling | Cell Signalling | regulation of follicle-stimulating hormone secretion | 7.32E-03 | 1.59E-03 | 14223 | 4 | 1066 | 3 |
| Cell Signalling | Cell Signalling | response to caffeine | 7.74E-03 | 1.72E-03 | 14223 | 8 | 1066 | 4 |
| Cell Signalling | Cell Signalling | positive regulation of insulin-like growth factor receptor signaling pathway | 7.74E-03 | 1.72E-03 | 14223 | 8 | 1066 | 4 |
| Cell Signalling | Cell Signalling | activation of MAPK activity | 8.07E-03 | 1.79E-03 | 14223 | 78 | 1066 | 14 |
| Cell Signalling | Cell Signalling | positive regulation of NF-kappaB import into nucleus | 8.11E-03 | 1.81E-03 | 14223 | 13 | 1066 | 5 |
| Cell Signalling | Cell Signalling | activation of NF-kappaB-inducing kinase activity | 8.11E-03 | 1.81E-03 | 14223 | 13 | 1066 | 5 |
| Cell Signalling | Cell Signalling | response to amine stimulus | 8.13E-03 | 1.82E-03 | 14223 | 46 | 1066 | 10 |
| Cell Signalling | Cell Signalling | regulation of insulin-like growth factor receptor signaling pathway | 1.15E-02 | 2.65E-03 | 14223 | 14 | 1066 | 5 |
| Cell Signalling | Cell Signalling | regulation of receptor activity | 1.47E-02 | 3.56E-03 | 14223 | 21 | 1066 | 6 |
| Cell Signalling | Cell Signalling | regulation of vascular endothelial growth factor receptor signaling pathway | 1.96E-02 | 5.09E-03 | 14223 | 16 | 1066 | 5 |
| Cell Signalling | Cell Signalling | regulation of epidermal growth factor receptor activity | 1.96E-02 | 5.09E-03 | 14223 | 16 | 1066 | 5 |
| Cell Signalling | Cell Signalling | response to purine | 2.41E-02 | 6.75E-03 | 14223 | 11 | 1066 | 4 |
| Cell Signalling | Cell Signalling | regulation of protein tyrosine kinase activity | 2.41E-02 | 6.77E-03 | 14223 | 17 | 1066 | 5 |

| | | | | | | | | |
|---|---|---|---|---|---|---|---|---|
| Cell Signalling | Cell Signalling | positive regulation of Rho GTPase activity | 2.41E-02 | 6.77E-03 | 14223 | 17 | 1066 | 5 |
| Cell Signalling | Cell Signalling | regulation of NF-kappaB import into nucleus | 2.54E-02 | 7.28E-03 | 14223 | 24 | 1066 | 6 |
| Cell Signalling | Cell Signalling | regulation of phospholipase activity | 2.68E-02 | 7.68E-03 | 14223 | 73 | 1066 | 12 |
| Cell Signalling | Cell Signalling | response to alkaloid | 2.80E-02 | 8.05E-03 | 14223 | 56 | 1066 | 10 |
| Cell Signalling | Cell Signalling | I-kappaB kinase/NF-kappaB cascade | 1.04E-01 | 4.05E-02 | 14223 | 62 | 1066 | 9 |
| Cell Signalling | Cell Signalling | negative regulation of hydrolase activity | 1.48E-01 | 7.20E-02 | 14223 | 59 | 1066 | 8 |
| Cell Signalling | Cell Signalling | regulation of Rho protein signal transduction | 6.05E-01 | 5.34E-01 | 14223 | 105 | 1066 | 8 |
| Cell Signalling | Cell Signalling | regulation of small GTPase mediated signal transduction | 7.17E-01 | 6.60E-01 | 14223 | 272 | 1066 | 19 |
| Cell Signalling | Cell Signalling | regulation of GTPase activity | 7.71E-01 | 7.23E-01 | 14223 | 139 | 1066 | 9 |
| Cell Signalling | Cell Signalling | regulation of Ras protein signal transduction | 8.13E-01 | 7.72E-01 | 14223 | 234 | 1066 | 15 |
| Cell Survival | Cell Survival | regulation of cell death | 3.56E-26 | 4.34E-28 | 14223 | 875 | 1066 | 161 |
| Cell Survival | Cell Survival | regulation of programmed cell death | 4.28E-26 | 5.31E-28 | 14223 | 868 | 1066 | 160 |
| Cell Survival | Cell Survival | regulation of apoptosis | 1.36E-25 | 1.77E-27 | 14223 | 860 | 1066 | 158 |
| Cell Survival | Cell Survival | negative regulation of cell death | 8.89E-22 | 1.80E-23 | 14223 | 395 | 1066 | 92 |
| Cell Survival | Cell Survival | negative regulation of programmed cell death | 1.16E-20 | 2.51E-22 | 14223 | 387 | 1066 | 89 |
| Cell Survival | Cell Survival | negative regulation of apoptosis | 1.75E-20 | 3.87E-22 | 14223 | 382 | 1066 | 88 |
| Cell Survival | Cell Survival | anti-apoptosis | 1.06E-14 | 3.20E-16 | 14223 | 203 | 1066 | 53 |
| Cell Survival | Cell Survival | positive regulation of cell death | 4.62E-11 | 2.09E-12 | 14223 | 452 | 1066 | 78 |
| Cell Survival | Cell Survival | positive regulation of apoptosis | 5.54E-11 | 2.54E-12 | 14223 | 445 | 1066 | 77 |
| Cell Survival | Cell Survival | positive regulation of programmed cell death | 7.68E-11 | 3.60E-12 | 14223 | 448 | 1066 | 77 |
| Cell Survival | Cell Survival | cell death | 2.11E-07 | 1.67E-08 | 14223 | 701 | 1066 | 94 |
| Cell Survival | Cell Survival | programmed cell death | 6.78E-07 | 5.68E-08 | 14223 | 579 | 1066 | 80 |
| Cell Survival | Cell Survival | apoptosis | 1.34E-06 | 1.17E-07 | 14223 | 569 | 1066 | 78 |
| Cell Survival | Cell Survival | activation of pro-apoptotic gene products | 2.38E-05 | 2.49E-06 | 14223 | 23 | 1066 | 10 |
| Cell Survival | Cell Survival | regulation of muscle cell apoptosis | 3.06E-05 | 3.29E-06 | 14223 | 11 | 1066 | 7 |
| Cell Survival | Cell Survival | positive regulation of anti-apoptosis | 1.32E-04 | 1.65E-05 | 14223 | 33 | 1066 | 11 |
| Cell Survival | Cell Survival | induction of apoptosis | 1.60E-04 | 2.04E-05 | 14223 | 315 | 1066 | 45 |
| Cell Survival | Cell Survival | induction of programmed cell death | 1.72E-04 | 2.22E-05 | 14223 | 316 | 1066 | 45 |
| Cell Survival | Cell Survival | regulation of survival gene product expression | 5.54E-04 | 8.16E-05 | 14223 | 21 | 1066 | 8 |
| Cell Survival | Cell Survival | regulation of anti-apoptosis | 7.97E-04 | 1.21E-04 | 14223 | 40 | 1066 | 11 |
| Cell Survival | Cell Survival | negative regulation of muscle cell apoptosis | 4.62E-03 | 9.14E-04 | 14223 | 7 | 1066 | 4 |
| Cell Survival | Cell Survival | regulation of B cell apoptosis | 4.62E-03 | 9.14E-04 | 14223 | 7 | 1066 | 4 |
| Cell Survival | Cell Survival | regulation of smooth muscle cell apoptosis | 4.62E-03 | 9.14E-04 | 14223 | 7 | 1066 | 4 |
| Cell Survival | Cell Survival | regulation of retinal cell programmed cell death | 4.62E-03 | 9.14E-04 | 14223 | 7 | 1066 | 4 |
| Cell Survival | Cell Survival | positive regulation of caspase activity | 6.81E-03 | 1.43E-03 | 14223 | 68 | 1066 | 13 |
| Cell Survival | Cell Survival | positive regulation of smooth muscle cell apoptosis | 7.32E-03 | 1.59E-03 | 14223 | 4 | 1066 | 3 |
| Cell Survival | Cell Survival | negative regulation of striated muscle cell apoptosis | 7.32E-03 | 1.59E-03 | 14223 | 4 | 1066 | 3 |
| Cell Survival | Cell Survival | regulation of striated muscle cell apoptosis | 7.32E-03 | 1.59E-03 | 14223 | 4 | 1066 | 3 |
| Cell Survival | Cell Survival | positive regulation of muscle cell apoptosis | 7.32E-03 | 1.59E-03 | 14223 | 4 | 1066 | 3 |
| Cell Survival | Cell Survival | regulation of lymphocyte apoptosis | 8.11E-03 | 1.81E-03 | 14223 | 13 | 1066 | 5 |
| Cell Survival | Cell Survival | cytolysis | 8.94E-03 | 2.03E-03 | 14223 | 19 | 1066 | 6 |
| Cell Survival | Cell Survival | regulation of caspase activity | 5.63E-02 | 1.94E-02 | 14223 | 92 | 1066 | 13 |
| Cell Survival | Cell Survival | localization | 3.62E-01 | 2.68E-01 | 14223 | 2984 | 1066 | 232 |
| Immunomodulation | Immunomodulation | immune system process | 5.42E-64 | 3.42E-67 | 14223 | 951 | 1066 | 236 |
| Immunomodulation | Immunomodulation | inflammatory response | 3.10E-56 | 3.26E-59 | 14223 | 322 | 1066 | 126 |
| Immunomodulation | Immunomodulation | defense response | 1.90E-46 | 6.38E-49 | 14223 | 628 | 1066 | 164 |
| Immunomodulation | Immunomodulation | immune response | 3.50E-43 | 1.40E-45 | 14223 | 620 | 1066 | 158 |
| Immunomodulation | Immunomodulation | regulation of immune system process | 2.53E-31 | 2.02E-33 | 14223 | 427 | 1066 | 112 |
| Immunomodulation | Immunomodulation | positive regulation of immune system process | 4.41E-22 | 8.61E-24 | 14223 | 268 | 1066 | 74 |
| Immunomodulation | Immunomodulation | regulation of immune response | 1.99E-18 | 4.63E-20 | 14223 | 238 | 1066 | 64 |

| | | | | | | | | |
|---|---|---|---|---|---|---|---|---|
| Immunomodulation | Immunomodulation | regulation of cytokine production | 3.37E-18 | 8.06E-20 | 14223 | 208 | 1066 | 59 |
| Immunomodulation | Immunomodulation | response to lipopolysaccharide | 6.71E-17 | 1.75E-18 | 14223 | 118 | 1066 | 42 |
| Immunomodulation | Immunomodulation | response to molecule of bacterial origin | 4.21E-16 | 1.17E-17 | 14223 | 129 | 1066 | 43 |
| Immunomodulation | Immunomodulation | response to bacterium | 1.36E-15 | 3.85E-17 | 14223 | 234 | 1066 | 59 |
| Immunomodulation | Immunomodulation | regulation of lymphocyte activation | 2.79E-15 | 8.03E-17 | 14223 | 159 | 1066 | 47 |
| Immunomodulation | Immunomodulation | regulation of leukocyte activation | 6.18E-15 | 1.83E-16 | 14223 | 181 | 1066 | 50 |
| Immunomodulation | Immunomodulation | leukocyte activation | 8.47E-15 | 2.54E-16 | 14223 | 236 | 1066 | 58 |
| Immunomodulation | Immunomodulation | acute inflammatory response | 2.62E-14 | 8.63E-16 | 14223 | 92 | 1066 | 34 |
| Immunomodulation | Immunomodulation | regulation of secretion | 7.84E-13 | 2.95E-14 | 14223 | 246 | 1066 | 56 |
| Immunomodulation | Immunomodulation | positive regulation of immune response | 1.02E-11 | 4.27E-13 | 14223 | 148 | 1066 | 40 |
| Immunomodulation | Immunomodulation | regulation of inflammatory response | 4.25E-11 | 1.90E-12 | 14223 | 92 | 1066 | 30 |
| Immunomodulation | Immunomodulation | negative regulation of immune system process | 2.39E-10 | 1.15E-11 | 14223 | 92 | 1066 | 29 |
| Immunomodulation | Immunomodulation | immune effector process | 4.51E-10 | 2.26E-11 | 14223 | 132 | 1066 | 35 |
| Immunomodulation | Immunomodulation | positive regulation of lymphocyte activation | 5.24E-10 | 2.64E-11 | 14223 | 107 | 1066 | 31 |
| Immunomodulation | Immunomodulation | lymphocyte activation | 6.00E-10 | 3.03E-11 | 14223 | 189 | 1066 | 43 |
| Immunomodulation | Immunomodulation | regulation of defense response | 9.82E-10 | 5.03E-11 | 14223 | 163 | 1066 | 39 |
| Immunomodulation | Immunomodulation | regulation of B cell activation | 1.16E-09 | 6.06E-11 | 14223 | 53 | 1066 | 21 |
| Immunomodulation | Immunomodulation | positive regulation of leukocyte activation | 1.61E-09 | 8.60E-11 | 14223 | 118 | 1066 | 32 |
| Immunomodulation | Immunomodulation | innate immune response | 2.02E-09 | 1.10E-10 | 14223 | 167 | 1066 | 39 |
| Immunomodulation | Immunomodulation | adaptive immune response | 4.53E-09 | 2.58E-10 | 14223 | 73 | 1066 | 24 |
| Immunomodulation | Immunomodulation | regulation of T cell activation | 1.31E-08 | 7.95E-10 | 14223 | 121 | 1066 | 31 |
| Immunomodulation | Immunomodulation | adaptive immune response based on somatic recombination of immune receptors built from immunoglobulin superfamily domains | 1.85E-08 | 1.16E-09 | 14223 | 72 | 1066 | 23 |
| Immunomodulation | Immunomodulation | positive regulation of defense response | 1.88E-08 | 1.18E-09 | 14223 | 84 | 1066 | 25 |
| Immunomodulation | Immunomodulation | negative regulation of leukocyte activation | 3.28E-08 | 2.19E-09 | 14223 | 57 | 1066 | 20 |
| Immunomodulation | Immunomodulation | negative regulation of lymphocyte activation | 3.72E-08 | 2.51E-09 | 14223 | 52 | 1066 | 19 |
| Immunomodulation | Immunomodulation | positive regulation of inflammatory response | 4.06E-08 | 2.77E-09 | 14223 | 42 | 1066 | 17 |
| Immunomodulation | Immunomodulation | activation of plasma proteins involved in acute inflammatory response | 4.06E-08 | 2.77E-09 | 14223 | 42 | 1066 | 17 |
| Immunomodulation | Immunomodulation | regulation of immunoglobulin production | 6.74E-08 | 4.87E-09 | 14223 | 25 | 1066 | 13 |
| Immunomodulation | Immunomodulation | positive regulation of B cell activation | 8.77E-08 | 6.44E-09 | 14223 | 39 | 1066 | 16 |
| Immunomodulation | Immunomodulation | regulation of adaptive immune response | 1.63E-07 | 1.25E-08 | 14223 | 51 | 1066 | 18 |
| Immunomodulation | Immunomodulation | complement activation | 1.96E-07 | 1.52E-08 | 14223 | 41 | 1066 | 16 |
| Immunomodulation | Immunomodulation | leukocyte mediated immunity | 4.82E-07 | 4.01E-08 | 14223 | 85 | 1066 | 23 |
| Immunomodulation | Immunomodulation | regulation of adaptive immune response based on somatic recombination of immune receptors built from immunoglobulin superfamily domains | 7.12E-07 | 6.02E-08 | 14223 | 50 | 1066 | 17 |
| Immunomodulation | Immunomodulation | T cell activation | 2.42E-06 | 2.18E-07 | 14223 | 121 | 1066 | 27 |
| Immunomodulation | Immunomodulation | regulation of isotype switching | 3.95E-06 | 3.66E-07 | 14223 | 12 | 1066 | 8 |
| Immunomodulation | Immunomodulation | negative regulation of immune response | 4.93E-06 | 4.63E-07 | 14223 | 29 | 1066 | 12 |
| Immunomodulation | Immunomodulation | activation of immune response | 5.77E-06 | 5.45E-07 | 14223 | 97 | 1066 | 23 |
| Immunomodulation | Immunomodulation | lymphocyte mediated immunity | 8.36E-06 | 8.17E-07 | 14223 | 65 | 1066 | 18 |
| Immunomodulation | Immunomodulation | regulation of production of molecular mediator of immune response | 8.56E-06 | 8.37E-07 | 14223 | 41 | 1066 | 14 |
| Immunomodulation | Immunomodulation | regulation of interleukin-6 production | 2.14E-05 | 2.21E-06 | 14223 | 44 | 1066 | 14 |
| Immunomodulation | Immunomodulation | regulation of interferon-gamma production | 2.31E-05 | 2.39E-06 | 14223 | 33 | 1066 | 12 |
| Immunomodulation | Immunomodulation | regulation of immune effector process | 2.68E-05 | 2.84E-06 | 14223 | 106 | 1066 | 23 |
| Immunomodulation | Immunomodulation | regulation of MHC class II biosynthetic process | 3.06E-05 | 3.29E-06 | 14223 | 11 | 1066 | 7 |
| Immunomodulation | Immunomodulation | positive regulation of interleukin-12 production | 3.06E-05 | 3.29E-06 | 14223 | 11 | 1066 | 7 |
| Immunomodulation | Immunomodulation | humoral immune response | 3.14E-05 | 3.39E-06 | 14223 | 78 | 1066 | 19 |
| Immunomodulation | Immunomodulation | immunoglobulin mediated immune response | 3.52E-05 | 3.84E-06 | 14223 | 52 | 1066 | 15 |
| Immunomodulation | Immunomodulation | positive regulation of isotype switching | 3.88E-05 | 4.29E-06 | 14223 | 8 | 1066 | 6 |
| Immunomodulation | Immunomodulation | positive regulation of MHC class II biosynthetic process | 3.88E-05 | 4.29E-06 | 14223 | 8 | 1066 | 6 |
| Immunomodulation | Immunomodulation | B cell mediated immunity | 4.48E-05 | 4.98E-06 | 14223 | 53 | 1066 | 15 |
| Immunomodulation | Immunomodulation | negative regulation of T cell activation | 6.29E-05 | 7.18E-06 | 14223 | 42 | 1066 | 13 |

| | | | | | | | | |
|---|---|---|---|---|---|---|---|---|
| Immunomodulation | Immunomodulation | myeloid leukocyte activation | 7.79E-05 | 9.10E-06 | 14223 | 49 | 1066 | 14 |
| Immunomodulation | Immunomodulation | positive regulation of T cell activation | 9.50E-05 | 1.13E-05 | 14223 | 77 | 1066 | 18 |
| Immunomodulation | Immunomodulation | regulation of acute inflammatory response | 1.13E-04 | 1.39E-05 | 14223 | 27 | 1066 | 10 |
| Immunomodulation | Immunomodulation | regulation of interleukin-12 production | 1.18E-04 | 1.47E-05 | 14223 | 22 | 1066 | 9 |
| Immunomodulation | Immunomodulation | defense response to Gram-positive bacterium | 1.58E-04 | 2.02E-05 | 14223 | 28 | 1066 | 10 |
| Immunomodulation | Immunomodulation | regulation of chemokine production | 1.75E-04 | 2.25E-05 | 14223 | 23 | 1066 | 9 |
| Immunomodulation | Immunomodulation | regulation of tumor necrosis factor production | 1.76E-04 | 2.28E-05 | 14223 | 34 | 1066 | 11 |
| Immunomodulation | Immunomodulation | defense response to bacterium | 1.89E-04 | 2.47E-05 | 14223 | 120 | 1066 | 23 |
| Immunomodulation | Immunomodulation | regulation of immunoglobulin secretion | 2.13E-04 | 2.82E-05 | 14223 | 10 | 1066 | 6 |
| Immunomodulation | Immunomodulation | regulation of isotype switching to IgG isotypes | 3.14E-04 | 4.33E-05 | 14223 | 7 | 1066 | 5 |
| Immunomodulation | Immunomodulation | regulation of interleukin-6 biosynthetic process | 3.52E-04 | 4.91E-05 | 14223 | 15 | 1066 | 7 |
| Immunomodulation | Immunomodulation | positive regulation of interferon-gamma production | 3.52E-04 | 4.91E-05 | 14223 | 15 | 1066 | 7 |
| Immunomodulation | Immunomodulation | cellular response to molecule of bacterial origin | 3.83E-04 | 5.41E-05 | 14223 | 20 | 1066 | 8 |
| Immunomodulation | Immunomodulation | regulation of lymphocyte mediated immunity | 4.00E-04 | 5.68E-05 | 14223 | 50 | 1066 | 13 |
| Immunomodulation | Immunomodulation | response to virus | 4.45E-04 | 6.33E-05 | 14223 | 144 | 1066 | 25 |
| Immunomodulation | Immunomodulation | regulation of interleukin-2 production | 5.21E-04 | 7.52E-05 | 14223 | 32 | 1066 | 10 |
| Immunomodulation | Immunomodulation | regulation of immunoglobulin mediated immune response | 5.54E-04 | 8.16E-05 | 14223 | 21 | 1066 | 8 |
| Immunomodulation | Immunomodulation | regulation of B cell mediated immunity | 5.54E-04 | 8.16E-05 | 14223 | 21 | 1066 | 8 |
| Immunomodulation | Immunomodulation | cytokine production | 5.65E-04 | 8.35E-05 | 14223 | 45 | 1066 | 12 |
| Immunomodulation | Immunomodulation | B cell activation | 7.43E-04 | 1.12E-04 | 14223 | 75 | 1066 | 16 |
| Immunomodulation | Immunomodulation | positive regulation of acute inflammatory response | 8.49E-04 | 1.30E-04 | 14223 | 17 | 1066 | 7 |
| Immunomodulation | Immunomodulation | cellular defense response | 8.49E-04 | 1.30E-04 | 14223 | 61 | 1066 | 14 |
| Immunomodulation | Immunomodulation | response to interleukin-1 | 8.74E-04 | 1.35E-04 | 14223 | 28 | 1066 | 9 |
| Immunomodulation | Immunomodulation | positive regulation of isotype switching to IgG isotypes | 9.46E-04 | 1.48E-04 | 14223 | 5 | 1066 | 4 |
| Immunomodulation | Immunomodulation | regulation of immune response to tumor cell | 1.37E-03 | 2.29E-04 | 14223 | 9 | 1066 | 5 |
| Immunomodulation | Immunomodulation | positive regulation of response to tumor cell | 1.37E-03 | 2.29E-04 | 14223 | 9 | 1066 | 5 |
| Immunomodulation | Immunomodulation | positive regulation of immune response to tumor cell | 1.37E-03 | 2.29E-04 | 14223 | 9 | 1066 | 5 |
| Immunomodulation | Immunomodulation | regulation of response to tumor cell | 1.37E-03 | 2.29E-04 | 14223 | 9 | 1066 | 5 |
| Immunomodulation | Immunomodulation | regulation of leukocyte mediated immunity | 2.00E-03 | 3.45E-04 | 14223 | 59 | 1066 | 13 |
| Immunomodulation | Immunomodulation | regulation of cytokine secretion | 2.09E-03 | 3.65E-04 | 14223 | 38 | 1066 | 10 |
| Immunomodulation | Immunomodulation | type I interferon biosynthetic process | 2.34E-03 | 4.20E-04 | 14223 | 3 | 1066 | 3 |
| Immunomodulation | Immunomodulation | type I interferon production | 2.34E-03 | 4.20E-04 | 14223 | 3 | 1066 | 3 |
| Immunomodulation | Immunomodulation | positive regulation of interleukin-6 biosynthetic process | 2.34E-03 | 4.16E-04 | 14223 | 6 | 1066 | 4 |
| Immunomodulation | Immunomodulation | cellular response to lipoteichoic acid | 2.34E-03 | 4.20E-04 | 14223 | 3 | 1066 | 3 |
| Immunomodulation | Immunomodulation | inflammatory response to antigenic stimulus | 2.34E-03 | 4.16E-04 | 14223 | 6 | 1066 | 4 |
| Immunomodulation | Immunomodulation | T cell activation via T cell receptor contact with antigen bound to MHC molecule on antigen presenting cell | 2.34E-03 | 4.20E-04 | 14223 | 3 | 1066 | 3 |
| Immunomodulation | Immunomodulation | regulation of interleukin-2 biosynthetic process | 2.36E-03 | 4.24E-04 | 14223 | 20 | 1066 | 7 |
| Immunomodulation | Immunomodulation | cytokine biosynthetic process | 2.64E-03 | 4.85E-04 | 14223 | 15 | 1066 | 6 |
| Immunomodulation | Immunomodulation | negative regulation of B cell activation | 2.64E-03 | 4.85E-04 | 14223 | 15 | 1066 | 6 |
| Immunomodulation | Immunomodulation | leukocyte homeostasis | 2.91E-03 | 5.37E-04 | 14223 | 33 | 1066 | 9 |
| Immunomodulation | Immunomodulation | defense response to Gram-negative bacterium | 3.86E-03 | 7.38E-04 | 14223 | 11 | 1066 | 5 |
| Immunomodulation | Immunomodulation | positive regulation of innate immune response | 4.21E-03 | 8.21E-04 | 14223 | 49 | 1066 | 11 |
| Immunomodulation | Immunomodulation | complement activation, lectin pathway | 4.62E-03 | 9.14E-04 | 14223 | 7 | 1066 | 4 |
| Immunomodulation | Immunomodulation | complement activation, classical pathway | 5.04E-03 | 1.00E-03 | 14223 | 29 | 1066 | 8 |
| Immunomodulation | Immunomodulation | cellular response to lipopolysaccharide | 5.22E-03 | 1.05E-03 | 14223 | 17 | 1066 | 6 |
| Immunomodulation | Immunomodulation | somatic diversification of immune receptors via germline recombination within a single locus | 5.37E-03 | 1.10E-03 | 14223 | 23 | 1066 | 7 |
| Immunomodulation | Immunomodulation | positive regulation of interleukin-6 production | 5.37E-03 | 1.10E-03 | 14223 | 23 | 1066 | 7 |
| Immunomodulation | Immunomodulation | positive regulation of cytokine secretion | 6.17E-03 | 1.28E-03 | 14223 | 30 | 1066 | 8 |
| Immunomodulation | Immunomodulation | macrophage activation | 7.02E-03 | 1.48E-03 | 14223 | 18 | 1066 | 6 |
| Immunomodulation | Immunomodulation | negative regulation of cytokine production | 7.23E-03 | 1.52E-03 | 14223 | 45 | 1066 | 10 |

| | | | | | | | | |
|---|---|---|---|---|---|---|---|---|
| Immunomodulation | Immunomodulation | humoral immune response mediated by circulating immunoglobulin | 7.43E-03 | 1.61E-03 | 14223 | 31 | 1066 | 8 |
| Immunomodulation | Immunomodulation | positive regulation of chemokine biosynthetic process | 7.74E-03 | 1.72E-03 | 14223 | 8 | 1066 | 4 |
| Immunomodulation | Immunomodulation | positive regulation of prostaglandin secretion | 7.74E-03 | 1.72E-03 | 14223 | 8 | 1066 | 4 |
| Immunomodulation | Immunomodulation | regulation of prostaglandin secretion | 7.74E-03 | 1.72E-03 | 14223 | 8 | 1066 | 4 |
| Immunomodulation | Immunomodulation | positive regulation of icosanoid secretion | 7.74E-03 | 1.72E-03 | 14223 | 8 | 1066 | 4 |
| Immunomodulation | Immunomodulation | regulation of icosanoid secretion | 7.74E-03 | 1.72E-03 | 14223 | 8 | 1066 | 4 |
| Immunomodulation | Immunomodulation | positive regulation of tumor necrosis factor production | 8.11E-03 | 1.81E-03 | 14223 | 13 | 1066 | 5 |
| Immunomodulation | Immunomodulation | somatic diversification of immune receptors | 8.39E-03 | 1.89E-03 | 14223 | 25 | 1066 | 7 |
| Immunomodulation | Immunomodulation | response to lipoteichoic acid | 1.51E-02 | 3.74E-03 | 14223 | 5 | 1066 | 3 |
| Immunomodulation | Immunomodulation | regulation of innate immune response | 1.79E-02 | 4.54E-03 | 14223 | 60 | 1066 | 11 |
| Immunomodulation | Immunomodulation | T cell activation involved in immune response | 6.28E-02 | 2.20E-02 | 14223 | 15 | 1066 | 4 |
| Immunomodulation | Immunomodulation | cell activation involved in immune response | 7.65E-02 | 2.75E-02 | 14223 | 40 | 1066 | 7 |
| Immunomodulation | Immunomodulation | leukocyte activation involved in immune response | 7.65E-02 | 2.75E-02 | 14223 | 40 | 1066 | 7 |
| Immunomodulation | Immunomodulation | lymphocyte activation involved in immune response | 1.37E-01 | 5.80E-02 | 14223 | 20 | 1066 | 4 |
| Molecular Function | Molecular Function | positive regulation of molecular function | 2.41E-21 | 5.12E-23 | 14223 | 642 | 1066 | 123 |
| Molecular Function | Molecular Function | regulation of molecular function | 8.28E-16 | 2.31E-17 | 14223 | 1064 | 1066 | 157 |
| Molecular Function | Molecular Function | protein maturation | 4.48E-09 | 2.53E-10 | 14223 | 116 | 1066 | 31 |
| Molecular Function | Molecular Function | positive regulation of DNA binding | 3.65E-08 | 2.46E-09 | 14223 | 93 | 1066 | 26 |
| Molecular Function | Molecular Function | protein oligomerization | 5.96E-06 | 5.65E-07 | 14223 | 190 | 1066 | 35 |
| Molecular Function | Molecular Function | negative regulation of protein modification process | 1.04E-05 | 1.04E-06 | 14223 | 138 | 1066 | 28 |
| Molecular Function | Molecular Function | protein complex biogenesis | 1.36E-05 | 1.38E-06 | 14223 | 504 | 1066 | 68 |
| Molecular Function | Molecular Function | protein complex assembly | 1.36E-05 | 1.38E-06 | 14223 | 504 | 1066 | 68 |
| Molecular Function | Molecular Function | regulation of actin filament length | 2.35E-05 | 2.44E-06 | 14223 | 63 | 1066 | 17 |
| Molecular Function | Molecular Function | protein heterooligomerization | 2.58E-05 | 2.73E-06 | 14223 | 57 | 1066 | 16 |
| Molecular Function | Molecular Function | regulation of actin polymerization or depolymerization | 6.29E-05 | 7.18E-06 | 14223 | 61 | 1066 | 16 |
| Molecular Function | Molecular Function | negative regulation of molecular function | 1.91E-04 | 2.49E-05 | 14223 | 376 | 1066 | 51 |
| Molecular Function | Molecular Function | regulation of actin filament polymerization | 2.83E-04 | 3.84E-05 | 14223 | 55 | 1066 | 14 |
| Molecular Function | Molecular Function | peptidyl-tyrosine modification | 2.90E-04 | 3.96E-05 | 14223 | 42 | 1066 | 12 |
| Molecular Function | Molecular Function | macromolecular complex subunit organization | 3.77E-04 | 5.28E-05 | 14223 | 722 | 1066 | 83 |
| Molecular Function | Molecular Function | protein modification process | 1.05E-03 | 1.66E-04 | 14223 | 1526 | 1066 | 151 |
| Molecular Function | Molecular Function | positive regulation of actin filament polymerization | 1.17E-03 | 1.89E-04 | 14223 | 13 | 1066 | 6 |
| Molecular Function | Molecular Function | macromolecular complex assembly | 1.18E-03 | 1.92E-04 | 14223 | 673 | 1066 | 76 |
| Molecular Function | Molecular Function | DNA topological change | 1.37E-03 | 2.29E-04 | 14223 | 9 | 1066 | 5 |
| Molecular Function | Molecular Function | regulation of protein polymerization | 1.84E-03 | 3.15E-04 | 14223 | 66 | 1066 | 14 |
| Molecular Function | Molecular Function | peptidyl-amino acid modification | 1.86E-03 | 3.19E-04 | 14223 | 159 | 1066 | 25 |
| Molecular Function | Molecular Function | translational elongation | 5.63E-03 | 1.15E-03 | 14223 | 100 | 1066 | 17 |
| Molecular Function | Molecular Function | macromolecule modification | 5.79E-03 | 1.19E-03 | 14223 | 1607 | 1066 | 152 |
| Molecular Function | Molecular Function | regulation of protein complex assembly | 7.32E-03 | 1.57E-03 | 14223 | 94 | 1066 | 16 |
| Molecular Function | Molecular Function | positive regulation of protein polymerization | 1.47E-02 | 3.56E-03 | 14223 | 21 | 1066 | 6 |
| Molecular Function | Molecular Function | cellular protein complex assembly | 1.82E-02 | 4.67E-03 | 14223 | 152 | 1066 | 21 |
| Molecular Function | Molecular Function | positive regulation of protein complex assembly | 2.90E-02 | 8.34E-03 | 14223 | 32 | 1066 | 7 |
| Molecular Function | Molecular Function | cellular macromolecular complex subunit organization | 3.17E-01 | 2.20E-01 | 14223 | 357 | 1066 | 31 |
| Molecular Function | Molecular Function | DNA conformation change | 5.47E-01 | 4.71E-01 | 14223 | 139 | 1066 | 11 |
| Molecular Function | Molecular Function | macromolecule localization | 6.08E-01 | 5.41E-01 | 14223 | 1114 | 1066 | 83 |
| Organism Function | Organism Function | positive regulation of biological process | 7.42E-65 | 3.12E-68 | 14223 | 2214 | 1066 | 388 |
| Organism Function | Organism Function | regulation of multicellular organismal process | 3.96E-51 | 6.66E-54 | 14223 | 1075 | 1066 | 232 |
| Organism Function | Organism Function | multicellular organismal process | 4.06E-37 | 2.39E-39 | 14223 | 4378 | 1066 | 525 |
| Organism Function | Organism Function | regulation of biological quality | 6.48E-36 | 3.94E-38 | 14223 | 1552 | 1066 | 258 |
| Organism Function | Organism Function | biological regulation | 2.56E-33 | 1.83E-35 | 14223 | 6921 | 1066 | 712 |
| Organism Function | Organism Function | multi-organism process | 2.34E-31 | 1.82E-33 | 14223 | 790 | 1066 | 161 |

| | | | | | | | | |
|---|---|---|---|---|---|---|---|---|
| Organism Function | Organism Function | regulation of biological process | 3.80E-30 | 3.27E-32 | 14223 | 6533 | 1066 | 674 |
| Organism Function | Organism Function | locomotory behavior | 8.09E-30 | 7.31E-32 | 14223 | 275 | 1066 | 86 |
| Organism Function | Organism Function | behavior | 2.98E-28 | 2.81E-30 | 14223 | 467 | 1066 | 113 |
| Organism Function | Organism Function | positive regulation of multicellular organismal process | 7.14E-24 | 1.13E-25 | 14223 | 283 | 1066 | 79 |
| Organism Function | Organism Function | response to other organism | 1.91E-17 | 4.86E-19 | 14223 | 362 | 1066 | 80 |
| Organism Function | Organism Function | coagulation | 2.07E-13 | 7.36E-15 | 14223 | 109 | 1066 | 36 |
| Organism Function | Organism Function | blood coagulation | 2.07E-13 | 7.36E-15 | 14223 | 109 | 1066 | 36 |
| Organism Function | Organism Function | homeostatic process | 2.22E-13 | 7.98E-15 | 14223 | 777 | 1066 | 120 |
| Organism Function | Organism Function | hemostasis | 1.26E-12 | 4.80E-14 | 14223 | 115 | 1066 | 36 |
| Organism Function | Organism Function | regulation of body fluid levels | 1.26E-12 | 4.81E-14 | 14223 | 152 | 1066 | 42 |
| Organism Function | Organism Function | interspecies interaction between organisms | 5.50E-12 | 2.28E-13 | 14223 | 327 | 1066 | 65 |
| Organism Function | Organism Function | ovulation cycle | 6.32E-11 | 2.91E-12 | 14223 | 82 | 1066 | 28 |
| Organism Function | Organism Function | negative regulation of multicellular organismal process | 6.42E-11 | 2.96E-12 | 14223 | 177 | 1066 | 43 |
| Organism Function | Organism Function | multicellular organismal macromolecule metabolic process | 7.24E-11 | 3.37E-12 | 14223 | 34 | 1066 | 18 |
| Organism Function | Organism Function | reproductive process | 9.66E-10 | 4.93E-11 | 14223 | 811 | 1066 | 113 |
| Organism Function | Organism Function | reproduction | 1.18E-09 | 6.22E-11 | 14223 | 814 | 1066 | 113 |
| Organism Function | Organism Function | regulation of blood coagulation | 1.79E-08 | 1.11E-09 | 14223 | 40 | 1066 | 17 |
| Organism Function | Organism Function | negative regulation of blood coagulation | 6.74E-08 | 4.87E-09 | 14223 | 25 | 1066 | 13 |
| Organism Function | Organism Function | regulation of coagulation | 8.77E-08 | 6.46E-09 | 14223 | 44 | 1066 | 17 |
| Organism Function | Organism Function | aging | 9.66E-08 | 7.16E-09 | 14223 | 146 | 1066 | 33 |
| Organism Function | Organism Function | rhythmic process | 1.08E-07 | 8.04E-09 | 14223 | 154 | 1066 | 34 |
| Organism Function | Organism Function | death | 1.53E-07 | 1.17E-08 | 14223 | 706 | 1066 | 95 |
| Organism Function | Organism Function | ovulation cycle process | 1.56E-07 | 1.19E-08 | 14223 | 74 | 1066 | 22 |
| Organism Function | Organism Function | regulation of behavior | 2.06E-07 | 1.61E-08 | 14223 | 69 | 1066 | 21 |
| Organism Function | Organism Function | negative regulation of coagulation | 2.07E-07 | 1.63E-08 | 14223 | 27 | 1066 | 13 |
| Organism Function | Organism Function | regulation of system process | 2.41E-07 | 1.91E-08 | 14223 | 315 | 1066 | 53 |
| Organism Function | Organism Function | positive regulation of behavior | 2.59E-07 | 2.08E-08 | 14223 | 47 | 1066 | 17 |
| Organism Function | Organism Function | female gamete generation | 1.02E-06 | 8.78E-08 | 14223 | 69 | 1066 | 20 |
| Organism Function | Organism Function | multicellular organism reproduction | 1.90E-06 | 1.68E-07 | 14223 | 515 | 1066 | 72 |
| Organism Function | Organism Function | reproductive process in a multicellular organism | 1.90E-06 | 1.68E-07 | 14223 | 515 | 1066 | 72 |
| Organism Function | Organism Function | female pregnancy | 2.88E-06 | 2.61E-07 | 14223 | 122 | 1066 | 27 |
| Organism Function | Organism Function | acute-phase response | 6.24E-06 | 5.92E-07 | 14223 | 40 | 1066 | 14 |
| Organism Function | Organism Function | homeostasis of number of cells | 5.86E-05 | 6.64E-06 | 14223 | 96 | 1066 | 21 |
| Organism Function | Organism Function | molting cycle process | 1.04E-04 | 1.27E-05 | 14223 | 44 | 1066 | 13 |
| Organism Function | Organism Function | molting cycle | 1.69E-04 | 2.16E-05 | 14223 | 46 | 1066 | 13 |
| Organism Function | Organism Function | multicellular organismal homeostasis | 5.27E-04 | 7.64E-05 | 14223 | 88 | 1066 | 18 |
| Organism Function | Organism Function | cellular macromolecule metabolic process | 5.29E-04 | 7.67E-05 | 14223 | 3501 | 1066 | 315 |
| Organism Function | Organism Function | oogenesis | 7.97E-04 | 1.21E-04 | 14223 | 40 | 1066 | 11 |
| Organism Function | Organism Function | maternal process involved in female pregnancy | 8.49E-04 | 1.30E-04 | 14223 | 17 | 1066 | 7 |
| Organism Function | Organism Function | blood circulation | 1.17E-03 | 1.90E-04 | 14223 | 181 | 1066 | 28 |
| Organism Function | Organism Function | circulatory system process | 1.17E-03 | 1.90E-04 | 14223 | 181 | 1066 | 28 |
| Organism Function | Organism Function | regulation of fibrinolysis | 1.17E-03 | 1.89E-04 | 14223 | 13 | 1066 | 6 |
| Organism Function | Organism Function | ovulation | 1.21E-03 | 1.99E-04 | 14223 | 18 | 1066 | 7 |
| Organism Function | Organism Function | tissue homeostasis | 1.58E-03 | 2.66E-04 | 14223 | 65 | 1066 | 14 |
| Organism Function | Organism Function | positive regulation of blood coagulation | 1.82E-03 | 3.11E-04 | 14223 | 14 | 1066 | 6 |
| Organism Function | Organism Function | positive regulation of coagulation | 5.22E-03 | 1.05E-03 | 14223 | 17 | 1066 | 6 |
| Organism Function | Organism Function | anatomical structure homeostasis | 5.25E-03 | 1.06E-03 | 14223 | 108 | 1066 | 18 |
| Organism Function | Organism Function | regulation of gonadotropin secretion | 7.32E-03 | 1.59E-03 | 14223 | 4 | 1066 | 3 |
| Organism Function | Organism Function | negative regulation of follicle-stimulating hormone secretion | 7.32E-03 | 1.59E-03 | 14223 | 4 | 1066 | 3 |
| Organism Function | Organism Function | negative regulation of gonadotropin secretion | 7.32E-03 | 1.59E-03 | 14223 | 4 | 1066 | 3 |

| | | | | | | | | |
|---|---|---|---|---|---|---|---|---|
| Organism Function | Organism Function | positive regulation of fibrinolysis | 7.32E-03 | 1.59E-03 | 14223 | 4 | 1066 | 3 |
| Organism Function | Organism Function | regulation of synaptic transmission | 7.37E-03 | 1.60E-03 | 14223 | 130 | 1066 | 20 |
| Organism Function | Organism Function | regulation of transmission of nerve impulse | 7.74E-03 | 1.71E-03 | 14223 | 140 | 1066 | 21 |
| Organism Function | Organism Function | fibrinolysis | 7.74E-03 | 1.72E-03 | 14223 | 8 | 1066 | 4 |
| Organism Function | Organism Function | regulation of symbiosis, encompassing mutualism through parasitism | 7.74E-03 | 1.72E-03 | 14223 | 8 | 1066 | 4 |
| Organism Function | Organism Function | regulation of endocrine process | 1.79E-02 | 4.57E-03 | 14223 | 10 | 1066 | 4 |
| Organism Function | Organism Function | regulation of multi-organism process | 2.31E-02 | 6.37E-03 | 14223 | 46 | 1066 | 9 |
| Organism Function | Organism Function | regulation of muscle adaptation | 4.18E-02 | 1.30E-02 | 14223 | 13 | 1066 | 4 |
| Organism Function | Organism Function | regulation of transcription, DNA-dependent | 8.96E-01 | 8.68E-01 | 14223 | 1803 | 1066 | 124 |
| Organism Function | Organism Function | system process | 9.85E-01 | 9.73E-01 | 14223 | 1505 | 1066 | 95 |
| Regeneration | Cell Differentiation | regulation of cell differentiation | 1.68E-25 | 2.22E-27 | 14223 | 554 | 1066 | 120 |
| Regeneration | Cell Differentiation | positive regulation of cell differentiation | 3.00E-25 | 4.04E-27 | 14223 | 258 | 1066 | 77 |
| Regeneration | Cell Differentiation | cell differentiation | 6.66E-25 | 9.24E-27 | 14223 | 1672 | 1066 | 244 |
| Regeneration | Cell Differentiation | cell development | 2.52E-12 | 9.94E-14 | 14223 | 637 | 1066 | 102 |
| Regeneration | Cell Differentiation | hemopoiesis | 5.49E-11 | 2.50E-12 | 14223 | 234 | 1066 | 51 |
| Regeneration | Cell Differentiation | leukocyte differentiation | 1.36E-08 | 8.32E-10 | 14223 | 128 | 1066 | 32 |
| Regeneration | Cell Differentiation | regulation of myeloid cell differentiation | 5.41E-08 | 3.79E-09 | 14223 | 76 | 1066 | 23 |
| Regeneration | Cell Differentiation | negative regulation of cell differentiation | 5.73E-08 | 4.04E-09 | 14223 | 235 | 1066 | 45 |
| Regeneration | Cell Differentiation | regulation of myeloid leukocyte differentiation | 5.75E-08 | 4.07E-09 | 14223 | 48 | 1066 | 18 |
| Regeneration | Cell Differentiation | positive regulation of myeloid cell differentiation | 5.75E-08 | 4.08E-09 | 14223 | 38 | 1066 | 16 |
| Regeneration | Cell Differentiation | myeloid cell differentiation | 2.41E-07 | 1.92E-08 | 14223 | 95 | 1066 | 25 |
| Regeneration | Cell Differentiation | neuron differentiation | 2.71E-07 | 2.20E-08 | 14223 | 443 | 1066 | 67 |
| Regeneration | Cell Differentiation | positive regulation of osteoblast differentiation | 1.22E-06 | 1.06E-07 | 14223 | 26 | 1066 | 12 |
| Regeneration | Cell Differentiation | regulation of osteoblast differentiation | 2.25E-06 | 2.02E-07 | 14223 | 48 | 1066 | 16 |
| Regeneration | Cell Differentiation | myeloid leukocyte differentiation | 3.13E-06 | 2.84E-07 | 14223 | 38 | 1066 | 14 |
| Regeneration | Cell Differentiation | striated muscle cell differentiation | 3.18E-06 | 2.90E-07 | 14223 | 87 | 1066 | 22 |
| Regeneration | Cell Differentiation | regulation of lymphocyte differentiation | 5.48E-06 | 5.17E-07 | 14223 | 57 | 1066 | 17 |
| Regeneration | Cell Differentiation | cell morphogenesis involved in differentiation | 1.48E-05 | 1.51E-06 | 14223 | 241 | 1066 | 40 |
| Regeneration | Cell Differentiation | positive regulation of myeloid leukocyte differentiation | 5.07E-05 | 5.69E-06 | 14223 | 20 | 1066 | 9 |
| Regeneration | Cell Differentiation | hemopoietic progenitor cell differentiation | 6.33E-05 | 7.25E-06 | 14223 | 16 | 1066 | 8 |
| Regeneration | Cell Differentiation | regulation of cell morphogenesis involved in differentiation | 1.00E-04 | 1.21E-05 | 14223 | 92 | 1066 | 20 |
| Regeneration | Cell Differentiation | positive regulation of macrophage derived foam cell differentiation | 1.20E-04 | 1.50E-05 | 14223 | 13 | 1066 | 7 |
| Regeneration | Cell Differentiation | neural crest cell differentiation | 1.32E-04 | 1.65E-05 | 14223 | 33 | 1066 | 11 |
| Regeneration | Cell Differentiation | negative regulation of myeloid leukocyte differentiation | 1.75E-04 | 2.25E-05 | 14223 | 23 | 1066 | 9 |
| Regeneration | Cell Differentiation | regulation of macrophage differentiation | 2.13E-04 | 2.82E-05 | 14223 | 10 | 1066 | 6 |
| Regeneration | Cell Differentiation | negative regulation of myeloid cell differentiation | 2.33E-04 | 3.10E-05 | 14223 | 35 | 1066 | 11 |
| Regeneration | Cell Differentiation | epithelial cell differentiation | 3.61E-04 | 5.05E-05 | 14223 | 168 | 1066 | 28 |
| Regeneration | Cell Differentiation | regulation of macrophage derived foam cell differentiation | 4.89E-04 | 7.01E-05 | 14223 | 26 | 1066 | 9 |
| Regeneration | Cell Differentiation | regulation of osteoclast differentiation | 4.89E-04 | 7.01E-05 | 14223 | 26 | 1066 | 9 |
| Regeneration | Cell Differentiation | lymphocyte differentiation | 5.37E-04 | 7.81E-05 | 14223 | 96 | 1066 | 19 |
| Regeneration | Cell Differentiation | glial cell differentiation | 8.49E-04 | 1.30E-04 | 14223 | 61 | 1066 | 14 |
| Regeneration | Cell Differentiation | regulation of T cell differentiation | 1.04E-03 | 1.64E-04 | 14223 | 48 | 1066 | 12 |
| Regeneration | Cell Differentiation | negative regulation of osteoclast differentiation | 1.17E-03 | 1.89E-04 | 14223 | 13 | 1066 | 6 |
| Regeneration | Cell Differentiation | positive regulation of erythrocyte differentiation | 1.17E-03 | 1.89E-04 | 14223 | 13 | 1066 | 6 |
| Regeneration | Cell Differentiation | myoblast differentiation | 1.21E-03 | 1.99E-04 | 14223 | 18 | 1066 | 7 |
| Regeneration | Cell Differentiation | macrophage differentiation | 1.37E-03 | 2.29E-04 | 14223 | 9 | 1066 | 5 |
| Regeneration | Cell Differentiation | regulation of granulocyte differentiation | 1.37E-03 | 2.29E-04 | 14223 | 9 | 1066 | 5 |
| Regeneration | Cell Differentiation | regulation of epithelial to mesenchymal transition | 1.82E-03 | 3.11E-04 | 14223 | 14 | 1066 | 6 |
| Regeneration | Cell Differentiation | B cell differentiation | 2.16E-03 | 3.77E-04 | 14223 | 45 | 1066 | 11 |
| Regeneration | Cell Differentiation | positive regulation of macrophage differentiation | 2.34E-03 | 4.16E-04 | 14223 | 6 | 1066 | 4 |

| | | | | | | | | |
|---|---|---|---|---|---|---|---|---|
| Regeneration | Cell Differentiation | positive regulation of lymphocyte differentiation | 2.34E-03 | 4.18E-04 | 14223 | 32 | 1066 | 9 |
| Regeneration | Cell Differentiation | negative regulation of lymphocyte differentiation | 2.64E-03 | 4.85E-04 | 14223 | 15 | 1066 | 6 |
| Regeneration | Cell Differentiation | oocyte differentiation | 4.20E-03 | 8.16E-04 | 14223 | 22 | 1066 | 7 |
| Regeneration | Cell Differentiation | smooth muscle cell differentiation | 4.62E-03 | 9.14E-04 | 14223 | 7 | 1066 | 4 |
| Regeneration | Cell Differentiation | regulation of monocyte differentiation | 4.62E-03 | 9.14E-04 | 14223 | 7 | 1066 | 4 |
| Regeneration | Cell Differentiation | negative regulation of granulocyte differentiation | 4.62E-03 | 9.14E-04 | 14223 | 7 | 1066 | 4 |
| Regeneration | Cell Differentiation | regulation of neuron differentiation | 4.83E-03 | 9.60E-04 | 14223 | 171 | 1066 | 25 |
| Regeneration | Cell Differentiation | cell morphogenesis involved in neuron differentiation | 5.19E-03 | 1.03E-03 | 14223 | 191 | 1066 | 27 |
| Regeneration | Cell Differentiation | osteoblast differentiation | 5.27E-03 | 1.07E-03 | 14223 | 36 | 1066 | 9 |
| Regeneration | Cell Differentiation | astrocyte differentiation | 5.77E-03 | 1.19E-03 | 14223 | 12 | 1066 | 5 |
| Regeneration | Cell Differentiation | regulation of B cell differentiation | 5.77E-03 | 1.19E-03 | 14223 | 12 | 1066 | 5 |
| Regeneration | Cell Differentiation | cell maturation | 7.32E-03 | 1.58E-03 | 14223 | 77 | 1066 | 14 |
| Regeneration | Cell Differentiation | oocyte maturation | 8.11E-03 | 1.81E-03 | 14223 | 13 | 1066 | 5 |
| Regeneration | Cell Differentiation | mesodermal cell differentiation | 1.23E-02 | 2.91E-03 | 14223 | 9 | 1066 | 4 |
| Regeneration | Cell Differentiation | regulation of erythrocyte differentiation | 1.47E-02 | 3.56E-03 | 14223 | 21 | 1066 | 6 |
| Regeneration | Cell Differentiation | cell fate commitment | 4.18E-02 | 1.29E-02 | 14223 | 136 | 1066 | 18 |
| Regeneration | Development | system development | 4.87E-51 | 9.21E-54 | 14223 | 2427 | 1066 | 382 |
| Regeneration | Development | anatomical structure development | 4.89E-51 | 1.03E-53 | 14223 | 2660 | 1066 | 405 |
| Regeneration | Development | organ development | 6.92E-50 | 1.74E-52 | 14223 | 1800 | 1066 | 314 |
| Regeneration | Development | developmental process | 9.32E-45 | 3.33E-47 | 14223 | 3236 | 1066 | 445 |
| Regeneration | Development | multicellular organismal development | 1.62E-44 | 6.12E-47 | 14223 | 2973 | 1066 | 420 |
| Regeneration | Development | regulation of developmental process | 2.19E-38 | 1.20E-40 | 14223 | 794 | 1066 | 174 |
| Regeneration | Development | positive regulation of developmental process | 6.36E-34 | 4.28E-36 | 14223 | 351 | 1066 | 104 |
| Regeneration | Development | tissue development | 8.25E-34 | 5.72E-36 | 14223 | 755 | 1066 | 161 |
| Regeneration | Development | cellular developmental process | 6.11E-24 | 9.37E-26 | 14223 | 1718 | 1066 | 246 |
| Regeneration | Development | anatomical structure morphogenesis | 5.47E-23 | 9.88E-25 | 14223 | 1219 | 1066 | 192 |
| Regeneration | Development | regulation of anatomical structure morphogenesis | 1.35E-20 | 2.95E-22 | 14223 | 303 | 1066 | 77 |
| Regeneration | Development | organ morphogenesis | 9.65E-20 | 2.19E-21 | 14223 | 636 | 1066 | 119 |
| Regeneration | Development | tube development | 2.09E-17 | 5.35E-19 | 14223 | 297 | 1066 | 71 |
| Regeneration | Development | regulation of growth | 6.86E-17 | 1.80E-18 | 14223 | 362 | 1066 | 79 |
| Regeneration | Development | immune system development | 1.36E-15 | 3.88E-17 | 14223 | 276 | 1066 | 65 |
| Regeneration | Development | tissue morphogenesis | 4.38E-15 | 1.28E-16 | 14223 | 275 | 1066 | 64 |
| Regeneration | Development | hemopoietic or lymphoid organ development | 1.45E-14 | 4.50E-16 | 14223 | 260 | 1066 | 61 |
| Regeneration | Development | nervous system development | 3.11E-14 | 1.03E-15 | 14223 | 1158 | 1066 | 162 |
| Regeneration | Development | morphogenesis of a branching epithelium | 6.79E-14 | 2.30E-15 | 14223 | 100 | 1066 | 35 |
| Regeneration | Development | growth | 1.52E-13 | 5.34E-15 | 14223 | 209 | 1066 | 52 |
| Regeneration | Development | morphogenesis of a branching structure | 2.33E-13 | 8.42E-15 | 14223 | 121 | 1066 | 38 |
| Regeneration | Development | regulation of cell growth | 1.62E-12 | 6.23E-14 | 14223 | 214 | 1066 | 51 |
| Regeneration | Development | branching morphogenesis of a tube | 3.72E-12 | 1.51E-13 | 14223 | 90 | 1066 | 31 |
| Regeneration | Development | regulation of anatomical structure size | 5.46E-12 | 2.25E-13 | 14223 | 319 | 1066 | 64 |
| Regeneration | Development | negative regulation of developmental process | 7.62E-12 | 3.18E-13 | 14223 | 290 | 1066 | 60 |
| Regeneration | Development | epidermis development | 1.38E-11 | 5.86E-13 | 14223 | 190 | 1066 | 46 |
| Regeneration | Development | anatomical structure formation involved in morphogenesis | 1.73E-11 | 7.48E-13 | 14223 | 376 | 1066 | 70 |
| Regeneration | Development | reproductive developmental process | 2.17E-11 | 9.43E-13 | 14223 | 297 | 1066 | 60 |
| Regeneration | Development | morphogenesis of an epithelium | 2.32E-11 | 1.02E-12 | 14223 | 207 | 1066 | 48 |
| Regeneration | Development | epithelium development | 2.52E-11 | 1.11E-12 | 14223 | 338 | 1066 | 65 |
| Regeneration | Development | neurogenesis | 4.11E-11 | 1.83E-12 | 14223 | 639 | 1066 | 99 |
| Regeneration | Development | ectoderm development | 7.04E-11 | 3.27E-12 | 14223 | 206 | 1066 | 47 |
| Regeneration | Development | gland development | 1.32E-10 | 6.25E-12 | 14223 | 195 | 1066 | 45 |
| Regeneration | Development | ossification | 2.79E-10 | 1.36E-11 | 14223 | 117 | 1066 | 33 |

| | | | | | | | | |
|---|---|---|---|---|---|---|---|---|
| Regeneration | Development | generation of neurons | 2.96E-10 | 1.45E-11 | 14223 | 596 | 1066 | 92 |
| Regeneration | Development | tube morphogenesis | 3.82E-10 | 1.88E-11 | 14223 | 201 | 1066 | 45 |
| Regeneration | Development | bone development | 4.51E-10 | 2.26E-11 | 14223 | 132 | 1066 | 35 |
| Regeneration | Development | mammary gland development | 2.15E-09 | 1.19E-10 | 14223 | 88 | 1066 | 27 |
| Regeneration | Development | sex differentiation | 2.75E-09 | 1.54E-10 | 14223 | 176 | 1066 | 40 |
| Regeneration | Development | regulation of ossification | 6.49E-09 | 3.76E-10 | 14223 | 86 | 1066 | 26 |
| Regeneration | Development | development of primary sexual characteristics | 9.51E-09 | 5.67E-10 | 14223 | 147 | 1066 | 35 |
| Regeneration | Development | developmental growth | 1.23E-08 | 7.39E-10 | 14223 | 114 | 1066 | 30 |
| Regeneration | Development | ureteric bud development | 1.48E-08 | 9.15E-10 | 14223 | 60 | 1066 | 21 |
| Regeneration | Development | female sex differentiation | 1.88E-08 | 1.18E-09 | 14223 | 84 | 1066 | 25 |
| Regeneration | Development | gland morphogenesis | 3.18E-08 | 2.12E-09 | 14223 | 80 | 1066 | 24 |
| Regeneration | Development | reproductive structure development | 5.01E-08 | 3.46E-09 | 14223 | 164 | 1066 | 36 |
| Regeneration | Development | lung development | 6.33E-08 | 4.53E-09 | 14223 | 102 | 1066 | 27 |
| Regeneration | Development | development of primary female sexual characteristics | 1.16E-07 | 8.70E-09 | 14223 | 79 | 1066 | 23 |
| Regeneration | Development | respiratory tube development | 1.19E-07 | 8.98E-09 | 14223 | 105 | 1066 | 27 |
| Regeneration | Development | female gonad development | 1.20E-07 | 9.01E-09 | 14223 | 73 | 1066 | 22 |
| Regeneration | Development | respiratory system development | 1.53E-07 | 1.16E-08 | 14223 | 113 | 1066 | 28 |
| Regeneration | Development | positive regulation of growth | 1.74E-07 | 1.35E-08 | 14223 | 87 | 1066 | 24 |
| Regeneration | Development | positive regulation of ossification | 2.07E-07 | 1.63E-08 | 14223 | 27 | 1066 | 13 |
| Regeneration | Development | regulation of muscle organ development | 2.25E-07 | 1.78E-08 | 14223 | 52 | 1066 | 18 |
| Regeneration | Development | odontogenesis | 2.64E-07 | 2.14E-08 | 14223 | 64 | 1066 | 20 |
| Regeneration | Development | exocrine system development | 2.87E-07 | 2.34E-08 | 14223 | 37 | 1066 | 15 |
| Regeneration | Development | regulation of cell development | 3.99E-07 | 3.29E-08 | 14223 | 251 | 1066 | 45 |
| Regeneration | Development | gonad development | 7.76E-07 | 6.58E-08 | 14223 | 129 | 1066 | 29 |
| Regeneration | Development | neuron development | 8.10E-07 | 6.89E-08 | 14223 | 327 | 1066 | 53 |
| Regeneration | Development | embryonic development | 9.84E-07 | 8.39E-08 | 14223 | 604 | 1066 | 82 |
| Regeneration | Development | epithelial tube morphogenesis | 1.64E-06 | 1.43E-07 | 14223 | 126 | 1066 | 28 |
| Regeneration | Development | regulation of biomineral formation | 2.17E-06 | 1.93E-07 | 14223 | 37 | 1066 | 14 |
| Regeneration | Development | regulation of morphogenesis of a branching structure | 3.18E-06 | 2.91E-07 | 14223 | 28 | 1066 | 12 |
| Regeneration | Development | regulation of bone mineralization | 3.30E-06 | 3.04E-07 | 14223 | 33 | 1066 | 13 |
| Regeneration | Development | cartilage development | 6.60E-06 | 6.28E-07 | 14223 | 77 | 1066 | 20 |
| Regeneration | Development | regulation of neuron apoptosis | 6.97E-06 | 6.71E-07 | 14223 | 91 | 1066 | 22 |
| Regeneration | Development | urogenital system development | 7.00E-06 | 6.75E-07 | 14223 | 143 | 1066 | 29 |
| Regeneration | Development | positive regulation of cell growth | 7.22E-06 | 6.97E-07 | 14223 | 52 | 1066 | 16 |
| Regeneration | Development | ureteric bud morphogenesis | 7.41E-06 | 7.19E-07 | 14223 | 30 | 1066 | 12 |
| Regeneration | Development | gliogenesis | 7.46E-06 | 7.25E-07 | 14223 | 71 | 1066 | 19 |
| Regeneration | Development | positive regulation of bone mineralization | 8.98E-06 | 8.81E-07 | 14223 | 21 | 1066 | 10 |
| Regeneration | Development | ovarian follicle development | 9.32E-06 | 9.18E-07 | 14223 | 47 | 1066 | 15 |
| Regeneration | Development | salivary gland morphogenesis | 1.65E-05 | 1.69E-06 | 14223 | 27 | 1066 | 11 |
| Regeneration | Development | regulation of nervous system development | 2.37E-05 | 2.47E-06 | 14223 | 228 | 1066 | 38 |
| Regeneration | Development | positive regulation of biomineral formation | 2.38E-05 | 2.49E-06 | 14223 | 23 | 1066 | 10 |
| Regeneration | Development | lung alveolus development | 2.47E-05 | 2.60E-06 | 14223 | 28 | 1066 | 11 |
| Regeneration | Development | branching involved in ureteric bud morphogenesis | 2.47E-05 | 2.60E-06 | 14223 | 28 | 1066 | 11 |
| Regeneration | Development | salivary gland development | 3.56E-05 | 3.91E-06 | 14223 | 29 | 1066 | 11 |
| Regeneration | Development | mesenchyme development | 3.56E-05 | 3.90E-06 | 14223 | 65 | 1066 | 17 |
| Regeneration | Development | mammary gland morphogenesis | 5.12E-05 | 5.76E-06 | 14223 | 30 | 1066 | 11 |
| Regeneration | Development | central nervous system development | 5.63E-05 | 6.34E-06 | 14223 | 446 | 1066 | 60 |
| Regeneration | Development | placenta development | 7.34E-05 | 8.52E-06 | 14223 | 90 | 1066 | 20 |
| Regeneration | Development | male sex differentiation | 7.74E-05 | 9.03E-06 | 14223 | 83 | 1066 | 19 |
| Regeneration | Development | neural crest cell development | 9.84E-05 | 1.18E-05 | 14223 | 32 | 1066 | 11 |

| | | | | | | | | |
|---|---|---|---|---|---|---|---|---|
| Regeneration | Development | hair follicle development | 1.04E-04 | 1.27E-05 | 14223 | 44 | 1066 | 13 |
| Regeneration | Development | hair cycle process | 1.04E-04 | 1.27E-05 | 14223 | 44 | 1066 | 13 |
| Regeneration | Development | developmental maturation | 1.06E-04 | 1.30E-05 | 14223 | 100 | 1066 | 21 |
| Regeneration | Development | regulation of prostatic bud formation | 1.07E-04 | 1.32E-05 | 14223 | 6 | 1066 | 5 |
| Regeneration | Development | neuron projection development | 1.13E-04 | 1.39E-05 | 14223 | 245 | 1066 | 38 |
| Regeneration | Development | regulation of developmental growth | 1.21E-04 | 1.51E-05 | 14223 | 51 | 1066 | 14 |
| Regeneration | Development | hair cycle | 1.69E-04 | 2.16E-05 | 14223 | 46 | 1066 | 13 |
| Regeneration | Development | branch elongation of an epithelium | 2.13E-04 | 2.82E-05 | 14223 | 10 | 1066 | 6 |
| Regeneration | Development | prostate gland growth | 2.13E-04 | 2.82E-05 | 14223 | 10 | 1066 | 6 |
| Regeneration | Development | in utero embryonic development | 2.57E-04 | 3.46E-05 | 14223 | 218 | 1066 | 34 |
| Regeneration | Development | developmental growth involved in morphogenesis | 2.57E-04 | 3.47E-05 | 14223 | 19 | 1066 | 8 |
| Regeneration | Development | development of primary male sexual characteristics | 2.68E-04 | 3.64E-05 | 14223 | 76 | 1066 | 17 |
| Regeneration | Development | regulation of neurogenesis | 2.83E-04 | 3.85E-05 | 14223 | 210 | 1066 | 33 |
| Regeneration | Development | positive regulation of cell development | 2.92E-04 | 4.00E-05 | 14223 | 84 | 1066 | 18 |
| Regeneration | Development | regulation of embryonic development | 2.94E-04 | 4.02E-05 | 14223 | 30 | 1066 | 10 |
| Regeneration | Development | regulation of cell morphogenesis | 3.04E-04 | 4.16E-05 | 14223 | 149 | 1066 | 26 |
| Regeneration | Development | kidney development | 3.62E-04 | 5.07E-05 | 14223 | 101 | 1066 | 20 |
| Regeneration | Development | embryonic organ development | 5.17E-04 | 7.47E-05 | 14223 | 217 | 1066 | 33 |
| Regeneration | Development | renal system development | 5.37E-04 | 7.81E-05 | 14223 | 104 | 1066 | 20 |
| Regeneration | Development | regulation of kidney development | 7.25E-04 | 1.08E-04 | 14223 | 8 | 1066 | 5 |
| Regeneration | Development | negative regulation of neuron apoptosis | 7.26E-04 | 1.09E-04 | 14223 | 53 | 1066 | 13 |
| Regeneration | Development | pattern specification process | 7.97E-04 | 1.21E-04 | 14223 | 270 | 1066 | 38 |
| Regeneration | Development | nerve development | 8.74E-04 | 1.35E-04 | 14223 | 28 | 1066 | 9 |
| Regeneration | Development | regulation of hair follicle development | 9.46E-04 | 1.48E-04 | 14223 | 5 | 1066 | 4 |
| Regeneration | Development | positive regulation of branching involved in ureteric bud morphogenesis | 9.46E-04 | 1.48E-04 | 14223 | 5 | 1066 | 4 |
| Regeneration | Development | regulation of branching involved in ureteric bud morphogenesis | 9.46E-04 | 1.48E-04 | 14223 | 5 | 1066 | 4 |
| Regeneration | Development | chordate embryonic development | 9.57E-04 | 1.50E-04 | 14223 | 362 | 1066 | 47 |
| Regeneration | Development | negative regulation of growth | 1.00E-03 | 1.57E-04 | 14223 | 126 | 1066 | 22 |
| Regeneration | Development | cellular component morphogenesis | 1.02E-03 | 1.61E-04 | 14223 | 353 | 1066 | 46 |
| Regeneration | Development | regulation of organ formation | 1.08E-03 | 1.72E-04 | 14223 | 23 | 1066 | 8 |
| Regeneration | Development | brain development | 1.15E-03 | 1.84E-04 | 14223 | 305 | 1066 | 41 |
| Regeneration | Development | embryonic morphogenesis | 1.16E-03 | 1.85E-04 | 14223 | 335 | 1066 | 44 |
| Regeneration | Development | tongue development | 1.17E-03 | 1.89E-04 | 14223 | 13 | 1066 | 6 |
| Regeneration | Development | mammary gland alveolus development | 1.17E-03 | 1.89E-04 | 14223 | 13 | 1066 | 6 |
| Regeneration | Development | digestive system development | 1.17E-03 | 1.88E-04 | 14223 | 63 | 1066 | 14 |
| Regeneration | Development | embryonic development ending in birth or egg hatching | 1.19E-03 | 1.94E-04 | 14223 | 366 | 1066 | 47 |
| Regeneration | Development | neuron projection morphogenesis | 1.22E-03 | 2.00E-04 | 14223 | 200 | 1066 | 30 |
| Regeneration | Development | embryonic placenta development | 1.71E-03 | 2.88E-04 | 14223 | 58 | 1066 | 13 |
| Regeneration | Development | prostate gland development | 1.71E-03 | 2.88E-04 | 14223 | 37 | 1066 | 10 |
| Regeneration | Development | regulation of multicellular organism growth | 2.00E-03 | 3.45E-04 | 14223 | 59 | 1066 | 13 |
| Regeneration | Development | regulation of neuron projection development | 2.00E-03 | 3.45E-04 | 14223 | 107 | 1066 | 19 |
| Regeneration | Development | regulation of neurological system process | 2.04E-03 | 3.54E-04 | 14223 | 151 | 1066 | 24 |
| Regeneration | Development | regulation of branching involved in prostate gland morphogenesis | 2.34E-03 | 4.16E-04 | 14223 | 6 | 1066 | 4 |
| Regeneration | Development | sensory organ development | 2.34E-03 | 4.10E-04 | 14223 | 247 | 1066 | 34 |
| Regeneration | Development | regulation of hair cycle | 2.34E-03 | 4.16E-04 | 14223 | 6 | 1066 | 4 |
| Regeneration | Development | exocrine pancreas development | 2.34E-03 | 4.16E-04 | 14223 | 6 | 1066 | 4 |
| Regeneration | Development | lymph node development | 2.38E-03 | 4.29E-04 | 14223 | 10 | 1066 | 5 |
| Regeneration | Development | prostate gland morphogenesis | 2.45E-03 | 4.47E-04 | 14223 | 26 | 1066 | 8 |
| Regeneration | Development | branching involved in mammary gland duct morphogenesis | 2.64E-03 | 4.85E-04 | 14223 | 15 | 1066 | 6 |
| Regeneration | Development | oocyte development | 3.18E-03 | 5.94E-04 | 14223 | 21 | 1066 | 7 |

| | | | | | | | | |
|---|---|---|---|---|---|---|---|---|
| Regeneration | Development | positive regulation of neurogenesis | 3.18E-03 | 5.93E-04 | 14223 | 70 | 1066 | 14 |
| Regeneration | Development | regulation of odontogenesis | 3.86E-03 | 7.38E-04 | 14223 | 11 | 1066 | 5 |
| Regeneration | Development | lung epithelium development | 3.86E-03 | 7.38E-04 | 14223 | 11 | 1066 | 5 |
| Regeneration | Development | smooth muscle tissue development | 3.86E-03 | 7.38E-04 | 14223 | 11 | 1066 | 5 |
| Regeneration | Development | lung morphogenesis | 4.20E-03 | 8.16E-04 | 14223 | 22 | 1066 | 7 |
| Regeneration | Development | mammary gland duct morphogenesis | 4.20E-03 | 8.16E-04 | 14223 | 22 | 1066 | 7 |
| Regeneration | Development | sympathetic nervous system development | 4.62E-03 | 9.14E-04 | 14223 | 7 | 1066 | 4 |
| Regeneration | Development | regulation of bone remodeling | 5.22E-03 | 1.05E-03 | 14223 | 17 | 1066 | 6 |
| Regeneration | Development | regulation of bone resorption | 5.22E-03 | 1.05E-03 | 14223 | 17 | 1066 | 6 |
| Regeneration | Development | negative regulation of ossification | 5.22E-03 | 1.05E-03 | 14223 | 17 | 1066 | 6 |
| Regeneration | Development | branching involved in salivary gland morphogenesis | 5.22E-03 | 1.05E-03 | 14223 | 17 | 1066 | 6 |
| Regeneration | Development | mammary gland epithelium development | 5.27E-03 | 1.07E-03 | 14223 | 36 | 1066 | 9 |
| Regeneration | Development | thymus development | 5.37E-03 | 1.10E-03 | 14223 | 23 | 1066 | 7 |
| Regeneration | Development | germ cell development | 6.00E-03 | 1.24E-03 | 14223 | 92 | 1066 | 16 |
| Regeneration | Development | odontogenesis of dentine-containing tooth | 6.33E-03 | 1.32E-03 | 14223 | 37 | 1066 | 9 |
| Regeneration | Development | prostate gland epithelium morphogenesis | 6.91E-03 | 1.45E-03 | 14223 | 24 | 1066 | 7 |
| Regeneration | Development | anatomical structure maturation | 7.02E-03 | 1.48E-03 | 14223 | 18 | 1066 | 6 |
| Regeneration | Development | regulation of odontogenesis of dentine-containing tooth | 7.32E-03 | 1.59E-03 | 14223 | 4 | 1066 | 3 |
| Regeneration | Development | lung lobe morphogenesis | 7.32E-03 | 1.59E-03 | 14223 | 4 | 1066 | 3 |
| Regeneration | Development | axonogenesis | 7.32E-03 | 1.58E-03 | 14223 | 177 | 1066 | 25 |
| Regeneration | Development | lung lobe development | 7.32E-03 | 1.59E-03 | 14223 | 4 | 1066 | 3 |
| Regeneration | Development | positive regulation of hair follicle development | 7.32E-03 | 1.59E-03 | 14223 | 4 | 1066 | 3 |
| Regeneration | Development | branch elongation involved in mammary gland duct branching | 7.32E-03 | 1.59E-03 | 14223 | 4 | 1066 | 3 |
| Regeneration | Development | male gonad development | 7.62E-03 | 1.66E-03 | 14223 | 61 | 1066 | 12 |
| Regeneration | Development | mesonephros development | 7.74E-03 | 1.72E-03 | 14223 | 8 | 1066 | 4 |
| Regeneration | Development | SMAD protein complex assembly | 7.74E-03 | 1.72E-03 | 14223 | 8 | 1066 | 4 |
| Regeneration | Development | mesodermal cell fate commitment | 7.74E-03 | 1.72E-03 | 14223 | 8 | 1066 | 4 |
| Regeneration | Development | embryonic placenta morphogenesis | 7.74E-03 | 1.72E-03 | 14223 | 8 | 1066 | 4 |
| Regeneration | Development | mesoderm formation | 8.69E-03 | 1.96E-03 | 14223 | 39 | 1066 | 9 |
| Regeneration | Development | regulation of axonogenesis | 9.76E-03 | 2.22E-03 | 14223 | 63 | 1066 | 12 |
| Regeneration | Development | mesoderm morphogenesis | 1.22E-02 | 2.84E-03 | 14223 | 41 | 1066 | 9 |
| Regeneration | Development | pancreas development | 1.22E-02 | 2.84E-03 | 14223 | 41 | 1066 | 9 |
| Regeneration | Development | astrocyte development | 1.23E-02 | 2.91E-03 | 14223 | 9 | 1066 | 4 |
| Regeneration | Development | positive regulation of epidermis development | 1.23E-02 | 2.91E-03 | 14223 | 9 | 1066 | 4 |
| Regeneration | Development | mesoderm development | 1.47E-02 | 3.54E-03 | 14223 | 75 | 1066 | 13 |
| Regeneration | Development | autonomic nervous system development | 1.47E-02 | 3.56E-03 | 14223 | 21 | 1066 | 6 |
| Regeneration | Development | cell fate commitment involved in the formation of primary germ layers | 1.79E-02 | 4.57E-03 | 14223 | 10 | 1066 | 4 |
| Regeneration | Development | regulation of epidermis development | 1.79E-02 | 4.59E-03 | 14223 | 22 | 1066 | 6 |
| Regeneration | Development | gamete generation | 2.19E-02 | 6.00E-03 | 14223 | 409 | 1066 | 45 |
| Regeneration | Development | formation of primary germ layer | 2.31E-02 | 6.37E-03 | 14223 | 46 | 1066 | 9 |
| Regeneration | Development | gastrulation | 4.52E-02 | 1.42E-02 | 14223 | 79 | 1066 | 12 |
| Regeneration | Development | glial cell development | 4.82E-02 | 1.58E-02 | 14223 | 28 | 1066 | 6 |
| Regeneration | Development | sexual reproduction | 5.05E-02 | 1.68E-02 | 14223 | 469 | 1066 | 48 |
| Regeneration | Regeneration | response to wounding | 1.60E-74 | 3.35E-78 | 14223 | 552 | 1066 | 189 |
| Regeneration | Regeneration | wound healing | 1.98E-27 | 2.04E-29 | 14223 | 206 | 1066 | 71 |
| Regeneration | Regeneration | regulation of wound healing | 8.77E-08 | 6.46E-09 | 14223 | 44 | 1066 | 17 |
| Regeneration | Regeneration | regeneration | 6.81E-07 | 5.73E-08 | 14223 | 80 | 1066 | 22 |
| Regeneration | Regeneration | tissue remodeling | 5.67E-05 | 6.42E-06 | 14223 | 54 | 1066 | 15 |
| Regeneration | Regeneration | organ regeneration | 7.93E-05 | 9.42E-06 | 14223 | 37 | 1066 | 12 |
| Regeneration | Regeneration | tissue regeneration | 3.91E-04 | 5.54E-05 | 14223 | 31 | 1066 | 10 |

| | | | | | | | | |
|---|---|---|---|---|---|---|---|---|
| Regeneration | Regeneration | skeletal muscle tissue regeneration | 3.86E-03 | 7.38E-04 | 14223 | 11 | 1066 | 5 |
| Regeneration | Regeneration | regulation of tissue remodeling | 1.17E-02 | 2.71E-03 | 14223 | 20 | 1066 | 6 |
| Regeneration | Stem Cell Function | mesenchymal cell differentiation | 2.58E-05 | 2.73E-06 | 14223 | 57 | 1066 | 16 |
| Regeneration | Stem Cell Function | mesenchymal cell development | 5.67E-05 | 6.42E-06 | 14223 | 54 | 1066 | 15 |
| Regeneration | Stem Cell Function | stem cell maintenance | 1.92E-03 | 3.31E-04 | 14223 | 25 | 1066 | 8 |
| Regeneration | Stem Cell Function | stem cell development | 3.18E-03 | 5.94E-04 | 14223 | 27 | 1066 | 8 |
| Regeneration | Stem Cell Function | stem cell differentiation | 5.27E-03 | 1.07E-03 | 14223 | 36 | 1066 | 9 |
| Structure | Structure | cell adhesion | 1.95E-14 | 6.31E-16 | 14223 | 715 | 1066 | 116 |
| Structure | Structure | biological adhesion | 2.15E-14 | 7.01E-16 | 14223 | 716 | 1066 | 116 |
| Structure | Structure | regulation of cell adhesion | 2.82E-12 | 1.12E-13 | 14223 | 149 | 1066 | 41 |
| Structure | Structure | regulation of binding | 1.31E-09 | 6.94E-11 | 14223 | 201 | 1066 | 44 |
| Structure | Structure | positive regulation of cell adhesion | 1.75E-09 | 9.49E-11 | 14223 | 70 | 1066 | 24 |
| Structure | Structure | positive regulation of binding | 3.98E-09 | 2.24E-10 | 14223 | 109 | 1066 | 30 |
| Structure | Structure | extracellular matrix organization | 1.92E-08 | 1.22E-09 | 14223 | 103 | 1066 | 28 |
| Structure | Structure | leukocyte cell-cell adhesion | 3.18E-06 | 2.91E-07 | 14223 | 28 | 1066 | 12 |
| Structure | Structure | heterophilic cell-cell adhesion | 7.90E-05 | 9.29E-06 | 14223 | 21 | 1066 | 9 |
| Structure | Structure | regulation of cell-cell adhesion | 1.58E-04 | 2.02E-05 | 14223 | 28 | 1066 | 10 |
| Structure | Structure | cell-cell adhesion | 1.01E-03 | 1.58E-04 | 14223 | 293 | 1066 | 40 |
| Structure | Structure | negative regulation of cell adhesion | 1.04E-03 | 1.64E-04 | 14223 | 48 | 1066 | 12 |
| Structure | Structure | cell junction organization | 1.44E-03 | 2.40E-04 | 14223 | 72 | 1066 | 15 |
| Structure | Structure | positive regulation of cell-cell adhesion | 1.82E-03 | 3.11E-04 | 14223 | 14 | 1066 | 6 |
| Structure | Structure | cell adhesion mediated by integrin | 7.74E-03 | 1.72E-03 | 14223 | 8 | 1066 | 4 |
| Structure | Structure | cell junction assembly | 8.48E-03 | 1.91E-03 | 14223 | 54 | 1066 | 11 |

The colors of macroclusters match the corresponding categories in Fig. 3C. N and x denote the total number of reference and tested genes, respectively and nn and xx represent the number of reference and tested genes annotated to a given biological process, respectively.
